# Supplementary material for: Nascent Transcript Folding Plays a Major Role in Determining RNA Polymerase Elongation Rates
Source: Mol Cell. 2020 Aug 6;79(3):488–503.e11. doi: 10.1016/j.molcel.2020.06.002 (PMC7427326; doi:10.1016/j.molcel.2020.06.002)
Supplement: Document S1. Figures S1–S6 and Tables S1 and S2 [file mmc1.pdf]

Molecular Cell, Volume *79*

Supplemental Information

Nascent Transcript Folding Plays a Major Role in Determining RNA Polymerase Elongation Rates

Tomasz W. Turowski, Elisabeth Petfalski, Benjamin D. Goddard, Sarah L. French, Aleksandra Helwak, and David Tollervey

SUPPLEMENTARY FIGURES

Supplementary Figure 1

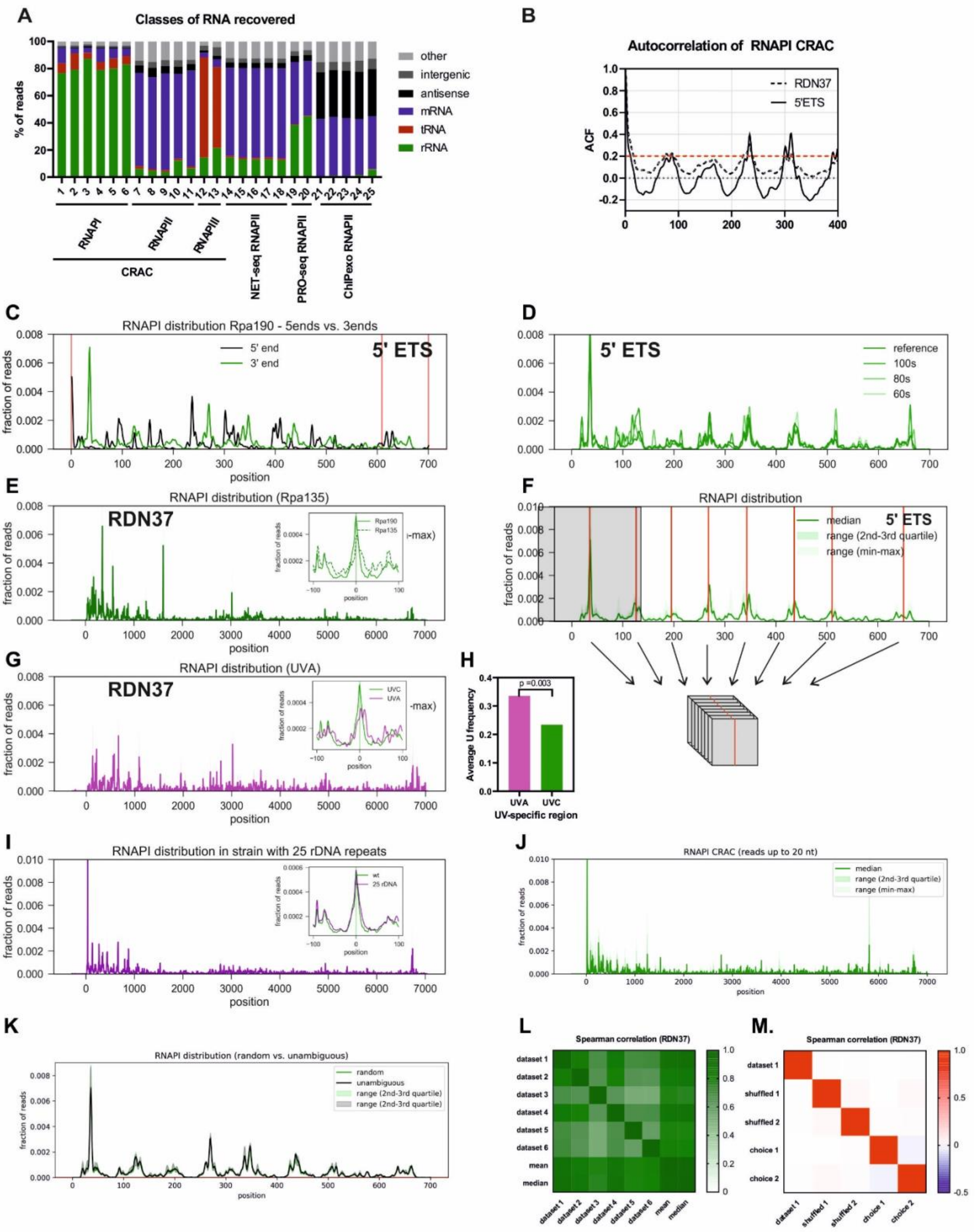

Figure S1. Initial analysis of RNAPI CRAC data (related to Figure 1)

(A) Transcriptome-wide binding profiles for RNAP I, II and III from CRAC (lanes 1 to 13), RNAPII NET-seq (lanes 14-18), PRO-seq (lanes 19-20) and ChIPexo (lanes 21-25).

(B) Autocorrelation function for the 5' ETS region of the pre-rRNA (solid line) and entire *RDN37* transcription unit (dashed line) indicates ~80 nt spacing between peaks in RNAPI CRAC data.

(C) RNAPI CRAC profile over the 5'ETS. The 3' ends (median, green) and the 5' ends (median, black) of mapped RNAs were plotted. There is no clear enrichment of reads at cleavage sites A0 and A1 (red lines).

(D) Rpa190-HTP CRAC profiles for UVC cross-linking time course. The 5' ETS region is presented to show data reproducibility for 60, 80 and 100 seconds of UVC cross-linking time.

(E) Rpa135-HTP CRAC profile, with the second largest RNAPI subunit tagged. Embedded: RNAPI CRAC peak metaplot for *RDN37*, comparing Rpa190 and Rpa135 at the level of single peaks.

(F) Outline of peak metaplot generation. All peaks were found using a dedicated function (see Materials and methods). Windows around each peak were superimposed to generate a metaplot centered around the peaks.

(G) Rpa190-HTP PAR-CRAC (UVA) profile. Embedded: Rpa190-HTP CRAC (UVC) peaks metaplot for *RDN37* overlaid with Rpa190-HTP PAR-CRAC (UVA). The data shows reproducibility on a level of single peaks.

(H) Average U frequency is slightly higher at the 3' end of reads recovered with PAR-CRAC. U frequency was calculated for the last two nucleotides for regions specifically enriched with UVA (purple) or UVC (green). P-value was calculated using a two-sided T-test.

(I) Rpa190-HTP CRAC profile for *RDN37* obtained from strain with 25 rDNA repeats. Embedded: Rpa190-HTP CRAC peaks metaplot for *RDN37* comparing wt and 25 rDNA repeat strains at the level of single peaks.

(J) Rpa190-HTP CRAC profile for *RDN37* obtained from reads only up to 20 nt.

(K) Comparison of Rpa190-HTP CRAC profiles for *RDN37* using random and unambiguous mapping.

(L) Spearman correlation between datasets 1-6, and median or mean of these datasets.

(M) Spearman correlation between dataset 1 and randomized datasets.

Supplementary Figure 2

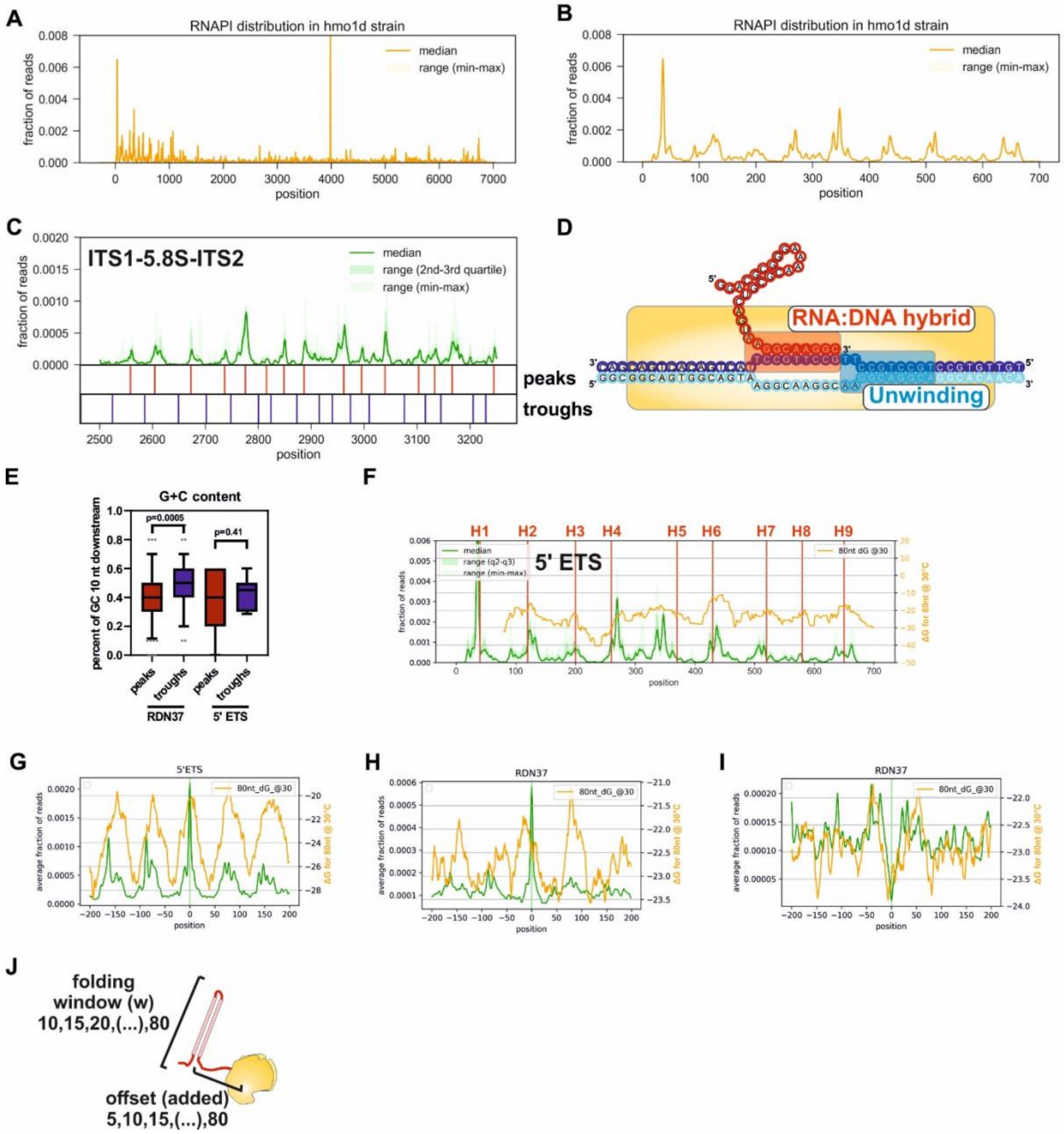

Figure S2. RNAPI density correlates with features in the nascent pre-rRNA (related to Figure 2)

(A) RNAPI CRAC profile for *RDN37* from the Rpa190-HTP *hmo1Δ* strain. The peak at 4000 nt is an artefact previously observed in CRAC analyses.

(B) RNAPI CRAC profile for 5' ETS from the Rpa190-HTP *hmo1Δ* strain.

(C) RNAPI CRAC profile for the ITS1, 5.8S rRNA and ITS2 regions with marked peaks and troughs (identified by peak calling algorithm and) used for further analysis.

(D) Schematic representation of nucleotides considered to play role in RNA:DNA hybrid formation (red box) or DNA unwinding (blue box).

(E) G+C-content in front of RNAPI has a minimal impact on DNA unwinding. Boxplot presenting distribution of G+C-content among peaks and troughs within RDN37 or the 5’ ETS. G+C content is calculated for 10 nt downstream from each feature.

(F) RNAPI CRAC profile (green) for the 5’ ETS and folding of the nascent transcript using different 80 nt folding windows at 30°C. Folding energy was calculated using hybrid-ss-min from the UNAfold package. Red vertical lines represent positions of the known hairpin loops (numbered).

(G) RNAPI CRAC peaks metaplot for the 5’ ETS with folding energy of the nascent transcript (80 nt folding window). *Note*: Stronger structures have lower ΔG.

(H) RNAPI CRAC peaks metaplot for *RDN37* with folding energy of nascent transcript (80 nt folding window).

(I) Same as (H) but troughs were superimposed.

(J) Schematic representation of all parameters tested for calculation of folding window (10-80 nt) and offset (5-80 nt).

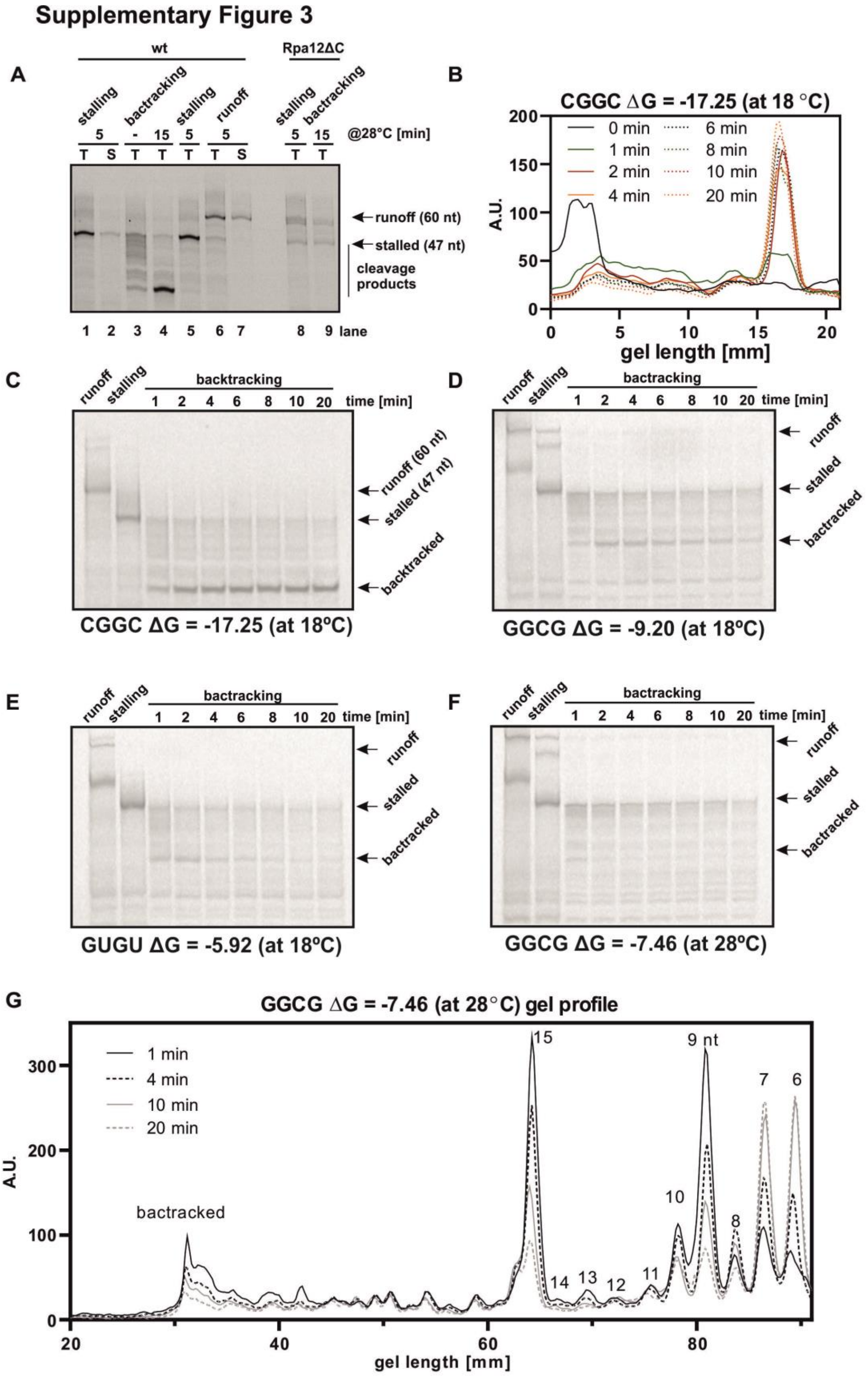

Figure S3. Nascent RNA limits backtracking proportionally to folding energy (related to Figure 3)

(A) RNAPI cleavage requires the Rpa12 C-terminal domain. *In vitro* assay for RNAPI backtracking, performed after initial incubation with transcription buffer (TB) lacking ATP ("stalling"). Nucleotides were washed out and samples incubated in TB without NTPs to induce RNAPI backtracking ("backtracking"). Cleavage products were detectable for RNAPI with wild type Rpa12, but were not observed with Rpa12ΔC. Unexpectedly, the C-terminal truncation of Rpa12 allowed substantial readthrough of the AAA stalling sequence during extension in the absence of added ATP (stalling, lane 8). This observation will be followed up elsewhere.

(B) Quantification of all lanes from Fig. 3E presenting area between stalled and backtracked peaks.

(C) – (F) Representative gels showing backtracking assays used to study kinetics of RNAPI translocation. Assay performed as in Fig. 3C. The scaffold used, temperature and predicted folding energy are indicated below each panel.

(G) Quantification of backtracked peaks and shorter products from backtracking assay. For clarity, only four time points are displayed. The scaffold used, temperature and predicted folding energy are indicated above the plot.

Supplementary Figure 4

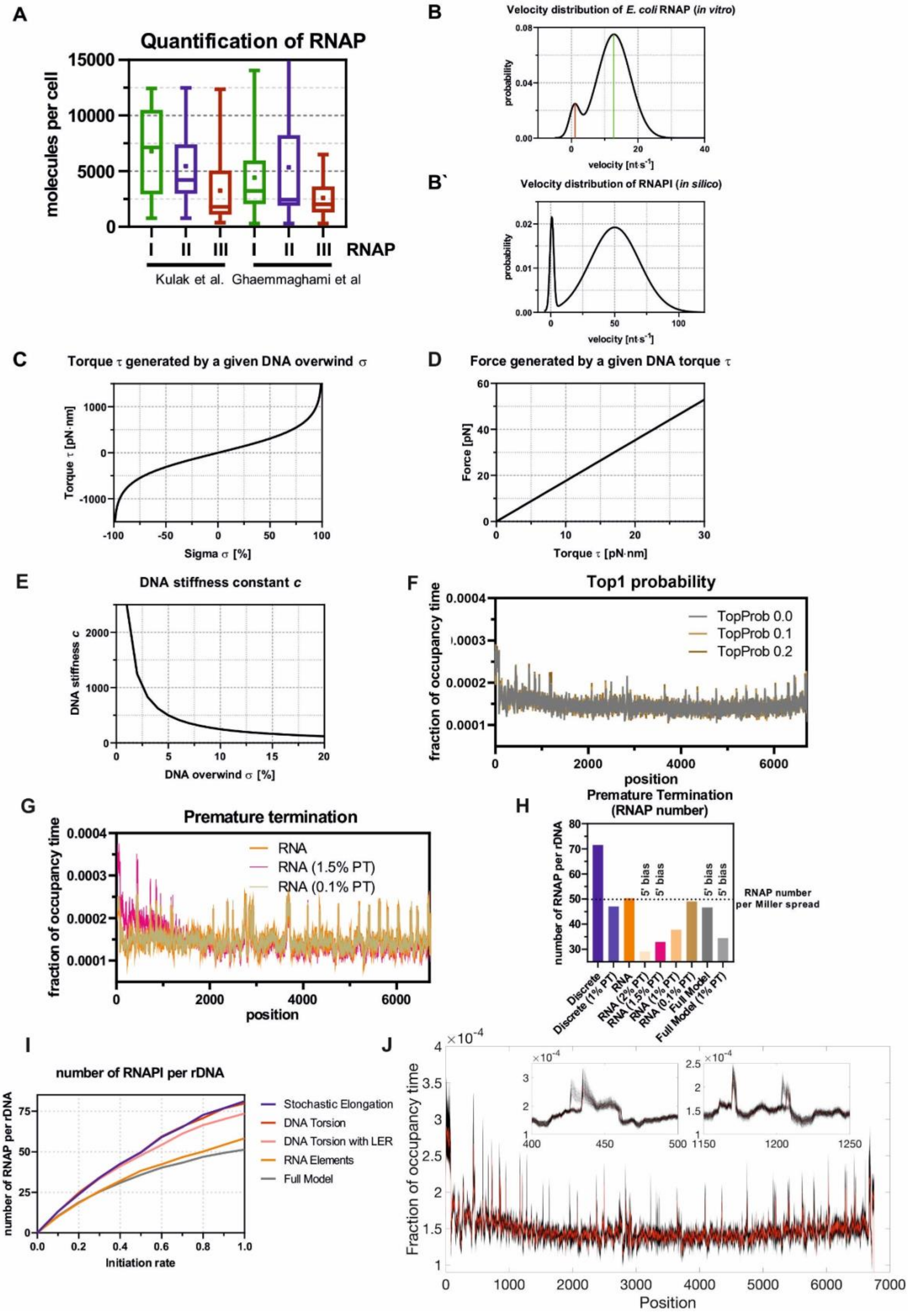

Figure S4. Mathematical model of RNAPI transcription (Related to Figure 4)

A: Quantification of RNA polymerases in yeast cell. Boxplots for each RNA polymerase using two independent studies and all detected subunits.

B: Velocity distribution of bacterial RNAP (Adelman et al., 2002). Described by two Gaussian functions: reflecting 7.8% of the area, represents paused state and is centered at 0.9 nt·s-1 and a second function, which reflects active elongation and is centered at 12.8 nt·s-1.

B': Same as B, but the active elongation function is centered at 50 nt·s-1.

C: Function of DNA torque τ generated by a given DNA overwind σ.

D: Function of force F generated by a given DNA torque τ.

E: Function of DNA stiffness constant c in relationship to DNA overwind σ for V_{Int}=50 nt·s-1.

F: Effect of Top1 (n=64). The results of simulations using parameter TopProb = {0, 0.1, 0.2} were superimposed to show high similarity of overall profile.

G: Effect of premature termination (n=64). The results of simulations allowing for premature termination over first 2000 nt with a given probability = {0%, 0.1%, 1.5%} were superimposed to show high similarity of overall profile.

H: Number of RNAPI molecules per rDNA relative to the premature termination rate (panel Fig. S5G) Note: The 5' bias of the profile (panel I) is accompanied by ~35% drop of RNAP molecules, whereas stable number of RNAPI molecules do not recapitulate the 5' bias (panel J).

I: Number of RNAPI molecules per rDNA relative to the initiation rates are shown for different version of the model used in Figs. 4C-4G.

J: Parameter Fitting (simulations n=256). The results for each parameter set are plotted in (transparent) grey; darker areas indicate more likely results. The red line denotes our choice of parameters (simulations n=1,042). Insets show two zoomed areas. Note that the results are robust under changing parameters, and the results presented for the chosen parameter set are 'typical'.

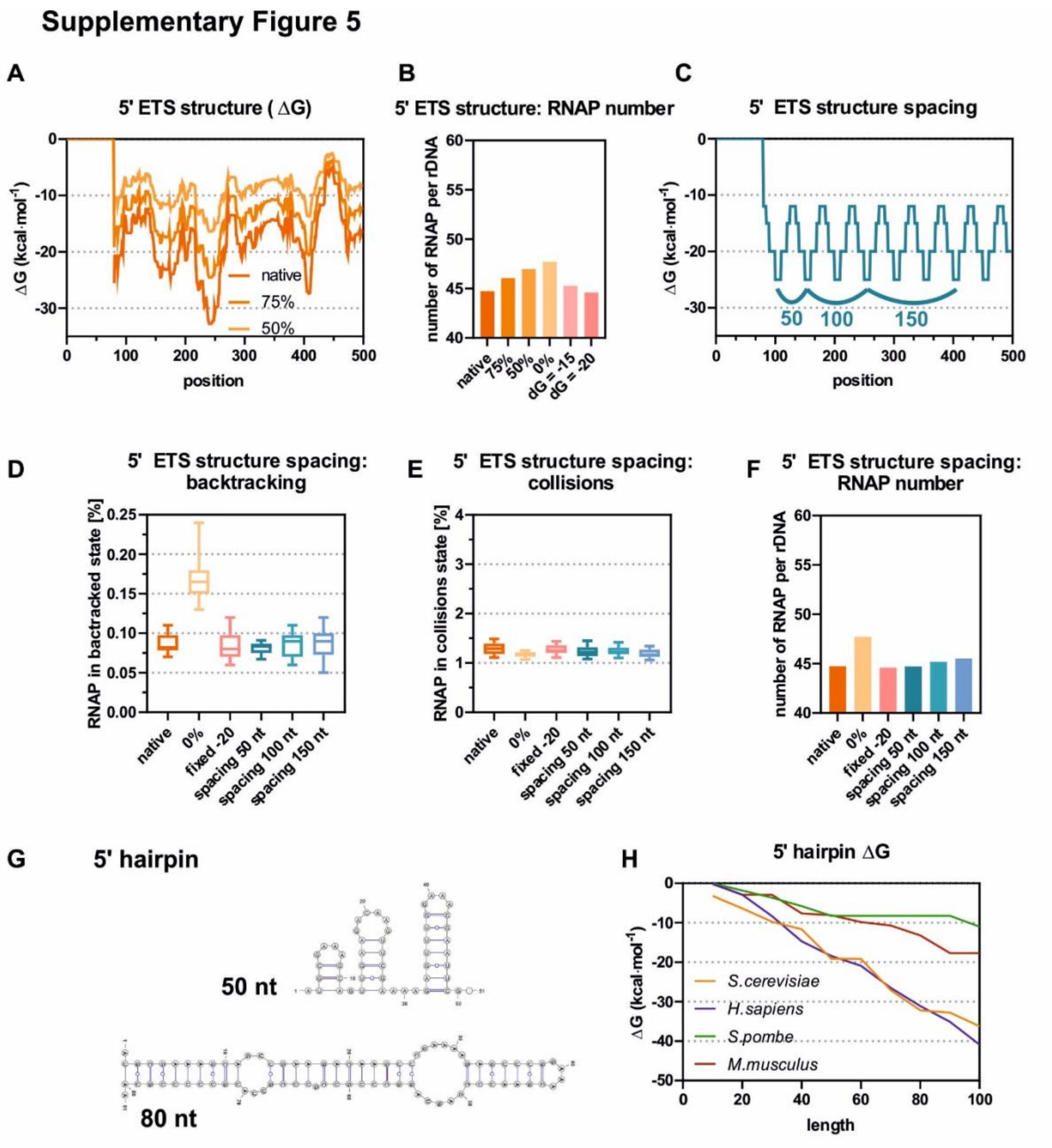

Figure S5. Conclusions from the model on the role of 5’ ETS structure (Related to Figure 5)

A: Plot of the folding input data for the first 500 nt. Folding energy of the 5’ ETS was calculated for a rolling window of 65 nt with 15 nt offset (dark orange). Two modified input files are presented, with folding reduced to 75% or 50% of the biological values. The input with a fixed ΔG would be a straight line and is omitted for clarity.

B: The predicted number of RNAPI molecules loaded on the rDNA remains similar for altered 5’ ETS structures.

C: Overview of altered spacing of 5’ ETS hairpins. Structural data were generated as presented with peak spacing equal 50. For 100 and 150 nt spacing, peaks were connected using longer spacers of fixed energy ΔG = -12 kcal·mol$_{-1}$.

D-F: Modification of spacing between hairpins within the 5'ETS. Measured features of the model remain unaffected: frequency of backtracking (D), frequency of collisions (E) and RNAP number per rDNA unit (F).
G: Secondary structures of the 5' proximal hairpin in the 5' ETS (lower panel) and the 50 nt long transcription intermediate (upper panel).
H: Folding energy of the full-length 5' proximal hairpins and transcription intermediates. The dG was calculated for first 100 nt of the 5' ETS and for shorter intermediates at 10 nt intervals, using sequences from *S. cerevisiae, H. sapiens, S. pombe* and *M. musculus*.

Supplementary Figure 6

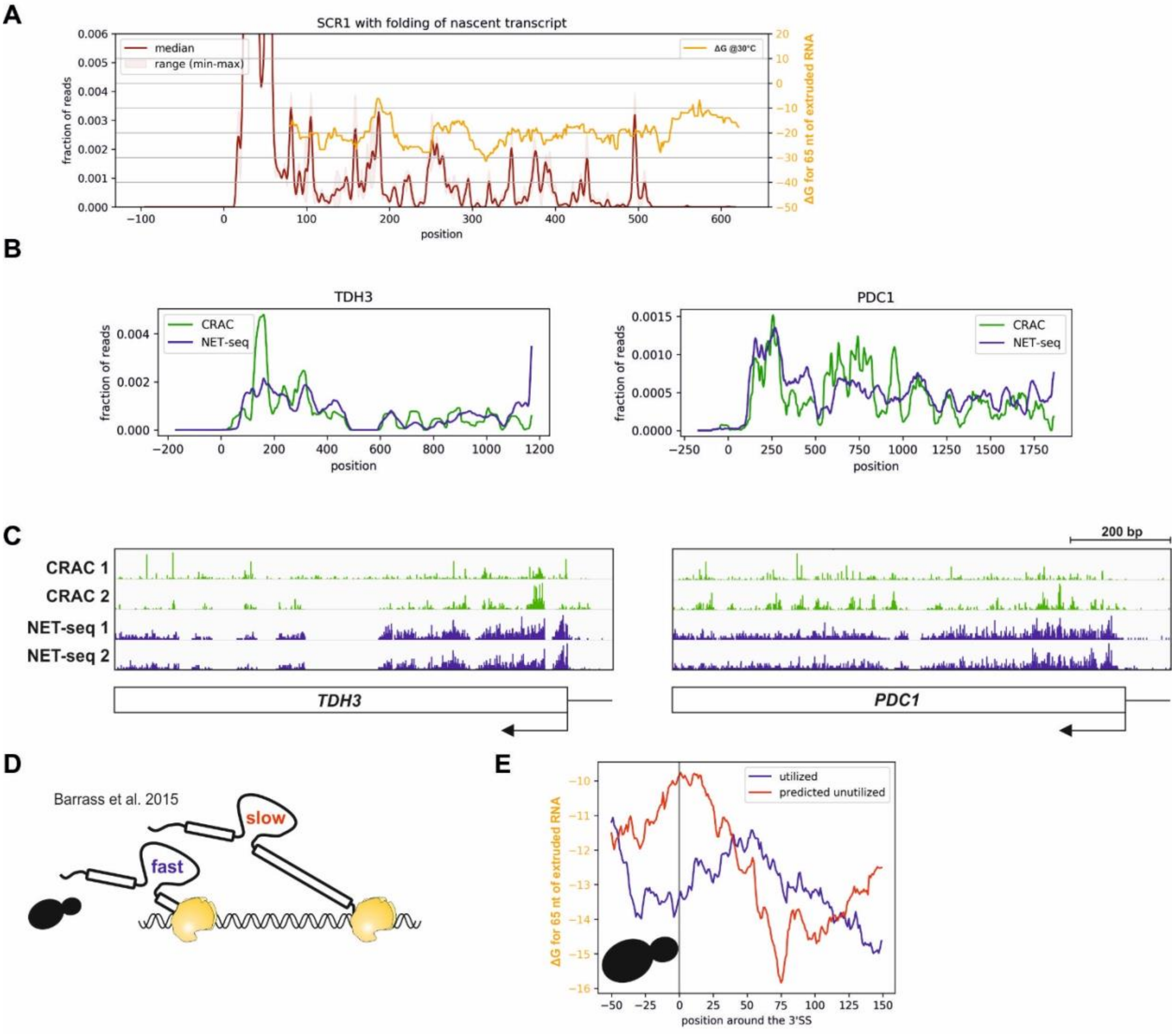

Figure S6. Folding of the nascent transcript plays a role in determining elongation rate of all eukaryotic RNA polymerases (Related to Figure 6)

A: RNAPIII profile for *SCR1* overlaid with nascent RNA folding. The same folding window as for RNAPI analysis was used (65 nt rolling window with 15 nt offset).

B: RNAPII density across *TDH3* (left panel) and *PDC1* genes (right panel). Medians of NET-seq (blue) and CRAC (green) data were plotted to show similarities between the profiles.

C: Genome Browser tracks for RNAPII CRAC (green) and NET-seq (blue) for two highly expressed genes *TDH3* and *PDC1*. Two replicates are shown.

D: Cartoon indicating differences between fast and slow-spliced pre-mRNAs. Slow splicing could be also considered as post transcriptional.

E: Folding energy of nascent RNA around utilized 3’ SS, versus predicted but skipped 3’ SS. The same data were used to generate Fig. 6I. Note that folding of the nascent RNA does not affect accessibility of the 3’ SS.

SUPPLEMENTARY TABLES

Table S1. Fixed parameters used in the mathematical model of RNAPI transcription (related to STAR Methods)

Name	value	Reference
Mean elongation of RNAPI	40 nt s^{-1}	(Kos and Tollervey, 2010)
35S pre-rRNA transcription time	170 s	(Kos and Tollervey, 2010)
rDNA repeats	150-200	(Nomura, 1999)
Active rDNA repeats	75-100	(Toussaint et al., 2005)
RNAPI per rDNA	50±2.5	(Dasgupta et al., 2007; El Hage et al., 2010; French et al., 2003; Hontz et al., 2008; Oakes et al., 2006; Sandmeier et al., 2002; Schneider et al., 2006; Tongaonkar et al., 2005; Viktorovskaya and Schneider, 2015)
35S rDNA length	~7000	SGD
RNAPI footprint	38 nt	(Neyer et al., 2016) PDB 5M5X

Table S2. The velocity of RNA polymerases (related to STAR Methods)

RNA polymerase	Method	Elongation rate nt sec^{-1}	Reference
RNAPII human	single cell microscopy, *in vivo*	115	(Tantale et al., 2016)

RNAPII yeast	single molecule transcription assay, *in vitro*	25-30	(Dangkulwanich et al., 2013)
RNAPII yeast	single molecule transcription assay, *in vitro*	18.7 +/- 2.7	(Lisica et al., 2016)
RNAPI yeast	single molecule transcription assay, *in vitro*	32.2 +/- 2.5	(Lisica et al., 2016)
RNAPI yeast	Metabolic labelling, *in vivo*	40	(Kos and Tollervey, 2010)
RNAPII human	4sUDRB-seq, *in vivo*	30-100	(Fuchs et al., 2014)
RNAPII human	DRB-arrest release, *in vivo*	50	(Cortazar et al., 2019)
RNAPII human	CDK9 inhibition and TT-seq, *in vivo*	39	(Gressel et al., 2017)
RNAPII mouse	Transcription release and GRO-seq, *in vivo*	33	(Jonkers et al., 2014)
