# Supplementary material for: Nascent Transcript Folding Plays a Major Role in Determining RNA Polymerase Elongation Rates
Source: Mol Cell. 2020 Aug 6;79(3):488–503.e11. doi: 10.1016/j.molcel.2020.06.002 (PMC7427326; doi:10.1016/j.molcel.2020.06.002)
Supplement: Document S2. Article plus Supplemental Information [file mmc5.pdf]

Molecular Cell

Article

Nascent Transcript Folding Plays a Major Role in Determining RNA Polymerase Elongation Rates

Graphical Abstract

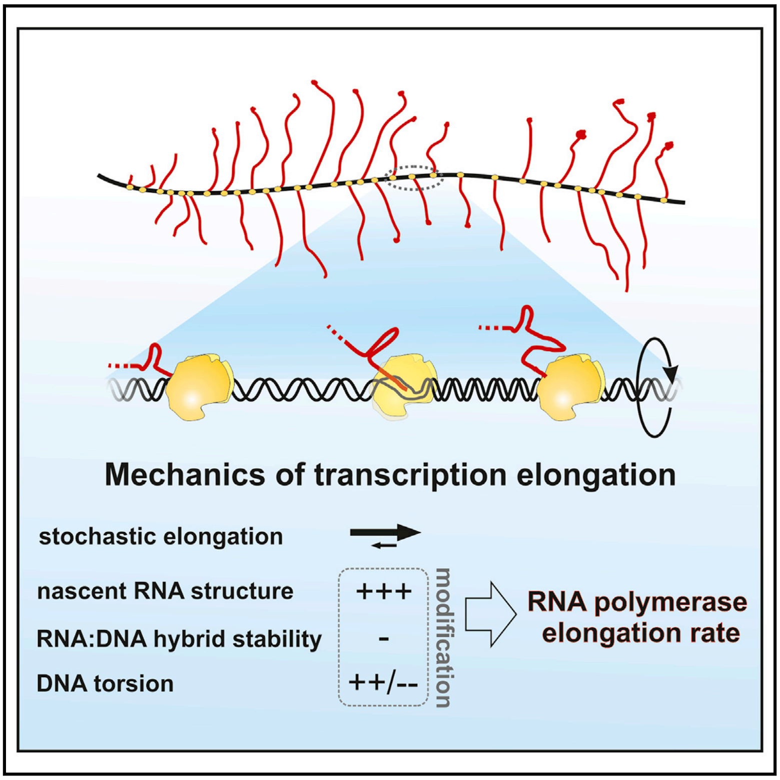

Authors

Tomasz W. Turowski,
Elisabeth Petfalski,
Benjamin D. Goddard, Sarah L. French,
Aleksandra Helwak, David Tollervey

Correspondence

t.turowski@ed.ac.uk (T.W.T.),
d.tollervey@ed.ac.uk (D.T.)

In Brief

Transcription elongation is a stochastic process that can go backward as well as forward. Here Turowski et al. report that folding of the nascent transcript immediately behind the polymerase resists backtracking and promotes forward movement. This results in faster elongation relative to unstructured sequences and is potentially important for cotranscriptional processing.

Highlights

- Structures in the nascent RNA correlate with rapid elongation by RNAPI *in vivo*
- Stable RNA structures limit RNAPI backtracking *in vitro*
- GC content in the transcription bubble tunes transcription elongation rate
- Nascent transcript folding modulates dynamics of all three RNAPs *in vivo*

Turowski et al., 2020, Molecular Cell *79*, 488–503
August 6, 2020 © 2020 The Author(s). Published by Elsevier Inc.
https://doi.org/10.1016/j.molcel.2020.06.002

CellPress

Molecular Cell

Article

Nascent Transcript Folding Plays a Major Role in Determining RNA Polymerase Elongation Rates

Tomasz W. Turowski,[1,*] Elisabeth Petfalski,[1] Benjamin D. Goddard,[2] Sarah L. French,[3] Aleksandra Helwak,[1] and David Tollervey[1,4,*]
[1]Wellcome Centre for Cell Biology, The University of Edinburgh, Edinburgh, UK
[2]School of Mathematics and Maxwell Institute for Mathematical Sciences, The University of Edinburgh, Edinburgh, UK
[3]Department of Microbiology, Immunology, and Cancer Biology, University of Virginia, Charlottesville, VA, USA
[4]Lead Contact
*Correspondence: t.turowski@ed.ac.uk (T.W.T.), d.tollervey@ed.ac.uk (D.T.)
https://doi.org/10.1016/j.molcel.2020.06.002

SUMMARY

Transcription elongation rates influence RNA processing, but sequence-specific regulation is poorly understood. We addressed this *in vivo*, analyzing RNAPI in *S. cerevisiae*. Mapping RNAPI by Miller chromatin spreads or UV crosslinking revealed 5′ enrichment and strikingly uneven local polymerase occupancy along the rDNA, indicating substantial variation in transcription speed. Two features of the nascent transcript correlated with RNAPI distribution: folding energy and GC content in the transcription bubble. *In vitro* experiments confirmed that strong RNA structures close to the polymerase promote forward translocation and limit backtracking, whereas high GC in the transcription bubble slows elongation. A mathematical model for RNAPI elongation confirmed the importance of nascent RNA folding in transcription. RNAPI from *S. pombe* was similarly sensitive to transcript folding, as were *S. cerevisiae* RNAPII and RNAPIII. For RNAPII, unstructured RNA, which favors slowed elongation, was associated with faster cotranscriptional splicing and proximal splice site use, indicating regulatory significance for transcript folding.

INTRODUCTION

Transcription elongation is composed of many successive cycles of nucleotide addition, in which the translocation step is based on Brownian motion without input of external energy. The major driver of transcription elongation is nucleotide addition because pyrophosphate release is essentially irreversible, allowing this step to act as a ratchet (Figure 1A). Dependence on this "Brownian ratchet" rather than an energy-driven processive mechanism makes elongation prone to frequent backtracking and potentially sensitive to inhibition or acceleration by quite modest forces (Dangkulwanich et al., 2013; Guajardo and Sousa, 1997). The rate of RNA polymerase (RNAP) elongation can have marked effects on the fate of the newly transcribed RNA; for example, changing RNA folding patterns or the outcome of alternative splicing (Saldi et al., 2016, 2018). Deep backtracking is relatively rare compared with the number of nucleotide addition cycles but, in aggregate, is widespread in the cell (Sheridan et al., 2019). Despite functional and structural differences, the basic mechanism of transcription elongation has remained the same throughout evolution.

Because of the double-stranded helical structure of DNA, either the DNA or the polymerase must rotate by one complete turn for every 10.5 nt transcribed. In yeast, each active rDNA gene is typically transcribed by ~50 RNAPI molecules, which are associated with nascent pre-ribosomes up to several megadaltons in size. With a transcription rate of ~40 nt s^{-1} (Kos and Tollervey, 2010), the transcribing polymerases are predicted to spin the rDNA at ~240 rpm. If all polymerases transcribe at the same rate, there will be no steric strain between adjacent RNAPI molecules. However, any change in the relative positions of transcribing RNAPI molecules generates substantial torsional stress that can quickly exceed the stalling force of the polymerases (Heberling et al., 2016; Ma et al., 2013; Tantale et al., 2016). The polymerases are therefore torsionally entrained in their relative positions along the rDNA. At the 5′ end, where RNAPI is associated with only a short nascent transcript, we anticipate that torsion can be at least partially released by rotation of the polymerase around the DNA, allowing increased freedom for changes in their relative positions. We therefore predict a gradient of torsional entrainment over the 5′ region of the rDNA. Torsional stress can also be relieved by the action of topoisomerases, Top1 and Top2, which are particularly active on rDNA, reflecting the high transcription rate (Brill et al., 1987; El Hage et al., 2010). However, topoisomerases can unwind a minimum of one complete turn of the DNA, whereas a stalling force is generated by substantially less overwinding for polymerases with spacing typical for the rDNA (120 bp) (Heberling et al., 2016; Ma et al., 2013; Tantale et al., 2016).

 © 2020 The Author(s). Published by Elsevier Inc.
This is an open access article under the CC BY license (http://creativecommons.org/licenses/by/4.0/).

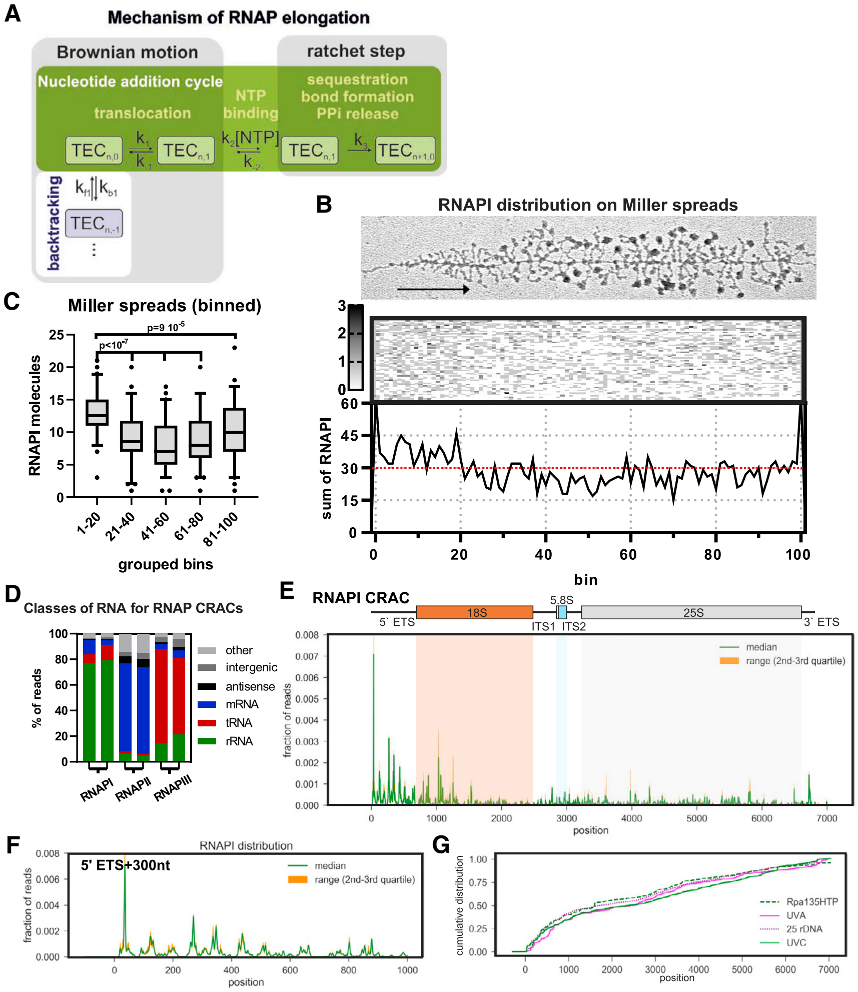

Figure 1. RNAPI Distribution along rDNA *In Vivo*

(A) Schematic of the Brownian ratchet during the nucleotide addition cycle by the transcription elongation complex (TEC). Translocation of RNAP is driven by Brownian motion, which leads to forward (green background) or backward (white background) movement. RNAP pausing is most frequently associated with the $TEC_{n,-1}$ position (i.e., position −1 relative to the 3′ end of nascent RNA) (Dangkulwanich et al., 2014). Directionality is conferred by the ratchet step, where an irreversible phosphodiester bond is formed. Elongation is generated by successive ratchet steps.

(B) RNAPI distribution determined from chromatin spreads. Top panel: illustrative Miller spread (French et al., 2003). An arrow shows transcription start and direction. Center panel: distribution of polymerases along individual spreads with 30–70 RNAPI. Bottom panel: cumulative distribution graph showing the sum of the polymerases in each bin from the genes shown above. The first observed RNAPI molecule is assigned to bin 1 and the last to bin 100.

(C) Boxplot showing the distribution of RNAPI in bins along the rDNA; derived from data in (B). The p values were calculated using a Wilcoxon rank-sum test (n = 60).

(D) Transcriptome-wide binding profiles for the catalytic subunits of RNAPI (Rpa190), RNAPII (Rpb1/Rpo21), and RNAPIII (Rpc160/Rpo31) from replicate CRAC analyses.

(E) Rpa190 CRAC distribution over the *RDN37* gene encoding the pre-rRNA. Top panel: schematic representation of the pre-rRNA transcription unit, including 18S (red), 5.8S (blue), and 25S (gray) rRNA and external and internal transcribed spacers (ETSs and ITSs, respectively). Bottom panel: RNAPI CRAC profile presented as fractions of reads. The solid green line marks the median for six biological replicates, and orange indicates the range between second and third quartiles. The cartoon and graph are approximately aligned with the chromatin spreads in (B). Primary data are included in Table S4.

(F) RNAPI CRAC profiles across the first 1,000 nt of the transcription unit reveal an uneven distribution with apparently regular spacing of peaks.

(G) Cumulative plot of RNAPI distribution profiles for *RDN37* obtained using CRAC with the second largest subunit (Rpa135-HTP), PAR-CRAC using Rpa190-HTP (UVA), CRAC with Rpa190-HTP in a strain with only 25 rDNA copies (25 rDNA), and in the wild type (UVC).

See also Figure S1.

In vivo distributions of RNAPI were initially analyzed using Miller chromatin spreads visualized by electron microscopy (for an example, see Osheim et al., 2009). Subsequently, polymerase distributions have been mapped using techniques that include chromatin immunoprecipitation (ChIP), native elongating transcript sequencing (NET-seq), and crosslinking and analysis of cDNA (CRAC), whereas metabolic labeling approaches such as transient transcriptome sequencing (TT-seq) provide complementary data on polymerase output (Booth et al., 2016; Churchman and Weissman, 2011; Clarke et al., 2018; Drexler et al., 2020; Mayer et al., 2015; Milligan et al., 2016; Nojima et al., 2015; Schwalb et al., 2016; Turowski et al., 2016; Vinayachandran et al., 2018). Commonly, DNA or RNA is recovered in association with the polymerase and identified by sequencing. The frequency of recovery correlates with the polymerase density at each position. Regions with high signals (peaks) are interpreted as having high polymerase occupancy and, therefore, a low elongation rate because RNA transcription is processive. Conversely, troughs reflect low polymerase occupancy and rapid elongation. Notably, all methods that allow high spatial resolution show markedly uneven polymerase distributions along all genes in yeast and human cells.

Mapping at nucleotide resolution should provide mechanistic information on the process of polymerase elongation. RNAPI is ideally suited for these analyses because it has a high transcription rate, transcribes only the nucleosome-free rDNA, and is not known to undergo regulatory phosphorylation (Wittner et al., 2011), facilitating deconvolution of the experimental data. To better understand the mechanism of RNAPI elongation, we mapped transcriptionally engaged RNAPI using CRAC, a method optimized for high specificity of the libraries.

RNAPI elongation rates were integrated with features in the nascent transcript and torsional effects, and we incorporated these results into a kinetic model of RNAPI transcription elongation. This provided mechanistic insights into eukaryotic transcription *in vivo*.

RESULTS

RNAPI Distribution Is Uneven along the Transcription Unit

We initially assessed the distribution of RNAPI along the rDNA transcription units using Miller spreads in a wild-type yeast strain (BY4741) growing in YPD medium, containing 2% glucose + 1 M sorbitol at 30°C, as described previously (Osheim et al., 2009). To analyze RNAPI distribution, we selected 60 spreads for which the full-length rDNA could be unambiguously traced, with polymerases positioned at the 5′ and 3′ ends, and the number of polymerases was around the average number of 50 (range, 30–70 per rDNA repeat) (see STAR Methods for RNAPI quantification). The position of each polymerase along these 60 genes was determined relative to normalized gene length, and the results were combined into 100 bins (1 bin ≈70 bp; Figure 1B). The summary plot of RNAPI distribution showed an excess of polymerase density over the 5′ region of the rDNA (Figures 1B and 1C). This indicated that the average rate of elongation was lower over the 5′ external transcribed spacer (ETS) region, in which major early pre-rRNA assembly events take place (Phipps et al., 2011; Turowski and Tollervey, 2015).

High spatial resolution is not readily obtained using Miller spreads, and we therefore utilized CRAC, a high-resolution UV crosslinking technique. To perform CRAC, the largest subunit of RNAPI, Rpa190, was genomically tagged with hexahistidine (His_6)-tobacco etch virus (TEV) protease cleavage site-2xProtA (HTP). Following growth in SD medium with 2% glucose at 30°C, nascent RNA was covalently crosslinked to RNAPI using 254-nm UV irradiation. After 3-step purification, including stringent denaturing wash conditions, cDNA libraries were prepared and sequenced using Illumina technology. The CRAC protocol used exclusively recovers RNAs with 3′ hydroxyl groups (STAR Methods), expected to represent endogenous 3′ ends of nascent transcripts. Comparing CRAC data for RNAPI with RNAPII and RNAPIII (Figures 1D and S1A) showed predominant recovery of the expected species: rRNA for RNAPI, mRNAs for RNAPII, and tRNAs for RNAPIII (Milligan et al., 2016; Turowski et al., 2016).

Qualitative comparison of the CRAC data with Miller spreads revealed a good match in the overall profile, confirming the 5′ bias (Figures 1B, 1C, and 1E). The average RNAPI density was higher within the first ~1,500 nt, presumably reflecting slower elongation and/or more frequent pausing. This was accompanied by a strikingly uneven distribution of read density over this region (Figure 1F), generating a series of peaks and troughs with apparently regular spacing. Autocorrelation plots (Figures S1B) confirmed a peak separation of around 80 nt, which was very marked over the first 1,000 nt.

Highly uneven polymerase distribution has been observed previously in datasets for RNAPII and RNAPIII (Churchman and Weissman, 2011; Milligan et al., 2016; Turowski et al., 2016). However, the 5′ bias in RNAPI distribution and the presence of such distinct peaks were unexpected. We therefore performed extensive validation of the RNAPI CRAC profile using different crosslinking times, a different RNAPI subunit as bait (Rpa135-HTP), developing photoactivated ribonucleotide (PAR) CRAC based on UVA irradiation and 4-thiouracil labeling, and strains with a decreased number of rDNA repeats (25 rDNA) (Figure S1; see detailed description in STAR Methods). All of these analyses yielded RNAPI distributions that were consistent with the results of CRAC with Rpa190 (Figure 1G). Further analysis was based on the median of six biological replicates, using Rpa190-HTP and UVC (254 nm) crosslinking (Figure S1L).

The strong 5′ peak of RNAPI density was centered around +36 (Figure 1F). The reported RNAPI footprint is ~38 nt, so this is the position expected for a polymerase immediately adjacent to another RNAPI, initiating at +1. We speculate that the +36 peak reflects RNAPI that remains in an initiation state (Engel et al., 2017). Release into an elongation state is expected to increase the elongation rate and might be associated with re-arrangements within the polymerase. In subsequent analyses, we will not consider the 5′ peak, but will focus on elongation steps during RNAPI transcription. Notably, this prominent peak should increase the accuracy of 5′ end positioning in the Miller spreads.

RNAPI Density Correlates with Features in the Nascent pre-rRNA

The ~80 nt spacing of the 5′ ETS peaks does not correspond to the size of the polymerase itself. The footprint of RNAPI is 38 nt, and the minimal spacing between the polymerases on the transcription unit is only slightly longer, as determined by cryoelectron microscopy (cryo-EM) and tomography (Engel et al., 2013; Neyer et al., 2016; Tafur et al., 2016).

We considered that the distribution of RNAPI might be influenced by chromatin structure, as found for RNAPII (Churchman and Weissman, 2011; Milligan et al., 2016). The actively transcribed rDNA repeats are not packaged into nucleosomes but associated with the DNA binding protein Hmo1, which is related to human HMG1 (Hall et al., 2006; Merz et al., 2008; Wittner et al., 2011). However, Rpa190 CRAC performed in an *hmo1Δ* strain still showed a 5′ bias and stable peaks over the 5′ region of the rDNA (Figures S2A and S2B).

High GC Content Moderates the Elongation Rate of RNAPI

We next assessed whether features in the nascent pre-rRNA could affect RNAPI elongation kinetics. A short RNA:DNA hybrid is present in the transcription bubble in the RNAP elongation complex (Figure 2A). For human RNAPII, stable RNA:DNA hybrids in the transcription bubble are more frequently associated with paused or backtracked states (Lukačišin et al., 2017; Schwalb et al., 2016). We used a peak-calling algorithm to define peaks and troughs in the RNAPI density (e.g., Figure S2C) and then determined GC content around each feature (peak or trough). Because the reads are 3′ mapped, the read density indicates the positions of 3′ ends of nascent transcripts within RNAPI. The 10-nt sequence immediately upstream corresponds to the RNA:DNA hybrid forming the transcription bubble (see Figure S2D for a schematic). This 10-nt region showed a higher

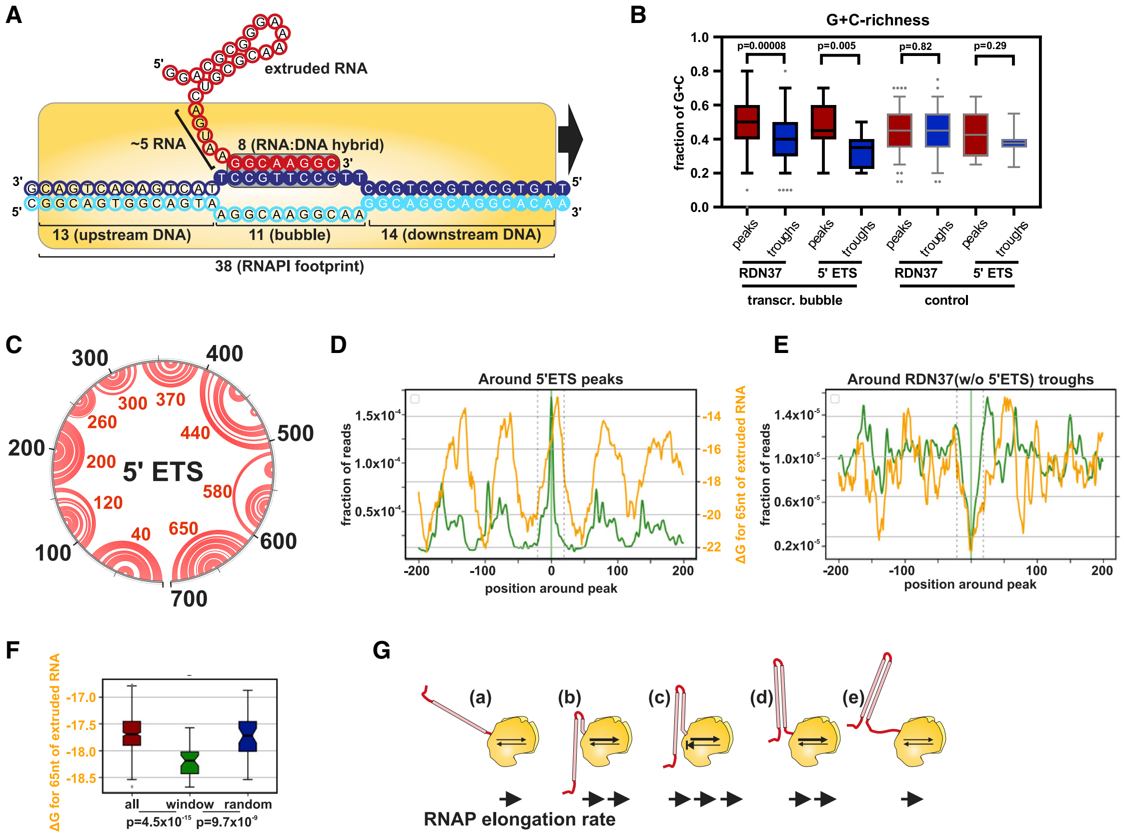

Figure 2. RNAPI Density Correlates with Features in the Nascent pre-rRNA

(A) Schematic representation of RNAPI transcription, including lengths of DNA and RNA elements hidden in the complex. An 8-bp RNA:DNA hybrid forms the transcription bubble, 13 nt of RNA is buried in the RNAPI complex, and 38 bp of DNA is covered by RNAPI. Data were derived from the structure of the RNAPI elongation complex (PDB: 5M5X; Tafur et al., 2016).

(B) GC content of RNA:DNA hybrids. Shown is a boxplot presenting the distribution of GC content among peaks (red) and troughs (blue) within RDN37 (peaks, n = 142; troughs, n = 147) or the 5′ ETS (peaks, n = 14; troughs, n = 12). GC content is calculated for 10 nt upstream of each feature (transcription bubble) or 10 nt upstream plus 10 nt downstream of each feature (control). The p values were calculated using a two-sided t test.

(C) Secondary structure of the 5′ ETS and *in silico* prediction of the UNAfold package (http://unafold.rna.albany.edu/). The gray circle represents positions within the 5′ ETS, and red arches show predicted interactions between bases. Red numbers indicate the loop position for each hairpin. The structure of the 700-nt-long 5′ ETS shows strong regular stems.

(D) RNAPI CRAC peak metaplot for the 5′ ETS with the folding energy of nascent transcript. A folding energy (ΔG in kilocalories per mole) of 65 nt behind RNAPI was predicted and offset by 15 nt. Stronger structures have lower ΔG.

(E) RNAPI CRAC trough metaplot for RDN37 without the 5′ ETS, with folding energy of the nascent transcript. Folding energy as in (D). The area between dashed gray lines was used as the window for the boxplots (F).

(F) Boxplots for (E), comparing distribution of folding energy; full 400-nt window (all), the 40-nt region between the dashed gray lines in (E) (window), or random 40 nt (random). The p values were calculated using a Wilcoxon rank-sum test (n = 90).

(G) Model. Structures forming in the nascent RNA promote transcription elongation by limiting back translocation.

See also Figure S2.

percentage of GC for peaks than for troughs (transcription bubble in Figure 2B), considering the entire rDNA (*RDN37*, $p < 8 \times 10^{-5}$) or the 5′ ETS alone ($p < 5 \times 10^{-3}$). Unwinding of the template DNA in front of the transcription bubble could potentially be slowed by high GC content. However, the first 10 nt downstream of peaks and troughs showed no clear correlation with GC for the 5′ ETS ($p >> 0.05$) or even an opposing trend for *RDN37* ($p = 5 \times 10^{-4}$) (Figure S2E). The GC content for the combined region 10 nt upstream plus 10 nt downstream of each peak and trough (control in Figure 2B; $p >> 0.05$) showed no significant differences.

The data indicate that elevated GC content in the RNA:DNA hybrid in the transcription bubble is associated with increased RNAPI occupancy, presumably reflecting slowed or transiently paused RNAPI.

Folding of the Nascent RNA Promotes RNAPI Elongation

The yeast 5′ ETS folds into 10 stable, extended hairpin structures (Sun et al., 2017; Figure 2C). To examine the influence of RNA structures forming just behind RNAPI, we initially calculated the folding energy for a rolling window of 80 nt upstream of each nucleotide position in the pre-rRNA, corresponding to the

average length of 5′ ETS hairpins. Comparison with the RNAPI CRAC peaks showed an apparent correlation with the predicted folding energy across the 5′ ETS (Figure S2F; $R^{spearman}$ [R^{sp}] = 0.65; because a window of 80 nt is used, the folding energy line commences at +80).

To more systematically compare folding with RNAPI density, we used peak and trough metaplots (Figure S1F). The zero position represents the maximum (Figures S2G and S2H) or minimum (Figure S2I) for the sum of all peaks or troughs identified by the peak-calling algorithm. This revealed a striking correlation where peaks of RNAPI density were associated with weak structures in the nascent pre-rRNA, especially over the 5′ ETS (Figure S2G; R^{sp} = 0.78; structures are plotted as ΔG, with lower values representing greater stability). Conversely, regions of low RNAPI occupancy were correlated with stable structures in the nascent transcript (Figure S2I). Each position on the x axis shows the average folding energy for the nascent transcripts associated with all polymerases located at that distance from the peak (or trough).

To better understand the relationship between pre-rRNA folding and elongation, the analysis was repeated using a range of window sizes to calculate folding energy. In addition, an "offset" was added because the terminal ~15 nt of the transcript is located within the polymerase and unable to participate in folding (Figures S2J). The best correlation was generated by using 65 nt of RNA to calculate folding with a 15-nt offset. The correlation was most marked over the 5′ ETS region (Figure 2D; R^{sp} = 0.53) but was also observed when the *RDN37* gene was analyzed excluding the 5′ ETS (Figures 2E and 2F; $p < 10^{-7}$).

We conclude that weak structures in the nascent pre-rRNA behind RNAPI coincide with sites of slowed elongation (high RNAPI density), whereas strong pre-RNA structures correlate with rapid elongation (low RNAPI density).

Because elongation is driven by Brownian motion, there is the potential for backtracking prior to each nucleotide addition step (Figure 1A). During backtracking, the newly synthesized region of the nascent transcript must re-enter the exit channel of the polymerase. Backtracking should therefore be strongly opposed by formation of a stable RNA structure in the nascent transcript. Moreover, there is a decrease in free energy (i.e., an increase in structure stability) as each additional base pair is formed in extended stems, which might also favor elongation over backtracking. We therefore postulate that stable cotranscriptional folding of nascent pre-rRNA strongly promotes transcription elongation *in vivo* (Figure 2G). This conclusion is supported by single-molecule *in vitro* transcription assays (Tadigotla et al., 2006; Zamft et al., 2012).

The 5′ ETS has very stable overall folding (ΔG −265 kilocalories (kcal) mol^{-1} over 700 nt) relative to the 5′ region of the 18S rRNA (ΔG −220 kcal mol^{-1} over the first 700 nt) despite having low GC content. This suggests that structure in the 5′ ETS may have been selected to promote elongation.

RNA Structures Limit RNAPI Backtracking *In Vitro*

The effects of nascent RNA are expected to operate over 1–2 s because of the fast elongation rate of RNAPI (~40 nt s^{-1}), precluding their experimental determination. To validate the conclusion that the structure in the nascent pre-rRNA limits backtracking, we used an *in vitro* RNAPI transcription system (Pilsl et al., 2016). In this, immobilized RNAPI binds an RNA:DNA scaffold, which mimics the transcription bubble, and elongates the transcript following nucleotide addition. The products are gel separated and visualized using a fluorescent label on the RNA primer (Figure 3A). Within RNAPI, Rpa12 specifically stimulates endonuclease cleavage of nascent RNA in the backtracked position (Kuhn et al., 2007). Backtracking therefore leads to truncation of previously elongated pre-rRNA transcripts.

RNAPI was purified via Rpa135-HTP and bound to immunoglobulin G (IgG)-conjugated magnetic beads to allow rapid exchange of transcription buffer. Nascent transcripts are retained on the beads in association with the polymerase. The template DNA included a sequence that generates a stem-loop structure in the RNA, close to the 5′ end of the transcript. The transcript lacked A residues other than a sequence of three adenines (AAA) close to the 3′ end of the template (Figures 3A and 3B). Incubation for 5 min at 28°C in the presence of nucleotides (GTP, UTP, and CTP) without ATP ([−ATP]) resulted in transcription elongation and stalling at the AAA sequence ("stalled") (Figures 3B and 3C, lanes 1 and 2). Nucleotides were washed out, and the elongation complex was incubated for 15 min at 28°C to allow RNAPI backtracking ("backtracked"). This generated shorter products, observed as a smear on the gel (Figure 3C, lanes 3 and 4). These are due to Rpa12 cleavage of the backtracked transcript, as shown by their absence when the same assay was performed using RNAPI purified from a Rpa12ΔC strain (Lisica et al., 2016), in which Rpa12 lacked the C-terminal domain required for cleavage (Figure S3A, lanes 8 and 9).

Cleavage by Rpa12 should reposition the 3′ end of the nascent transcript in the active site (Lisica et al., 2016; Prescott et al., 2004). Consistent with this expectation, we were able to restart transcription elongation by nucleotide re-addition. Addition of buffer lacking only ATP ([−ATP]) regenerates the stall (stalled), whereas addition of all four nucleotides ([all]) generates the full-length transcript ("runoff") (Figure 3C, lanes 5–7). The full-length runoff product was released by RNAPI into the supernatant fraction (Figure 3C, lane 7).

To compare sequences with different folding energy, we designed *in silico* a construct with four random nucleotides (Figure 3D, top panel). The predicted folding energy of the stalled nascent transcript was calculated, and we selected 10 sequences for experimental analysis, with a range of stabilities (ΔG –5 to –15 kcal mol^{-1} at 28°C; low ΔG corresponds to greater stability). In the backtracking assay, samples were first incubated in [−ATP] transcription buffer to induce stalling and then washed and incubated without nucleotides ([none]) for 15 min at 28°C to allow RNAPI backtracking (Figure 3D, bottom panel). Among the 10 constructs tested, only three generated clear stabilized cleavage products (Figure 3D, lanes 2, 3, and 5). Notably, these correspond to nascent RNAs with the most stable structures (ΔG –12 to –15 kcal mol^{-1}). We predict that this represents the strength of RNA structure needed to efficiently block further backtracking. Moreover, the cleavage product was more abundant for the construct with ΔG –15 than for the constructs with ΔG –12 or –13. These results confirm that stable structures in nascent RNA limit backtracking by RNAPI.

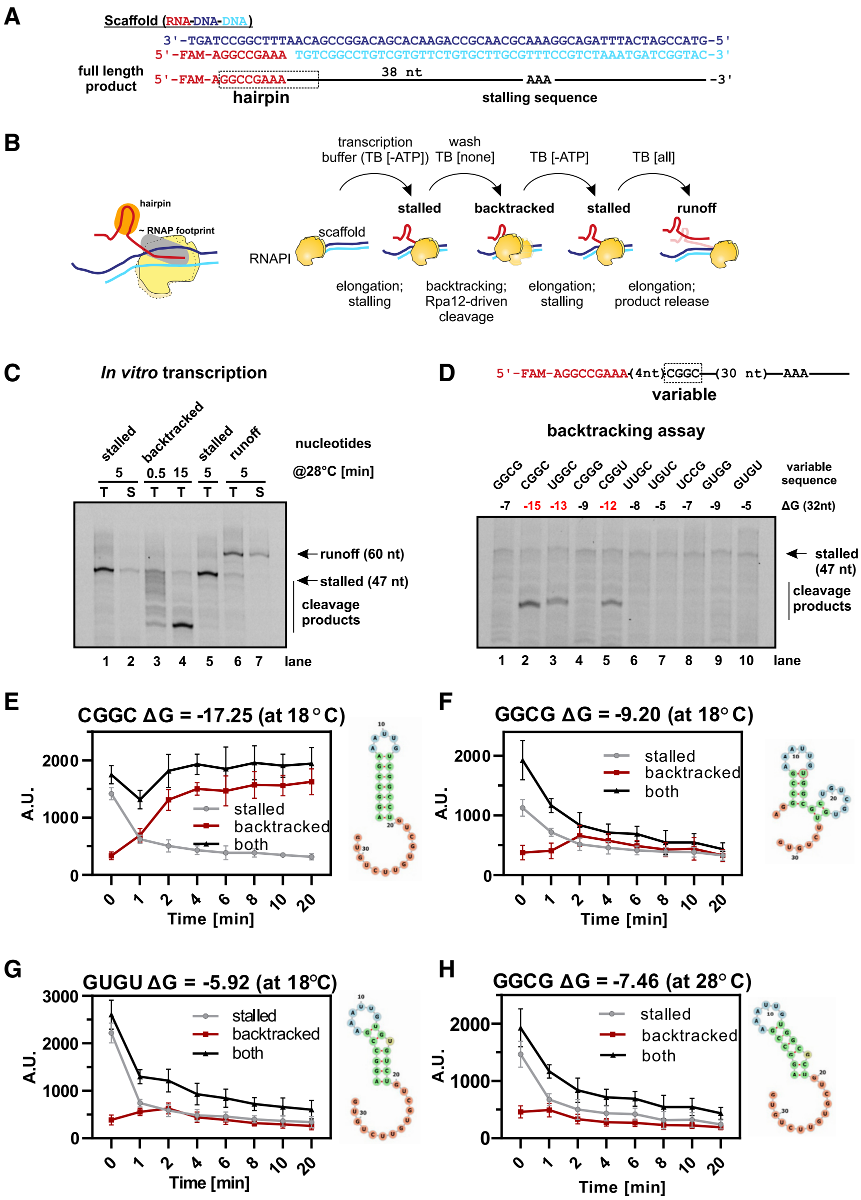

Figure 3. Strong Structures in the Nascent RNA Limit RNAPI Backtracking and Promote Elongation *In Vitro*

(A) Sequence of the RNA-DNA-DNA scaffold and schematic of the 60-nt RNA product with the stalling sequence marked (AAA).

(B) Schematic of the *in vitro* assay, showing the RNAPI elongation complex (yellow), DNA (light and dark blue), and RNA (red) with the hairpin structure highlighted (orange). [−ATP], transcription buffer (TB) without ATP to induce stalling (stalled); [none], incubation in TB without nucleotides to promote backtracking and Rpa12-driven cleavage (backtracked); [all], TB supplemented with all NTPs to generate runoff products (runoff).

(C) *In vitro* assay performed according to the scheme in (B). T, total; S, supernatant only. The experiment was performed in one tube, and aliquots were taken for each condition.

(D) *In vitro* assay of RNAPI backtracking. Top panel: schematic of the 60-nt RNA product with four variable nucleotides and stalling sequence (AAA). Bottom panel: set of 10 scaffolds with different folding energies of nascent RNA, transcribed in TB without ATP to induce stalling, washed, and incubated for 15 min at 28°C to allow Rpa12-driven cleavage. Only sequences with the highest stability limited RNAPI backtracking and produced prominent bands.

(E–H) Effects of structured RNA in reducing back-translocation *in vitro,* using four constructs with different folding energies. The assay was performed as in (D). Shown is quantification of stalled peaks, backtracked peaks, and the sum of both. Variable scaffold sequence, incubation temperature, and predicted folding energy are marked above each plot. Predictions of nascent, extruded RNA structure at the indicated temperature are shown on the right of each plot. Error bars present standard deviation (n = 3).

See also Figure S3.

Weaker structures did not generate stable stalls at 28°C but might still affect RNAPI back-translocation. To assess this, the RNAPI backtracking assay was analyzed at short time points and with reduced temperature (18°C) to slow the polymerase (Figures 3E–3G).

For the strongest hairpin, CGGC (ΔG −15 at 28°C and ΔG −17 at 18°C), we observed very rapid backtracking even at 18°C (Figures 3E and S3B). By 2 min, nearly all RNAPI complexes were lost from the stalled position and accumulated in backtracked positions stabilized by the 5′ terminal stem. These complexes were then stable for at least 20 min of incubation.

We next tested two hairpins that did not generate stable products at 28°C: GGCG (ΔG −7 at 28°C and ΔG −9 at 18°C) and GUGU (ΔG −5 at 28°C and ΔG −6 at 18°C). At 18°C, both transcripts generated a clear but transient gel band corresponding to backtracked RNAPI that was most prominent at 2 min and destabilized during longer incubation (Figures 3F, 3G, S3D, and S3E). This was more persistent for the more stable GGCG transcript than for GUGU. We also tested the GGCG transcript over a time course at 28°C (Figures 3H and S3F). The backtracked peak was reduced at 28°C but still observed after 10 min of incubation and produced an RNA shortened to 6 nt (Figure S3G).

Altogether, these kinetic assays revealed that strong structures block backtracking, whereas weaker structures slow the kinetics of back translocation proportional to their stability.

Mathematical Model of RNAPI Transcription

To better understand the contributions of the different components to overall transcription, we developed a mathematical model for RNAPI transcription. The model is based on simulations of individual RNAPI molecules initiating and transcribing a

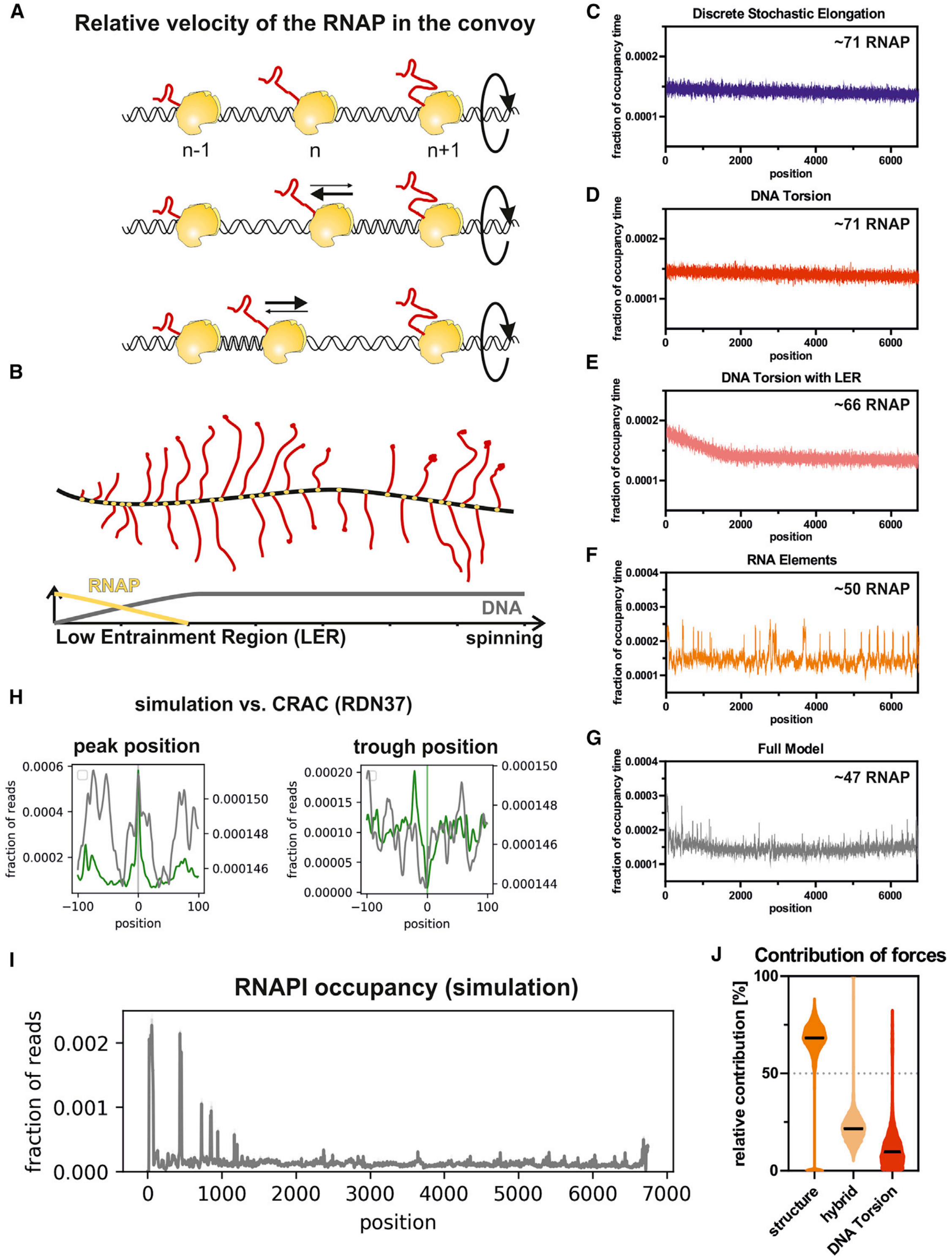

Figure 4. Mathematical Model of RNAPI Transcription

(A) Schematic of polymerase convoys in which a group of RNAP complexes moves along the rDNA transcription unit while DNA screws through the polymerases. In this model, the distance between initially loaded RNAP molecules is retained by the DNA helix, which behaves as an elastic rod and generates force when over- or under-wound.

(B) Schematic representation of the low entrainment region (LER). RNAPI molecules are initially able to rotate around the DNA, allowing changes in their relative positions without generating torsion. Grey line, degree of torsional entrainment; yellow line, ability of RNAPI to reduce torsion by rotation around the rDNA.

(legend continued on next page)

CellPress
OPEN ACCESS

7,000-nt RNA. The key parameters of the model include the (stochastic) initiation frequency and the probability of forward or reverse translocation. The latter is influenced by several factors: (1) the effects of DNA torsion on the probability of elongation versus backtracking, (2) the effects of structure in the nascent transcript, and (3) the stability of RNA-DNA duplex in the transcription bubble (Figures 4 and S4).

The parameters are very briefly described below and discussed in more detail in the STAR Methods. In this section, "RNAP" is used for statements universal to all RNAPs and "RNAPI" for features specific to RNAPI.

Starting Premises

Stochastic Initiation Events

Based on published data, we tested rates of stochastic initiation over a range of 0.33–1.0 s^{-1}, limited by the requirement that the preceding RNAPI has cleared the initiation region. A mean stochastic initiation rate of 0.8 s^{-1} generated RNAPI loading similar to that observed with Miller spreads (~50 per rDNA unit) (Figure S4I).

Stochastic Elongation

The reported average *in vivo* transcription rate across the entire yeast 35S pre-rRNA is ~40 nt s^{-1} (Kos and Tollervey, 2010), generated by the sum of multiple stochastic events. Although transcription elongation rates are often described as a velocity, the polymerase does not have momentum, and the time delay for each translocation event is independent and stochastic. At each time step in the model, the probability of translocation is random, chosen from a distribution derived from experimental data. The sum of these discrete stochastic delays creates the measured transcription rate. Together with stochastic initiation, this generated a model for the distribution of RNAP termed "stochastic elongation."

Effects of DNA Torsion

During transcription, the DNA or the RNAP plus the nascent transcript must rotate through 360° for each 10.5 nt incorporated (Figure 4A). If all RNAP molecules move in synchrony, then the torque from each will be equal, so no torsional stress will accumulate between adjacent polymerases. However, alterations in relative positions will result in positive supercoils between approaching polymerases and negative supercoils between separating polymerases (Figure 4A). The torque generated by torsion acts as an elastic rod, resulting in torsional entrainment of relative RNAP separation. The effect on elongation of this torque-assisted motion is included in the model as "DNA torsion." The effects of DNA torque were implemented progressively, from 0 at the initiation site, where the polymerase can rotate freely around the DNA, to 100% at +2 kb (Figure 4B). In the model, this is the "low entrainment region" (LER). In this region, neighboring RNAPI complexes can change relative positions without generating high torsional stress, potentially allowing more freedom to respond to effects of the nascent transcript.

Effects of the Nascent Transcript Sequence

Folding of the nascent transcript was incorporated with high stability (low ΔG; calculated using a 65-nt rolling window plus 15-nt offset) correlated with increased probability of rapid elongation and decreased probability of backtracking. The correlation between RNAP density and stability of the RNA:DNA duplex in the transcription bubble was incorporated with high stability (low ΔG; calculated using an 8-nt rolling window) correlated with decreased probability for rapid elongation. The effect of each feature was calculated for every nucleotide position. For ease of implementation, these were combined in the model as "RNA elements."

Modeling Indicates a Major Role of RNA Folding

We constructed a set of dynamic models that were run to achieve equilibrium states (Figures 4C–4G).

Discrete stochastic elongation alone generated a uniform distribution along the rDNA because each polymerase moves independently with a stochastic distribution of step times and variability generated by stochastic initiation (Figure 4C). A model implementing DNA torsion alone gives a broadly similar, relatively uniform profile (Figure 4D). All polymerases are constrained to move as a single convoy, with DNA torsion effects between polymerases accelerating and periodically stalling elongation. Neither of these models closely matches the *in vivo* electron microscopy (EM) and CRAC data. Inclusion of a 5′ low entrainment region generated a distribution that more closely matched the *in vivo* data because we now see a clear 5′ bias in modeled RNAPI density, with polymerases moving more slowly and more closely positioned over the initial 2 kb (Figure 4E). The model including only the RNA elements generated a highly uneven polymerase distribution, reflecting differences in folding energy and base composition across the entire rDNA (Figure 4F). Finally, incorporating all of these features into a single model gave a distribution closely approximating the *in vivo* data (Figures 4G and 4I). This shows the 5′ enrichment and relatively

(C) Modeled RNAPI occupancy along the transcription unit using a model of stochastic initiation and discrete, stochastic elongation. The average number of RNAP molecules per transcription unit is indicated. The average RNAPI occupancy for 64 simulations is presented. Each simulation was run for 2,000 sec and 200 time points were collected (C–G).
(D) Modeled RNAPI occupancy for the DNA torsion model.
(E) Modeled RNAPI occupancy for the DNA torsion model, including a LER of 2,000 nt. DNA torsion is engaged linearly between positions 0 and 2,000.
(F) RNAPI occupancy for the model with RNA elements. Translocation is modified positively by structure in the nascent RNA extruded from the polymerase and negatively by the stability of the RNA:DNA hybrid within the transcription bubble.
(G) Full model including DNA torsion, the LER, and RNA elements. This recapitulates experimental data to the greatest extent. Areas that do not correlate with experimental data are predicted to be affected by *trans*-acting factors binding co-transcriptionally (Discussion).
(H) RNAPI CRAC peak and trough metaplots together with simulated data. Shown is a full model (gray) relative to CRAC data (green).
(I) Modeled RNAPI occupancy plot generated using the full model. Four datasets of 256 simulations were used to generate profiles. *In silico* "reads" were sampled from interpolated profiles, generated as in G, and processed in the same way as RNAPI CRAC data.
(J) Violin plot of factor contributions at each elongation step within the full model.
See also Figure S4.

discrete peaks observed in the EM and CRAC data. As a potential source of the 5′ bias, we also considered premature termination of transcription. However, 30% reduced RNAP numbers per gene were needed to match the observed 5′ bias (Figures S4G and S4H), and this was excluded as a key factor in the model.

Alignments of peak and trough locations from the model with the experimentally derived peaks and troughs showed a clear overlap (Figures 4H; modeled data in gray, CRAC data in green). This confirmed that the model significantly recapitulates the experimental data at high resolution. Major discrepancies are speculated to reflect sites where backtracking is limited by stable binding of *trans*-acting factors rather than stem structures (Discussion).

In the final model, the relative contribution of forces from different elements is clearly dominated by RNA folding (Figure 4J), whereas DNA torsion has the weakest effect at each elongation step. However, entrainment alters the elongation kinetics in the same direction over multiple steps to maintain relative RNAPI positions.

A striking conclusion from the model concerns the combined effects of the different features on the probability of RNAPI backtracking and collisions (Figure 5). Stochastic elongation alone generates a low frequency of backtracking but a high frequency of collisions (Figures 5A and 5B). Inclusion of torque, generated from DNA torsion, reverses this: increased probability of backtracking and reduced probability of collisions. Backtracking and collisions are substantially suppressed by also including RNA structure (RNA elements). The final model suggests that RNAPI takes advantage of a low frequency of backtracking because of RNA structure and a low level of collisions because of DNA torsion.

The presence of a strongly folded 5′ ETS region in the pre-rRNA is conserved among eukaryotes. However, the primary sequence and length of the 5′ ETS are variable between species. We therefore assessed how overall folding of the 5′ ETS affects transcriptional output by modeling a set of alternative structures (Figure 5C) with (1) decreased ΔG over the 5′ ETS region or (2) altered spacing between the hairpins (Figures 5C–5F and S5A–S5C). For this analysis, the effect of the transcription bubble sequence was disregarded.

Consistent with the results in Figure 3, the decreased folding energy of the 5′ ETS region caused increased RNAP backtracking (Figure 5D), whereas collisions (Figure 5E) and the total number of RNAP particles (Figure S5B) remained unaffected. The fraction of backtracked RNAP correlated with the average ΔG over the 5′ ETS (Figure 5F). Surprisingly, modification of spacing between the 5′ ETS hairpins (see overview in Figure S5C) did not strongly affect output from the simulation (Figures S5D–S5F). Together, these results indicate that strong secondary structures in the 5′ ETS are functionally important in reducing RNAP backtracking.

The 5′ proximal hairpin in the 5′ ETS is distinct. Analysis of structures and folding energy reveals very weak intermediate structures in comparison with the full-length hairpin for *S. cerevisiae* (Figure S5G), humans (Figure S5H), and other characterized species (*S. pombe* and *M. musculus*). The RNAPI footprint was estimated at 38 bp by cryo-EM, so strong structures within this region will potentially accelerate promoter clearance and increase rDNA loading. We analyzed a set of *in silico* constructs with the 5′ ETS fixed to $\Delta G = -20$ kcal mol^{-1} starting at positions 0 nt, 10 nt, etc., up to 80 nt. The 5′ ETS with structures starting at early positions (0–30 nt) indeed increased effective initiation and rDNA loading (Figure 5I), but this was associated with increased backtracking and collisions (Figures 5G and 5H). We speculate that this lack of short stable 5′ structures reduces overloading of the rDNA transcription unit.

Effects of RNA Folding Are Widespread and Have Regulatory Potential

The key conclusions derived for RNAPI are expected to hold for all other polymerases and species. We therefore assessed the effects of nascent transcript structure for *Schizosaccharomyces pombe* RNAPI and other RNAPs in budding yeast.

The *S. pombe* RNAPI CRAC profile revealed an uneven distribution with a 5′ bias (Figure 6A), similar to *S. cerevisiae* RNAPI (Figure 1E). Metaplot analysis of troughs in RNAPI density versus folding energy of the nascent transcript revealed a strong correlation (Figure 6B; $p = 3 \times 10^{-4}$).

RNAPIII generally transcribes very short pre-tRNA transcripts. However, the RNAPIII-transcribed *SCR1* gene encodes the 522-nt-long scR1 ncRNA component of the signal recognition particle. Previous RNAPIII CRAC data showed a very uneven distribution across *SCR1* (Turowski et al., 2016). A peak and trough metaplot for RNAPIII density versus the folding energy of the nascent scR1 RNA revealed a high degree of correlation (Figures 6C, 6D, and S6A), similar to that observed for RNAPI; however, the number of features was too low to perform statistical analysis.

Published high-resolution analyses of RNAPII distribution by NET-seq or CRAC, using the catalytic subunit Rbp1, also reveal strikingly uneven density (Churchman and Weissman, 2011; Milligan et al., 2016; Figure S6B). Independent biological replicates for Rpb1 distribution in NET-seq and CRAC showed good reproducibility across well-transcribed genes (Figure S6C), indicating that the fluctuations represent genuine differences in RNAPII density.

Some of the variation in RNAPII occupancy reflects nucleosome positioning, with maximal density (minimal RNAPII elongation rate) seen at the center of nucleosomes (Churchman and Weissman, 2011; Milligan et al., 2016), which are generally well positioned in yeast. To determine whether structure in nascent transcripts also affects RNAPII occupancy, we used a peak-calling algorithm to define peaks and troughs in the RNAPII density across 50% of the most highly transcribed genes that are longer than 300 nt (n = 1,073). We used NET-seq peaks to generate metaplots because published RNAPII CRAC data were prepared using a protocol that does not specifically recover the nascent 3′ end. This showed a correlation between the RNAPII peaks (n = 9,927) and troughs (n = 4,776) and the rolling average of predicted ΔG (shown for a 65-nt window with a 15-nt offset in Figures 6E and 6F). RNAPII occupancy peaks were associated with a clear peak of folding energy, whereas troughs, indicating rapid elongation, were associated with stronger nascent RNA structure (Figures 6E, $p = 5 \times 10^{-15}$, and 6F, $p = 5 \times 10^{-8}$, Wilcoxon signed-rank test, orange line).

To determine whether nascent RNA structure may have regulatory potential, we used pre-mRNA splicing as a model process.

CellPress
OPEN ACCESS

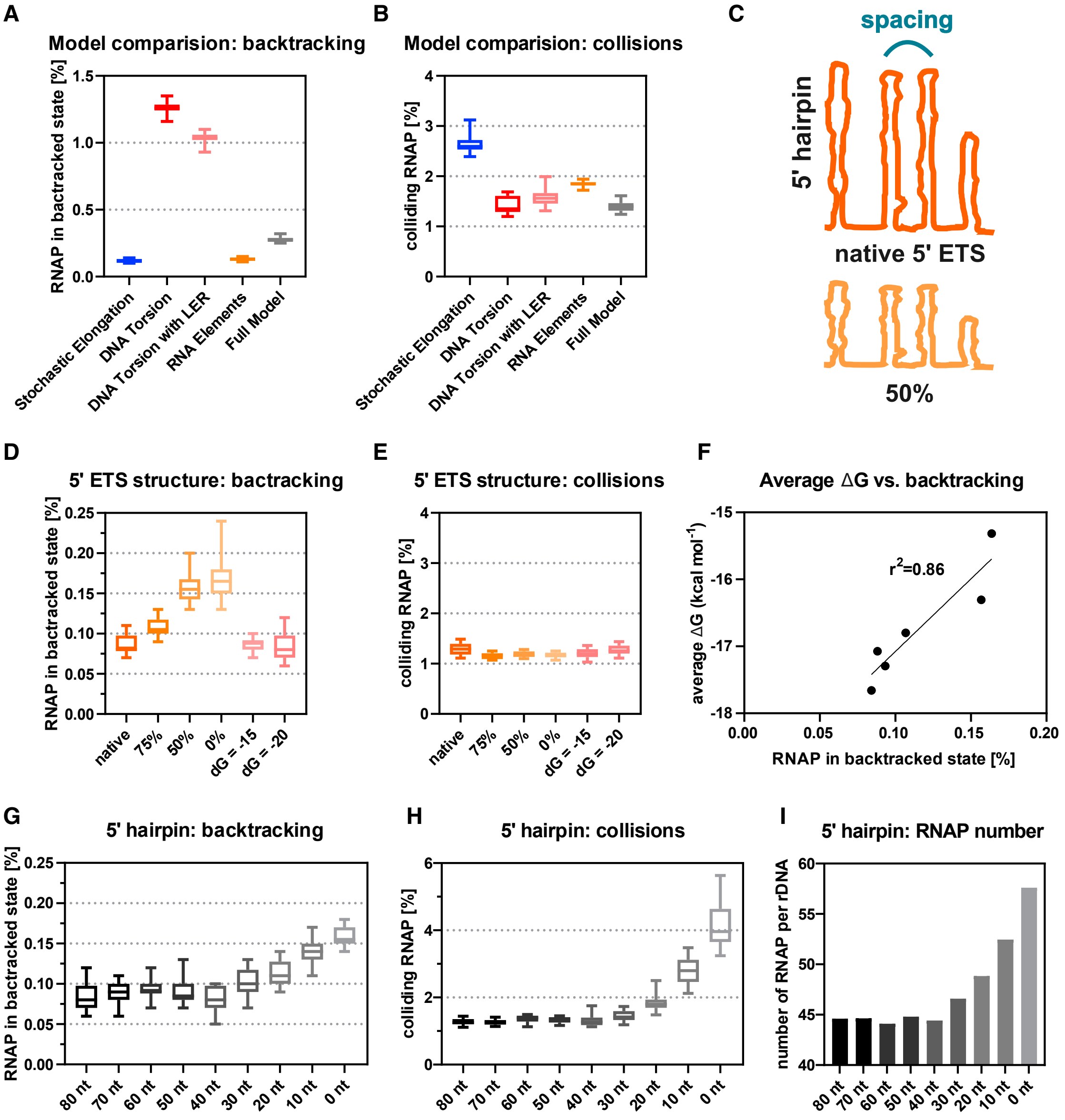

Figure 5. Conclusions from the Model of RNAPI Transcription

(A) RNAP in backtracked state. Backtracking events were saved for each of the simulations (Figures 4C–4G). Percentages of RNAP found in the backtracked state in different models were calculated and are presented as a boxplot.

(B) Frequency of colliding RNAP, calculated as in (A).

(C) Overview of the 5′ ETS structure modifications: changing spacing between hairpins (top panel) or reducing strength of RNA structures (bottom panel).

(D) Frequency of RNAP backtracking in response to weaker 5′ ETS structures. The 5′ ETS with RNA structures reduced to 75%, 50%, or 0% or fixed to $\Delta G = -15/-20$ kcal mol^{-1} was used.

(E) Frequency of colliding RNAP in response to weaker 5′ ETS structures.

(F) Frequency of RNAP in the backtracked state is correlated with average stability of RNA structures. ΔG was calculated for the entire rDNA with a modified 5′ ETS.

(G) Frequency of RNAP in backtracking in response to altered 5′ hairpin positioning within the 5′ ETS. The 5′ ETS with fixed $\Delta G = -20$ kcal mol^{-1} was used to test the effect of changes in 5′ structures.

(H) Frequency of colliding RNAP in response to the altered 5′ hairpin positioning.

(I) Number of RNAPs per rDNA in response to the altered 5′ hairpin positioning.

See also Figure S5.

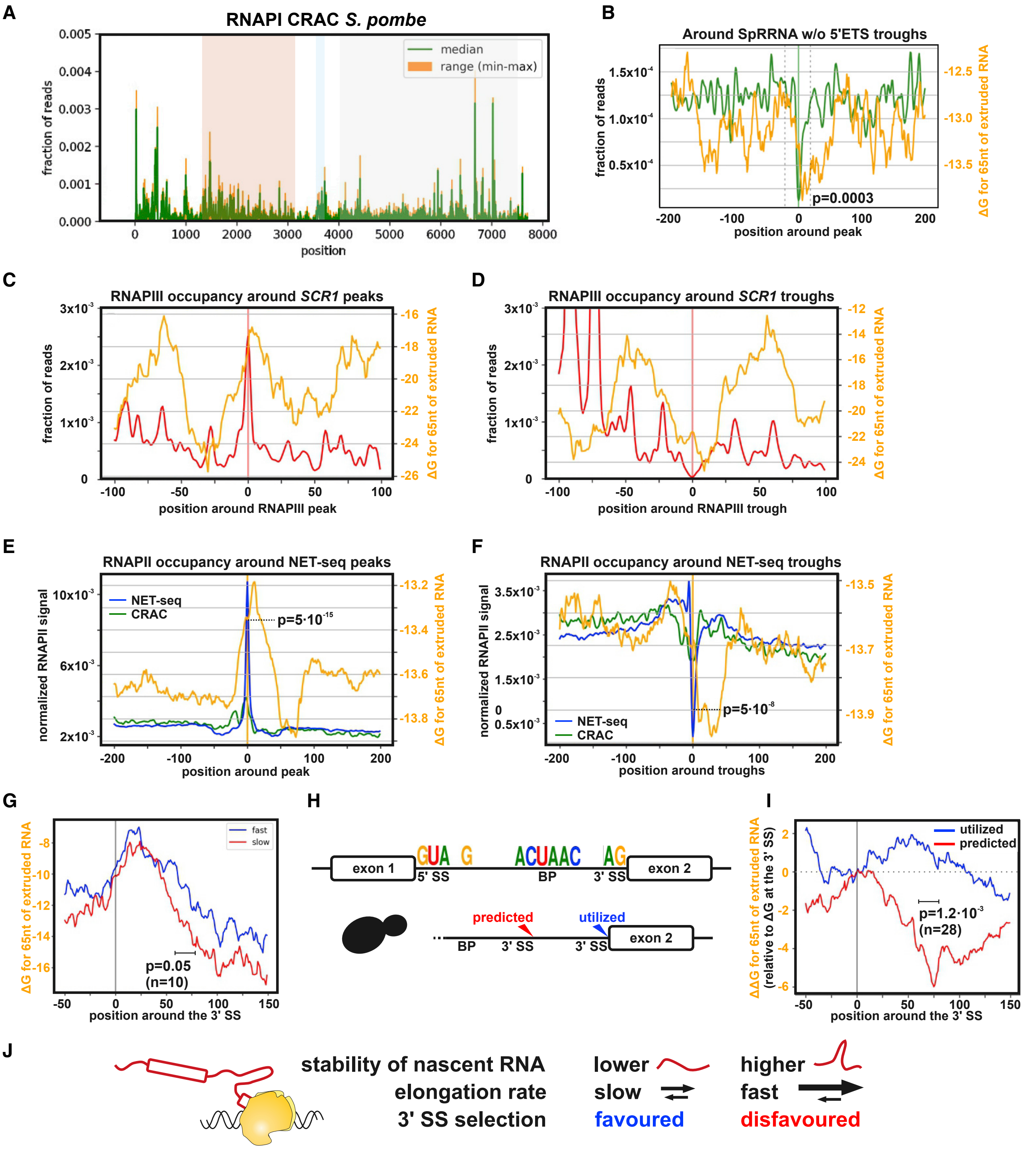

Figure 6. Effects of Transcript Folding Are Widespread and Have Regulatory Potential

(A) *S. pombe* RNAPI CRAC distribution over the rDNA gene encoding the pre-rRNA. Annotation are as in Figure 1E (n = 2).

(B) *S. pombe* RNAPI CRAC trough metaplot for the entire pre-rRNA with folding energy of the nascent transcript. The p value was calculated using a Wilcoxon rank-sum test for a 400-nt window versus a 40-nt central window (n = 80).

(C) RNAPIII CRAC peak metaplot compared with predicted folding of nascent scR1 ncRNA. Folding energy (ΔG in kilocalories per mole) was calculated for a rolling 65-nt window behind RNAPIII, offset by 15 nt (GEO: GSE77863).

(D) As (C), except that CRAC troughs were compared with folding of nascent scR1.

(legend continued on next page)

The splicing machinery co-transcriptionally recognizes the 5′ splice site (SS), branchpoint (BP), and the 3′ SS (acceptor site). We predicted that stronger structure in the nascent RNA would reduce the time available for co-transcriptional selection of the 3′ SS, disfavoring rapid cotranscriptional splicing. Analyses using extremely fast metabolic labeling previously ranked yeast pre-mRNAs by splicing speed (Barrass et al., 2015; Figure S6D). Consistent with our hypothesis, the fastest third of spliced genes had less structure in the nascent RNA at the start of exon 2 compared with the slowest third of spliced genes (Figure 6G, $p = 0.05$, Wilcoxon rank-sum test for $n = 10$).

The 3′ SS consensus is notably weak, consisting of only two bases (AG), suggesting a kinetic model for 3′ SS selection based on a "window of opportunity." To assess the potential role of nascent RNA folding as a decisive factor, we defined all yeast introns *de novo* using previously described features (Figure 6H), focusing on those with a predicted but unutilized 3′ SS upstream of the authentic site. Then we compared changes in folding energy of the nascent RNA downstream of the predicted and utilized 3′ SS (Figure 6I for ΔΔG, $p = 1.2 \times 10^{-3}$, Wilcoxon rank-sum test and Figure S6E for ΔG, $p > 0.05$). Nascent RNA extruded after transcription of the utilized 3′ SS maintained RNA folding (ΔΔG) on similar level, whereas predicted but unutilized 3′ SS RNAs are accompanied by stronger folding of nascent RNA (ΔΔG). Interestingly, this would suggest that relative folding energy (ΔΔG) is more important for selection of the 3′ SS because the overall stability of nascent RNA was not significantly different (Figure S6E; $p > 0.05$). Stronger folding of the nascent RNA may accelerate RNAPII and decrease the window of opportunity for splicing to occur, potentially favoring skipping of the unused, potential 3′SS (Figure 6J). Notably, significant differences were seen for folding energy of the nascent RNA, even when the folding window did not include the 3′SS, making it unlikely that direct effects on the structure or accessibility of the acceptor site are responsible for the observed correlations.

We conclude that stimulation of transcription elongation by nascent RNA structure is a conserved feature of all three eukaryotic polymerases and that regulation of co-transcriptional processes is at least partially determined by local folding of nascent RNA.

DISCUSSION

Analyses of eukaryotic transcription by multiple techniques reveal uneven polymerase occupancy, reflecting variable elongation rates. This is important because many RNA processing factors act very quickly on the nascent transcript. For example, splicing of pre-mRNA is strikingly speedy in yeast (Wallace and Beggs, 2017) but more heterogeneous in metazoans (Alpert et al., 2017; Drexler et al., 2020), potentially altering alternative splicing (Saldi et al., 2016). Understanding the detailed kinetics of transcription elongation *in vivo* will therefore be predictive of processing decisions.

In eukaryotes, RNAPI is most amenable to these analyses because it transcribes only a single product from the nucleosome-free rDNA. EM analyses of Miller chromatin spreads revealed uneven distribution of RNAPI across the rDNA, with an excess of polymerases in the 5′ region. In an orthogonal approach, we determined the distribution of RNAPI by CRAC UV crosslinking. This confirmed the 5′ enrichment for RNAPI density but also revealed a strikingly uneven, local polymerase distribution, most notably over the 5′ ETS region of the pre-rRNA (Figures 1E and 1F).

Analysis of features that correlate with peaks and troughs of RNAPI density showed a modest correlation with the stability of the RNA-DNA duplex in the transcription bubble but strong correlation with the calculated folding energy of the nascent pre-rRNA transcript close to the polymerase (Figure 2). RNAPs operate as Brownian ratchets and are prone to backtracking, which serves as a proofreading step (Figure 1A). During backtracking, the newly transcribed RNA must re-enter the polymerase. However, the transcription bubble region of RNAPI is only large enough for single-stranded RNA (Tafur et al., 2016). Backtracking is therefore resisted by any RNA structures that form sufficiently rapidly in the nascent transcript, as proposed previously for bacterial RNAP (Dangkulwanich et al., 2014).

Using RNAPI transcription *in vitro*, we confirmed that strong structures in the nascent transcript effectively resist backtracking and defined the stability of stems that can block or slow backtracking by RNAPI (Figure 3). Additionally, our genome-wide data provide evidence that RNA structure substantially modulates transcription elongation by RNAPII and RNAPIII (Figure 6).

Any *trans*-acting factors that rapidly and stably bind the nascent RNA are also predicted to resist backtracking. Supporting this conjecture, we note that there were fewer discrete 5′ ETS peaks in the model than in the CRAC data. The prominent CRAC peak around +100 corresponds with the major binding site for the UTP-A complex of early-binding ribosome synthesis factors

(E) Metaplot showing peaks of NET-seq density for RNAPII (GEO: GSE25107). Peaks from the top 50% of RNAPII transcripts longer than 300 nt were overlaid with the folding energy of the nascent transcripts. Folding energy (ΔG in kilocalories per mole) was calculated for a rolling 65-nt window behind RNAPII, offset by 15 nt. Each position on the x axis shows the average folding energy for the nascent transcripts associated with all polymerases located at that distance from the peak. The p value was calculated using a Wilcoxon signed-rank test ($n = 9{,}844$).
(F) As (E) but showing troughs in NET-seq density ($n = 4{,}749$).
(G) Folding of nascent RNA around the 3′ SS of pre-mRNA genes classified as fastest third and slowest third for spliced, non-ribosomal protein genes (Barrass et al., 2015). The p value was calculated using a Wilcoxon rank-sum test ($n = 10$).
(H) Conserved feature of yeast introns used for *de novo* prediction (top panel). Genes containing a predicted but skipped 3′ SS were analyzed ($n = 28$, bottom panel).
(I) Relative folding energy of nascent RNA around the utilized 3′ SS versus the predicted but skipped 3′ SS relative to the 3′ SS (position 0). The p value was calculated using a Wilcoxon rank-sum test ($n = 28$).
(J) Suggested role of nascent RNA stability in selection of the 3′ SS.
See also Figure S6.

(Hunziker et al., 2016; Sun et al., 2017), which have been implicated previously in transcription and were designated t-Utps (Gallagher et al., 2004). Similarly, a CRAC peak further 3′ is close to the major U3 small nucleolar RNA (snoRNA) binding site at +470. We postulate that RNA packaging factors bound to nascent transcripts also function as ratchets, favoring progressive RNAPI elongation.

To better understand the contributions of different features to the behavior of RNAPI *in vivo*, we developed a mathematical model of rDNA transcription. Notably, the model revealed that inclusion of the effects of torque reduced the numbers of colliding RNAPI but increased the fraction of RNAPI in a backtracked position (Figures 5A and 5B). Addition of nascent transcript folding reduced the frequency of backtracking while retaining the low level of collisions. This underlined the positive contribution of RNA structure to productive elongation.

The yeast 5′ ETS is notably highly structured, which may partly reflect selection of structures that promote efficient transcription. Structures within the 5′ ETS decreased the frequency of RNAPI backtracking, and this effect correlated with the overall ΔG of the nascent RNAs. Notably, relatively sharp peaks of RNAPI density correlated with the apexes of the extended stem structures in the ETS. We speculate that this arises because the lowest enhancement of elongation resulting from RNA structure occurs at these sites. Weaker, transient structures will have formed during extrusion of the 5′ sides of the extended stems, giving some boost to elongation, but these must be unfolded prior to refolding into the extended final stems.

Our key findings regarding the effects of folding in the nascent transcript on polymerase elongation may also be applicable to RNAPI from *S. pombe* and RNAPII and RNAPIII from *S. cerevisiae* and potentially RNAPs in many or all other systems. Although folding energy of the nascent transcript emerged as a the most significant feature in determining RNAPI elongation rates, its role in RNAPII elongation is expected to be tempered by many other factors affecting elongation (Gressel et al., 2019). Despite this, an apparent correlation between RNA folding and polymerase density was clearly seen by CRAC crosslinking and in NET-seq data, which use orthogonal approaches.

Signals within pre-mRNAs defining SSs have surprisingly little information content relative to splicing fidelity, and multiple additional features contribute to accurate SS selection. We propose that unstructured RNA downstream of the intron favors slowed elongation of RNAPII, which facilitates splicing by allowing more time for recognition of the 3′ SS by splicing factors associated with C-terminal domain of the polymerase. In contrast, structured RNA may promote rapid elongation, favoring distal SS use. Notably, the window of opportunity for 3′ SS recognition is presumably substantially shorter than the actual pre-mRNA splicing reaction, as assessed by transcript sequencing (Alpert et al., 2017; Drexler et al., 2020; Neugebauer, 2019; Wachutka et al., 2019; Wallace and Beggs, 2017). Finally, we note that similar considerations potentially apply to other cotranscriptional events that depend on RNAPII-associated recognition, including alternative polyadenylation.

STAR★METHODS

Detailed methods are provided in the online version of this paper and include the following:

- KEY RESOURCES TABLE
- RESCOURCE AVAILABILITY
  - Lead Contact
  - Materials Availability
  - Data and Code Availability
- EXPERIMENTAL MODEL AND SUBJECT DETAILS
  - Strains
- METHOD DETAILS
  - Miller Chromatin Spreads and Measurements of Polymerase Positions
  - In-vivo RNA crosslinking
  - CRAC
  - Purification of RNA polymerase I and *in vitro* assay
  - Validation of RNAPI CRAC data
  - Development of Mathematical model for RNAPI transcription
  - 1. Quantification of molecules of RNA polymerases
  - 2. Transcription initiation rate
  - 3. RNAPI number per rDNA transcription unit and RNAPI spacing
  - 4. Elongation rate of RNAP in the discrete model
  - 5. RNAP convoys imply DNA torsion effects
  - 6. Range of DNA stiffness constant *c*
  - 7. Low Entrainment Region
  - 8. Role of nascent RNA in transcription elongation
  - 9. Optional elements of the model
  - Numerical convergence of the model
  - Model optimization
  - Data sampling
  - Relative contribution of model elements
- QUANTIFICATION AND STATISTICAL ANALYSIS
  - Pre-processing and data alignment
  - RNA polymerase I profile
  - Profile analysis: peak/trough calling and metaplots
  - CRAC versus PAR-CRAC comparison
  - Analysis of G+C-richness
  - Folding of nascent RNA
  - Analysis of splicing
  - Statistical analyses

SUPPLEMENTAL INFORMATION

Supplemental Information can be found online at https://doi.org/10.1016/j.molcel.2020.06.002.

ACKNOWLEDGMENTS

We thank Grzegorz Kudla for critical reading of the manuscript; the members of Tollervey group and Guido Sanguinetti for stimulating discussions; Christoph Engel, Joachim Griesenbeck, and Michael Pilsl for assistance with the *in vitro* assay; and Tomas Gedeon, Lisa David, and Tamra Heberling for advice regarding the mathematical model. D.T. was supported by Wellcome (077248). T.W.T. was supported by the Polish Ministry of Science and Higher Education Mobility Plus program (1069/MOB/2013/0). S.L.F. was supported

CellPress
OPEN ACCESS

by the NIH (GM06952). Work in the Wellcome Centre for Cell Biology is supported by a Centre Core grant (203149).

AUTHOR CONTRIBUTIONS

T.W.T. and D.T. conceived the project and wrote the manuscript. T.W.T., E.P., and S.L.F. performed experiments. T.W.T., A.H., and D.T. analyzed the data. B.D.G., T.W.T., and D.T. developed the mathematical model. All authors edited and reviewed the manuscript.

DECLARATION OF INTERESTS

The authors declare no competing interests.

Received: March 2, 2020
Revised: May 1, 2020
Accepted: May 28, 2020
Published: June 24, 2020

SUPPORTING CITATIONS

The following references appear in the Supplemental Information: Cortazar et al. (2019); Dasgupta et al. (2007); French et al. (2003); Fuchs et al. (2014); Gressel et al. (2017); Hontz et al. (2008); Jonkers et al. (2014); Nomura (1999); Oakes et al. (2006); Sandmeier et al. (2002); Schneider et al. (2006); Tongaonkar et al. (2005); Toussaint et al. (2005); Viktorovskaya and Schneider, 2015.

REFERENCES

Adelman, K., La Porta, A., Santangelo, T.J., Lis, J.T., Roberts, J.W., and Wang, M.D. (2002). Single molecule analysis of RNA polymerase elongation reveals uniform kinetic behavior. Proc. Natl. Acad. Sci. USA *99*, 13538–13543.

Alpert, T., Herzel, L., and Neugebauer, K.M. (2017). Perfect timing: splicing and transcription rates in living cells. Wiley Interdiscip. Rev. RNA *8*, https://doi.org/10.1002/wrna.1401.

Axt, K., French, S.L., Beyer, A.L., and Tollervey, D. (2014). Kinetic analysis demonstrates a requirement for the Rat1 exonuclease in cotranscriptional pre-rRNA cleavage. PLoS ONE *9*, e85703.

Bailey, T.L. (2011). DREME: motif discovery in transcription factor ChIP-seq data. Bioinformatics *27*, 1653–1659.

Barrass, J.D., Reid, J.E.A., Huang, Y., Hector, R.D., Sanguinetti, G., Beggs, J.D., and Granneman, S. (2015). Transcriptome-wide RNA processing kinetics revealed using extremely short 4tU labeling. Genome Biol. *16*, 282.

Booth, G.T., Wang, I.X., Cheung, V.G., and Lis, J.T. (2016). Divergence of a conserved elongation factor and transcription regulation in budding and fission yeast. Genome Res. *26*, 799–811.

Bormuth, V., Varga, V., Howard, J., and Schäffer, E. (2009). Protein friction limits diffusive and directed movements of kinesin motors on microtubules. Science *325*, 870–873.

Brill, S.J., DiNardo, S., Voelkel-Meiman, K., and Sternglanz, R. (1987). Need for DNA topoisomerase activity as a swivel for DNA replication for transcription of ribosomal RNA. Nature *326*, 414–416.

Bustamante, C., Chemla, Y.R., Forde, N.R., and Izhaky, D. (2004). Mechanical processes in biochemistry. Annu. Rev. Biochem. *73*, 705–748.

Chong, Y.T., Koh, J.L., Friesen, H., Duffy, S.K., Cox, M.J., Moses, A., Moffat, J., Boone, C., and Andrews, B.J. (2015). Yeast Proteome Dynamics from Single Cell Imaging and Automated Analysis. Cell *161*, 1413–1424.

Churchman, L.S., and Weissman, J.S. (2011). Nascent transcript sequencing visualizes transcription at nucleotide resolution. Nature *469*, 368–373.

Cioci, F., Vu, L., Eliason, K., Oakes, M., Siddiqi, I.N., and Nomura, M. (2003). Silencing in yeast rDNA chromatin: reciprocal relationship in gene expression between RNA polymerase I and II. Mol. Cell *12*, 135–145.

Clarke, A.M., Engel, K.L., Giles, K.E., Petit, C.M., and Schneider, D.A. (2018). NETSeq reveals heterogeneous nucleotide incorporation by RNA polymerase I. Proc. Natl. Acad. Sci. USA *115*, E11633–E11641.

Cortazar, M.A., Sheridan, R.M., Erickson, B., Fong, N., Glover-Cutter, K., Brannan, K., and Bentley, D.L. (2019). Control of RNA Pol II Speed by PNUTS-PP1 and Spt5 Dephosphorylation Facilitates Termination by a “Sitting Duck Torpedo” Mechanism. Mol. Cell *76*, 896–908.e4.

Dammann, R., Lucchini, R., Koller, T., and Sogo, J.M. (1993). Chromatin structures and transcription of rDNA in yeast Saccharomyces cerevisiae. Nucleic Acids Res. *21*, 2331–2338.

Dangkulwanich, M., Ishibashi, T., Liu, S., Kireeva, M.L., Lubkowska, L., Kashlev, M., and Bustamante, C.J. (2013). Complete dissection of transcription elongation reveals slow translocation of RNA polymerase II in a linear ratchet mechanism. eLife *2*, e00971.

Dangkulwanich, M., Ishibashi, T., Bintu, L., and Bustamante, C. (2014). Molecular mechanisms of transcription through single-molecule experiments. Chem. Rev. *114*, 3203–3223.

Dasgupta, A., Sprouse, R.O., French, S., Aprikian, P., Hontz, R., Juedes, S.A., Smith, J.S., Beyer, A.L., and Auble, D.T. (2007). Regulation of rRNA synthesis by TATA-binding protein-associated factor Mot1. Mol. Cell. Biol. *27*, 2886–2896.

Dodt, M., Roehr, J.T., Ahmed, R., and Dieterich, C. (2012). FLEXBAR-Flexible Barcode and Adapter Processing for Next-Generation Sequencing Platforms. Biology (Basel) *1*, 895–905.

Drexler, H.L., Choquet, K., and Churchman, L.S. (2020). Splicing Kinetics and Coordination Revealed by Direct Nascent RNA Sequencing through Nanopores. Mol. Cell *77*, 985–998.e8.

El Hage, A., French, S.L., Beyer, A.L., and Tollervey, D. (2010). Loss of Topoisomerase I leads to R-loop-mediated transcriptional blocks during ribosomal RNA synthesis. Genes Dev. *24*, 1546–1558.

El Hage, A., Webb, S., Kerr, A., and Tollervey, D. (2014). Genome-wide distribution of RNA-DNA hybrids identifies RNase H targets in tRNA genes, retrotransposons and mitochondria. PLoS Genet. *10*, e1004716.

Engel, C., Sainsbury, S., Cheung, A.C., Kostrewa, D., and Cramer, P. (2013). RNA polymerase I structure and transcription regulation. Nature *502*, 650–655.

Engel, C., Gubbey, T., Neyer, S., Sainsbury, S., Oberthuer, C., Baejen, C., Bernecky, C., and Cramer, P. (2017). Structural Basis of RNA Polymerase I Transcription Initiation. Cell *169*, 120–131.e22.

French, S.L., Osheim, Y.N., Cioci, F., Nomura, M., and Beyer, A.L. (2003). In exponentially growing Saccharomyces cerevisiae cells, rRNA synthesis is determined by the summed RNA polymerase I loading rate rather than by the number of active genes. Mol. Cell. Biol. *23*, 1558–1568.

French, S.L., Osheim, Y.N., Schneider, D.A., Sikes, M.L., Fernandez, C.F., Copela, L.A., Misra, V.A., Nomura, M., Wolin, S.L., and Beyer, A.L. (2008). Visual analysis of the yeast 5S rRNA gene transcriptome: regulation and role of La protein. Mol. Cell. Biol. *28*, 4576–4587.

Fuchs, G., Voichek, Y., Benjamin, S., Gilad, S., Amit, I., and Oren, M. (2014). 4sUDRB-seq: measuring genomewide transcriptional elongation rates and initiation frequencies within cells. Genome Biol. *15*, R69, R69.

Gallagher, J.E., Dunbar, D.A., Granneman, S., Mitchell, B.M., Osheim, Y., Beyer, A.L., and Baserga, S.J. (2004). RNA polymerase I transcription and pre-rRNA processing are linked by specific SSU processome components. Genes Dev. *18*, 2506–2517.

Ghaemmaghami, S., Huh, W.K., Bower, K., Howson, R.W., Belle, A., Dephoure, N., O’Shea, E.K., and Weissman, J.S. (2003). Global analysis of protein expression in yeast. Nature *425*, 737–741.

Granneman, S., Petfalski, E., and Tollervey, D. (2011). A cluster of ribosome synthesis factors regulate pre-rRNA folding and 5.8S rRNA maturation by the Rat1 exonuclease. EMBO J. *30*, 4006–4019.

Gressel, S., Schwalb, B., Decker, T.M., Qin, W., Leonhardt, H., Eick, D., and Cramer, P. (2017). CDK9-dependent RNA polymerase II pausing controls transcription initiation. eLife *6*, e29736.

Gressel, S., Schwalb, B., and Cramer, P. (2019). The pause-initiation limit restricts transcription activation in human cells. Nat. Commun. *10*, 3603.

Guajardo, R., and Sousa, R. (1997). A model for the mechanism of polymerase translocation. J. Mol. Biol. *265*, 8–19.

Hall, D.B., Wade, J.T., and Struhl, K. (2006). An HMG protein, Hmo1, associates with promoters of many ribosomal protein genes and throughout the rRNA gene locus in Saccharomyces cerevisiae. Mol. Cell. Biol. *26*, 3672–3679.

Harada, Y., Ohara, O., Takatsuki, A., Itoh, H., Shimamoto, N., and Kinosita, K., Jr. (2001). Direct observation of DNA rotation during transcription by Escherichia coli RNA polymerase. Nature *409*, 113–115.

Heberling, T., Davis, L., Gedeon, J., Morgan, C., and Gedeon, T. (2016). A Mechanistic Model for Cooperative Behavior of Co-transcribing RNA Polymerases. PLoS Comput. Biol. *12*, e1005069.

Hontz, R.D., French, S.L., Oakes, M.L., Tongaonkar, P., Nomura, M., Beyer, A.L., and Smith, J.S. (2008). Transcription of multiple yeast ribosomal DNA genes requires targeting of UAF to the promoter by Uaf30. Mol. Cell. Biol. *28*, 6709–6719.

Hunziker, M., Barandun, J., Petfalski, E., Tan, D., Delan-Forino, C., Molloy, K.R., Kim, K.H., Dunn-Davies, H., Shi, Y., Chaker-Margot, M., et al. (2016). UtpA and UtpB chaperone nascent pre-ribosomal RNA and U3 snoRNA to initiate eukaryotic ribosome assembly. Nat. Commun. *7*, 12090.

Iborra, F.J., Pombo, A., McManus, J., Jackson, D.A., and Cook, P.R. (1996). The topology of transcription by immobilized polymerases. Exp. Cell Res. *229*, 167–173.

Jonkers, I., Kwak, H., and Lis, J.T. (2014). Genome-wide dynamics of Pol II elongation and its interplay with promoter proximal pausing, chromatin, and exons. eLife *3*, e02407.

Kim, S., Beltran, B., Irnov, I., and Jacobs-Wagner, C. (2019). Long-Distance Cooperative and Antagonistic RNA Polymerase Dynamics via DNA Supercoiling. Cell *179*, 106–119.e16.

Kos, M., and Tollervey, D. (2010). Yeast pre-rRNA processing and modification occur cotranscriptionally. Mol. Cell *37*, 809–820.

Kuhn, C.-D., Geiger, S.R., Baumli, S., Gartmann, M., Gerber, J., Jennebach, S., Mielke, T., Tschochner, H., Beckmann, R., and Cramer, P. (2007). Functional architecture of RNA polymerase I. Cell *131*, 1260–1272.

Kulak, N.A., Pichler, G., Paron, I., Nagaraj, N., and Mann, M. (2014). Minimal, encapsulated proteomic-sample processing applied to copy-number estimation in eukaryotic cells. Nat. Methods *11*, 319–324.

Lesne, A., Victor, J.-M., Bertrand, E., Basyuk, E., and Barbi, M. (2018). The Role of Supercoiling in the Motor Activity of RNA Polymerases. In Molecular Motors: Methods and Protocols, C. Lavelle, ed. (New York, NY: Springer New York), pp. 215–232.

Li, H., Handsaker, B., Wysoker, A., Fennell, T., Ruan, J., Homer, N., Marth, G., Abecasis, G., and Durbin, R.; 1000 Genome Project Data Processing Subgroup (2009). The Sequence Alignment/Map format and SAMtools. Bioinformatics *25*, 2078–2079.

Lisica, A., Engel, C., Jahnel, M., Roldán, É., Galburt, E.A., Cramer, P., and Grill, S.W. (2016). Mechanisms of backtrack recovery by RNA polymerases I and II. Proc. Natl. Acad. Sci. USA *113*, 2946–2951.

Longtine, M.S., McKenzie, A., 3rd, Demarini, D.J., Shah, N.G., Wach, A., Brachat, A., Philippsen, P., and Pringle, J.R. (1998). Additional modules for versatile and economical PCR-based gene deletion and modification in Saccharomyces cerevisiae. Yeast *14*, 953–961.

Lukačišin, M., Landon, M., and Jajoo, R. (2017). Sequence-specific thermodynamic properties of nucleic acids influence both transcriptional pausing and backtracking in yeast. PLoS ONE *12*, e0174066.

Ma, J., Bai, L., and Wang, M.D. (2013). Transcription under torsion. Science *340*, 1580–1583.

Machanick, P., and Bailey, T.L. (2011). MEME-ChIP: motif analysis of large DNA datasets. Bioinformatics *27*, 1696–1697.

Manzo, S.G., Hartono, S.R., Sanz, L.A., Marinello, J., De Biasi, S., Cossarizza, A., Capranico, G., and Chedin, F. (2018). DNA Topoisomerase I differentially modulates R-loops across the human genome. Genome Biol. *19*, 100.

Markham, N.R., and Zuker, M. (2008). UNAFold: software for nucleic acid folding and hybridization. Methods Mol. Biol. *453*, 3–31.

Mayer, A., di Iulio, J., Maleri, S., Eser, U., Vierstra, J., Reynolds, A., Sandstrom, R., Stamatoyannopoulos, J.A., and Churchman, L.S. (2015). Native elongating transcript sequencing reveals human transcriptional activity at nucleotide resolution. Cell *161*, 541–554.

Merz, K., Hondele, M., Goetze, H., Gmelch, K., Stoeckl, U., and Griesenbeck, J. (2008). Actively transcribed rRNA genes in S. cerevisiae are organized in a specialized chromatin associated with the high-mobility group protein Hmo1 and are largely devoid of histone molecules. Genes Dev. *22*, 1190–1204.

Milligan, L., Huynh-Thu, V.A., Delan-Forino, C., Tuck, A., Petfalski, E., Lombraña, R., Sanguinetti, G., Kudla, G., and Tollervey, D. (2016). Strand-specific, high-resolution mapping of modified RNA polymerase II. Mol. Syst. Biol. *12*, 874.

Neugebauer, K.M. (2019). Nascent RNA and the Coordination of Splicing with Transcription. Cold Spring Harb. Perspect. Biol. *11*, a032227.

Neyer, S., Kunz, M., Geiss, C., Hantsche, M., Hodirnau, V.-V., Seybert, A., Engel, C., Scheffer, M.P., Cramer, P., and Frangakis, A.S. (2016). Structure of RNA polymerase I transcribing ribosomal DNA genes. Nature *540*, 607–610.

Nojima, T., Gomes, T., Grosso, A.R.F., Kimura, H., Dye, M.J., Dhir, S., Carmo-Fonseca, M., and Proudfoot, N.J. (2015). Mammalian NET-Seq Reveals Genome-wide Nascent Transcription Coupled to RNA Processing. Cell *161*, 526–540.

Nomura, M. (1999). Regulation of ribosome biosynthesis in Escherichia coli and Saccharomyces cerevisiae: diversity and common principles. J. Bacteriol. *181*, 6857–6864.

Oakes, M.L., Siddiqi, I., French, S.L., Vu, L., Sato, M., Aris, J.P., Beyer, A.L., and Nomura, M. (2006). Role of histone deacetylase Rpd3 in regulating rRNA gene transcription and nucleolar structure in yeast. Mol. Cell. Biol. *26*, 3889–3901.

Osheim, Y.N., French, S.L., Keck, K.M., Champion, E.A., Spasov, K., Dragon, F., Baserga, S.J., and Beyer, A.L. (2004). Pre-18S ribosomal RNA is structurally compacted into the SSU processome prior to being cleaved from nascent transcripts in Saccharomyces cerevisiae. Mol. Cell *16*, 943–954.

Osheim, Y.N., French, S.L., Sikes, M.L., and Beyer, A.L. (2009). Electron microscope visualization of RNA transcription and processing in Saccharomyces cerevisiae by Miller chromatin spreading. Methods Mol. Biol. *464*, 55–69.

Phipps, K.R., Charette, J.M., and Baserga, S.J. (2011). The small subunit processome in ribosome biogenesis—progress and prospects. Wiley Interdisciplinary Reviews RNA *2*, 1–21.

Pilsl, M., Crucifix, C., Papai, G., Krupp, F., Steinbauer, R., Griesenbeck, J., Milkereit, P., Tschochner, H., and Schultz, P. (2016). Structure of the initiation-competent RNA polymerase I and its implication for transcription. Nat. Commun. *7*, 12126.

Pleiss, J.A., Whitworth, G.B., Bergkessel, M., and Guthrie, C. (2007). Transcript specificity in yeast pre-mRNA splicing revealed by mutations in core spliceosomal components. PLoS Biol. *5*, e90.

Prescott, E.M., Osheim, Y.N., Jones, H.S., Alen, C.M., Roan, J.G., Reeder, R.H., Beyer, A.L., and Proudfoot, N.J. (2004). Transcriptional termination by RNA polymerase I requires the small subunit Rpa12p. Proc. Natl. Acad. Sci. USA *101*, 6068–6073.

Quinlan, A.R., and Hall, I.M. (2010). BEDTools: a flexible suite of utilities for comparing genomic features. Bioinformatics *26*, 841–842.

Ramírez, F., Ryan, D.P., Grüning, B., Bhardwaj, V., Kilpert, F., Richter, A.S., Heyne, S., Dündar, F., and Manke, T. (2016). deepTools2: a next generation web server for deep-sequencing data analysis. Nucleic Acids Res. *44* (W1), W160-5.

Saldi, T., Cortazar, M.A., Sheridan, R.M., and Bentley, D.L. (2016). Coupling of RNA Polymerase II Transcription Elongation with Pre-mRNA Splicing. J. Mol. Biol. *428*, 2623–2635.

CellPress
OPEN ACCESS

Saldi, T., Fong, N., and Bentley, D.L. (2018). Transcription elongation rate affects nascent histone pre-mRNA folding and 3′ end processing. Genes Dev. *32*, 297–308.

Sandmeier, J.J., French, S., Osheim, Y., Cheung, W.L., Gallo, C.M., Beyer, A.L., and Smith, J.S. (2002). RPD3 is required for the inactivation of yeast ribosomal DNA genes in stationary phase. EMBO J. *21*, 4959–4968.

Sarkar, A., Léger, J.F., Chatenay, D., and Marko, J.F. (2001). Structural transitions in DNA driven by external force and torque. Phys. Rev. E Stat. Nonlin. Soft Matter Phys. *63*, 051903.

Schneider, D.A., French, S.L., Osheim, Y.N., Bailey, A.O., Vu, L., Dodd, J., Yates, J.R., Beyer, A.L., and Nomura, M. (2006). RNA polymerase II elongation factors Spt4p and Spt5p play roles in transcription elongation by RNA polymerase I and rRNA processing. Proc. Natl. Acad. Sci. USA *103*, 12707–12712.

Schwalb, B., Michel, M., Zacher, B., Frühauf, K., Demel, C., Tresch, A., Gagneur, J., and Cramer, P. (2016). TT-seq maps the human transient transcriptome. Science *352*, 1225–1228.

Shchepachev, V., Bresson, S., Spanos, C., Petfalski, E., Fischer, L., Rappsilber, J., and Tollervey, D. (2019). Defining the RNA interactome by total RNA-associated protein purification. Mol. Syst. Biol. *15*, e8689.

Sheridan, R.M., Fong, N., D'Alessandro, A., and Bentley, D.L. (2019). Widespread Backtracking by RNA Pol II Is a Major Effector of Gene Activation, 5′ Pause Release, Termination, and Transcription Elongation Rate. Mol. Cell *73*, 107–118.e4.

Sun, Q., Zhu, X., Qi, J., An, W., Lan, P., Tan, D., Chen, R., Wang, B., Zheng, S., Zhang, C., et al. (2017). Molecular architecture of the 90S small subunit preribosome. eLife *6*, e22086.

Tadigotla, V.R.O., O Maoiléidigh, D., Sengupta, A.M., Epshtein, V., Ebright, R.H., Nudler, E., and Ruckenstein, A.E. (2006). Thermodynamic and kinetic modeling of transcriptional pausing. Proc. Natl. Acad. Sci. USA *103*, 4439–4444.

Tafur, L., Sadian, Y., Hoffmann, N.A., Jakobi, A.J., Wetzel, R., Hagen, W.J.H., Sachse, C., and Müller, C.W. (2016). Molecular Structures of Transcribing RNA Polymerase I. Mol. Cell *64*, 1135–1143.

Tantale, K., Mueller, F., Kozulic-Pirher, A., Lesne, A., Victor, J.-M., Robert, M.-C., Capozi, S., Chouaib, R., Bäcker, V., Mateos-Langerak, J., et al. (2016). A single-molecule view of transcription reveals convoys of RNA polymerases and multi-scale bursting. Nat. Commun. *7*, 12248.

Tongaonkar, P., French, S.L., Oakes, M.L., Vu, L., Schneider, D.A., Beyer, A.L., and Nomura, M. (2005). Histones are required for transcription of yeast rRNA genes by RNA polymerase I. Proc. Natl. Acad. Sci. USA *102*, 10129–10134.

Toussaint, M., Levasseur, G., Tremblay, M., Paquette, M., and Conconi, A. (2005). Psoralen photocrosslinking, a tool to study the chromatin structure of RNA polymerase I–transcribed ribosomal genes. Biochem. Cell Biol. *83*, 449–459.

Turowski, T.W., and Tollervey, D. (2015). Cotranscriptional events in eukaryotic ribosome synthesis. Wiley Interdiscip. Rev. RNA *6*, 129–139.

Turowski, T.W., Leśniewska, E., Delan-Forino, C., Sayou, C., Boguta, M., and Tollervey, D. (2016). Global analysis of transcriptionally engaged yeast RNA polymerase III reveals extended tRNA transcripts. Genome Res. *26*, 933–944.

Viktorovskaya, O.V., and Schneider, D.A. (2015). Functional divergence of eukaryotic RNA polymerases: unique properties of RNA polymerase I suit its cellular role. Gene *556*, 19–26.

Vinayachandran, V., Reja, R., Rossi, M.J., Park, B., Rieber, L., Mittal, C., Mahony, S., and Pugh, B.F. (2018). Widespread and precise reprogramming of yeast protein-genome interactions in response to heat shock. Genome Res. *28*, 357–366.

Wachutka, L., Caizzi, L., Gagneur, J., and Cramer, P. (2019). Global donor and acceptor splicing site kinetics in human cells. eLife *8*, e45056.

Wahba, L., Costantino, L., Tan, F.J., Zimmer, A., and Koshland, D. (2016). S1-DRIP-seq identifies high expression and polyA tracts as major contributors to R-loop formation. Genes Dev. *30*, 1327–1338.

Wallace, E.W.J., and Beggs, J.D. (2017). Extremely fast and incredibly close: cotranscriptional splicing in budding yeast. RNA *23*, 601–610.

Webb, S., Hector, R.D., Kudla, G., and Granneman, S. (2014). PAR-CLIP data indicate that Nrd1-Nab3-dependent transcription termination regulates expression of hundreds of protein coding genes in yeast. Genome Biol. *15*, R8.

Wittner, M., Hamperl, S., Stöckl, U., Seufert, W., Tschochner, H., Milkereit, P., and Griesenbeck, J. (2011). Establishment and maintenance of alternative chromatin states at a multicopy gene locus. Cell *145*, 543–554.

Zamft, B., Bintu, L., Ishibashi, T., and Bustamante, C. (2012). Nascent RNA structure modulates the transcriptional dynamics of RNA polymerases. Proc. Natl. Acad. Sci. USA *109*, 8948–8953.

STAR★METHODS

KEY RESOURCES TABLE

REAGENT or RESOURCE	SOURCE	IDENTIFIER
Antibodies		
TAP Tag Polyclonal Antibody	Thermo Fisher Scientific	Cat#CAB1001; RRID:AB_10709700
Bacterial and Virus Strains		
Chemicals, Peptides, and Recombinant Proteins		
-Trp synthetic dropout mix	Formedium	Cat#DCS0149
Guanidine hydrochloride	Sigma	Cat#G4505-1KG
HaloTEV Protease	Promega	Cat#G6601
Critical Commercial Assays		
cOmplete EDTA-free protease inhibitor cocktail tablets	Roche	Cat#11873580001
Ni-NTA Superflow	QIAGEN	Cat#30410
Pierce spin columns snap cap	Thermo Scientific	Cat#69725
RNace-It Ribonuclease cocktail	Agilent	Cat#400720
RNasin Ribonuclease Inhibitor	Promega	Cat#N2115
Recombinant RNasin Ribonuclease Inhibitor	Promega	Cat#N2511
DNase RQ1	Promega	Cat#M6101
T4 RNA Ligase 2, truncated K227Q	NEB	Cat#M0351
T4 RNA Ligase 1	NEB	Cat#M0204L
T4 PNK	NEB	Cat#M0201L
Nitrocellulose membranes	GE Healthcare	Cat#10 439 196
MetaPhor agarose	Lonza	Cat#50180
NuPAGE 4-12% polyacrylamide Bis-Tris Gels	Life Technologies	Cat#NP0335
NuPAGE LDS 4x sample buffer	Life Technologies	Cat#NP0007
NuPAGE SDS-MOPS running buffer	Life Technologies	Cat#NP0001
NuPAGE Transfer Buffer	Life Technologies	Cat#NP00061
MinElute Gel Extraction kit	QIAGEN	Cat#28604
Proteinase K	Roche	Cat#03115836001
RNase H	NEB	Cat#M0297L
LA Taq	Takara	Cat#RR002M
Deposited Data		
Raw data files from CRAC	NCBI Gene expression omnibus	GSE136056
Raw image files	Mendeley	https://doi.org/10.17632/m253kk9sm6.1
Experimental Models: Organisms/Strains		
S. cerevisiae Strain background: BY4741 (MATa his3Δ1 leu2Δ0 met15Δ0 ura3Δ0)	Longtine et al., 1998	yTWT001
S. cerevisiae Strain Rpa190HTP a his3Δ1 leu2Δ0 met15Δ0 ura3Δ0 RPA190-HTP::URA3MX	This study	yTWT046
S. cerevisiae Strain Rpa135 HTP a his3Δ1 leu2Δ0 met15Δ0 ura3Δ0 RPA135-HTP::URA3MX	This study	yTWT051
S. cerevisiae Strain Rpa135 HTP Rpa12ΔC a his3Δ1 leu2Δ0 met15Δ0 ura3Δ0 RPA12(1-74aa only) RPA135-HTP::URA3MX	This study	yTWT232
S. cerevisiae Strain Rpa190 HTP 25 rDNA a ade2-1 ura3-1 his3-11,15 trp1-1 leu2-3,112 can1-100 fob1Δ::HIS3 RPA190-HTP::URA3MX	This study	yTWT144

(Continued on next page)

Continued

REAGENT or RESOURCE	SOURCE	IDENTIFIER
Oligonucleotides		
Table S3	This study	N/A
Software and Algorithms		
PyCRAC	Webb et al., 2014	https://bitbucket.org/sgrann/pycrac
SAMtools v1.3.1	Li et al., 2009	http://www.htslib.org/; RRID:SCR_002105
Bedtools v2.25	Quinlan and Hall, 2010	https://github.com/arq5x/bedtools2; RRID:SCR_006646
Prism 7	Graphpad	https://www.graphpad.com/; RRID:SCR_002798
Integrative Genomics Viewer	Broad Institute	http://software.broadinstitute.org/software/igv/; RRID:SCR_011793
Novoalign v2.07.00	Novocraft	http://www.novocraft.com/products/novoalign/; RRID:SCR_014818
UNAfold package v3.8	Markham and Zuker, 2008	http://unafold.rna.albany.edu/; RRID:SCR_001360

RESCOURCE AVAILABILITY

Lead Contact

Further information and requests for resources and reagents should be directed to and will be fulfilled by the Lead Contact, David Tollervey (d.tollervey@ed.ac.uk).

Materials Availability

All unique/stable reagents generated in this study are available from the Lead Contact without restriction.

Data and Code Availability

The accession number for the RNA sequencing data reported in this paper is GEO: [GSE136056]. Original data have been deposited to Mendeley Data: [https://doi.org/10.17632/m253kk9sm6.1].

The full MATLAB code for the mathematical model has been submitted as a git repository: https://bitbucket.org/bdgoddard/rnap_public/src/master/.

EXPERIMENTAL MODEL AND SUBJECT DETAILS

Strains

Yeast analyses were performed in strains derived from BY4741 (*MAT*a; *his3Δ1*; *leu2Δ0*; *met15Δ0*; *ura3Δ0*), except for the 25 rDNA strain which derives from NOY1071 (Cioci et al., 2003). For CRAC analyses, cells were grown in synthetic medium with 2% glucose at 30°C. For Miller spreads, cells were grown in YPD medium + 1M sorbitol. Strains used are listed above. Oligonucleotides are listed in Table S3.

METHOD DETAILS

Miller Chromatin Spreads and Measurements of Polymerase Positions

Starter cultures of yeast strain BY4741 were diluted into YPD (yeast extract, peptone, glucose) medium + 1 M sorbitol such that after growth at 30°C for 6 h the culture reached a density of OD_{600} = 0.4. At that point 1 mL aliquots were harvested and Miller chromatin spreads were prepared for electron microscopy as described (Osheim et al., 2009). In brief, pelleted cells were lysed using hypotonic shock; cell contents were allowed to disperse with gentle swirling; and the resultant "spread" was centrifuged onto a carbon coated EM grid. Staining with phosphotungstic acid and uranyl acetate enhanced the contrast of the spread material.

Chromatin spreads on multiple grids from several cultures were methodically examined, grid square by grid square, using a JEOL 100CX transmission electron microscope. Areas of dispersed chromatin containing 35S rRNA genes were photographed. In Miller chromatin spreads, active rRNA genes are recognized as a consequence of their high transcription frequency. The multiple Pol I molecules engaged in transcribing the genes lend electron density to the DNA template thus enhancing the visibility of the genes.

Micrographs of chromatin were examined and all 35S genes that could be unambiguously traced from 5′ to 3′ ends were scanned on an Epson Perfection V750 Pro flatbed scanner. Polymerase positions were measured on these digital images using ImageJ software. The position of the center of each polymerase was recorded relative to the 5′ end of the gene on which it was observed. These positions, measured in pixels along the DNA strands, were then normalized by setting the position of the first polymerase to a value of 0 and that of the last polymerase to a value of 100. While we cannot exactly determine if a first polymerase is at the promoter or occupies a spot a

polymerase width or two downstream, we based our determination of "full length" genes on the distance between first and last polymerase and the relative distances between upstream and downstream 5S genes, neighboring 35S genes, together with the characteristic features of polymerases and transcripts associated with initial and final positions (French et al., 2008; Osheim et al., 2004)

In-vivo RNA crosslinking

Strains for CRAC experiments were grown in synthetic dextrose (SD) medium with 2% glucose, lacking Trp to OD_{600} = 0.5. Actively growing cells were cross-linked in culture media using megatron UVC cross-linker (Granneman et al., 2011) typically for 100 s or less when indicated. For PAR-CRAC medium was additionally supplemented with 4-thiouracil (4tU) using a UVA-box (Shchepachev et al., 2019) for 40 s. 4tU was added to 1 mM final concentration for 30 min and cross-linked without washing or to 3.3 mM final concentration for 15 min, washed with PBS and immediately cross-linked.

CRAC

Samples were processed as previously described (Turowski et al., 2016). However, phosphatase treatment was omitted, so the 3′-OH ends required for linker ligation are present only on nascent RNA transcripts. Cells were lysed in TNMC100 (50 mM Tris-HCl pH 7.5, 150 mM NaCl, 0.1% NP-40, 5 mM $MgCl_2$, 10 mM $CaCl_2$, 5 mM β-mercaptoethanol, 50U of DNase RQ1 and a protease-inhibitor cocktail (1 tablet / 50 mL) with zirconia beads in a 50 mL conical. The cells were lysed with five one-minute pulses, with cooling on ice in between. The supernatant was spun for 20 minutes at 21,000 g. The cleared lysate was incubated with the IgG Sepharose for two hours at 4°C, with nutating. Subsequently, the beads were washed three times with TMN600 (50 mM Tris-HCl pH 7.5, 600 mM NaCl, 0.1% NP-40, 1.5 mM $MgCl_2$) and two times TMN100 (50 mM Tris-HCl pH 7.5, 100 mM NaCl, 0.1% NP-40, 5 mM $MgCl_2$). The eluate was transferred to a fresh tube containing 350 μL TMN100, 2.5U of RNace-IT was added and samples were incubated for 5 minutes at 37°C to fragment protein-bound RNA.

Protein:RNA complexes were eluted by incubation with HaloTEV for 2h at 18°C with shaking. The supernatant was separated and adjusted for nickel affinity purification with the addition of 400 mg guanidine hydrochloride, 45 μL NaCl (3M) and 7 μL imidazole (1 M) and added to 50 μL of washed nickel beads.

Following 4h incubation, the nickel beads were washed three times with WBI (6.0 M guanidine hydrochloride, 50 mM Tris-HCl pH 7.5, 300 mM NaCl, 0.1% NP-40, 10 mM imidazole, 1.5 mM $MgCl_2$ and 5 mM β-mercaptoethanol), three times with PNK buffer (50mM Tris-HCl pH 7.5, 50 mM NaCl, 1.5 mM MgCl2, 0.1% NP-40, and 5 mM β-mercaptoethanol) and transferred to a spin column. Subsequent reactions (80 μL total volume for each) were performed in the columns, and afterward washed once with WBI and three times with PNK buffer:

1. 3′ linker ligation (1x PNK buffer(NEB), 10% PEG8000, 20U T4 RNA Ligase II truncated K227Q, 80U RNasIN, 80 pmol preadenylated 3′ miRCat-33 linker (IDT); 16°C overnight).
2. 5′ end phosphorylation and radiolabeling (1x PNK buffer (NEB), 40 U T4 PNK (NEB), 80U RNasIN, 40 μCi ^{32}P-γATP; 37°C for 45 min, with addition of 100 nmol of ATP after 30 min).
3. 5′ linker ligation (1x PNK buffer (NEB), 10% PEG8000, 40 U T4 RNA ligase I (NEB), 80 U RNasIN, linker, 200 pmol 5′ linker, 1 mM ATP; 22°C for 4h).

The beads were washed twice with WBI and three times with PNK buffer. Protein:RNA complexes were eluted in 200 μL of elution buffer (50 mM Tris-HCl pH 7.5, 50 mM NaCl, 0.1% NP-40, 300 mM imidazole, and 5 mM β-mercaptoethanol) and acetone precipitated overnight. RNPs were pelleted at 21000 g for 20 minutes, and resuspended in 20 μL 1X NuPAGE sample loading buffer supplemented with 8% β-mercaptoethanol. The sample was denatured by incubation at 65°C for 10 minutes, and run on a 4%–12% Bis-tris NuPAGE gel at 130 V. The protein:RNA complexes were transferred to Hybond-C nitrocellulose membranes with NuPAGE MOPS transfer buffer for 2 h at 100V.

Labeled RNA was detected by autoradiography. The appropriate region was excised from the membrane and treated with 0.2 μg/μL Proteinase K (50 mM Tris-HCl pH 7.5, 50 mM NaCl, 0.1% NP-40, 10 mM imidazole, 1% SDS, 5 mM EDTA, and 5 mM β-mercaptoethanol; 2 hr 55°C with shaking) in a 500 μL reaction. The RNA component was isolated with a standard phenol:chloroform extraction followed by ethanol precipitation with 1 μL of GlycoBlue. The RNA was reverse transcribed using Superscript III and the miRCat-33 RT oligo (IDT) for 1 hr at 50°C in a 20 μL reaction. The resulting cDNA was amplified by PCR in 50 μL reactions using La Taq (5 μL template, 21-26 cycles) PCR reactions were combined, precipitated in ethanol, and resolved on a 3% Metaphore agarose gel. A region corresponding to 140 to 200 bp was excised from the gel and extracted using the Min-elute kit. Libraries were measured with Qbit and sequenced using Illumina HiSeq with 50bp single-end reads or Illumina MiniSeq with 75bp single-end reads.

Purification of RNA polymerase I and *in vitro* assay

The protein content of the supernatant was determined using the Bradford assay. Equal protein amounts (usually 1 ml cell extract, 20–30 mg) were incubated with 50–75 μl of immunoglobulin-G (rabbit IgG, I5006, Sigma) coupled magnetic beads slurry (Dynabeads M-270 Epoxy, 300 mg) for 1–2 h on a rotating wheel. The beads had previously been equilibrated with lysis buffer. The beads were washed four times with 1 ml buffer B1500 (20 mM HEPES/KOH pH 7.8, 1.5 M KOAc, 1 mM MgCl2, 20% glycerol, 0.1% IGEPAL CA-

630) and three times with 1 ml buffer B200 (20 mM HEPES/KOH pH 7.8, 200 mM KAc, 1 mM $MgCl_2$, 20% glycerol). For elution, beads were finally resuspended in 100 μl of buffer B200, supplemented with 3 μl TEV protease (HaloTEV, Promega G6602) and incubated for 2 h at 16 °C in a thermomixer (1,000 rpm). The supernatant was collected and aliquots were stored at 4°C for short term or at −80 °C for longer. For buffer exchange assays, TEV elution was skipped and aliquots were stored only for short term at 4°C. 10% of the purified fraction was analyzed via SDS–PAGE to monitor the purification success. Protein concentrations were determined by comparing the intensity of Coomassie-stained RNA polymerase subunits to the defined amount of Coomassie-stained HaloTEV protease used.

The *in vitro* RNA extension assay was modified from (Engel et al., 2013; Kuhn et al., 2007). For 1 reaction, 2 pmol of annealed RNA-DNA-DNA scaffold was pre-incubated with ~2 pmol of purified enzyme for 20 min at 20°C. Transcription was started by adding 6 μL 2x transcription buffer (TB). Elongation was performed in 1x TB (60 mM $(NH_4)_2SO_4$, 20 mM HEPES/KOH pH 7.6, 8 mM $MgSO_4$, 10 μM $ZnCl_2$, 10% glycerol, 10 mM DTT) supplemented with 1 mM NTPs. The samples were incubated at 28°C for 5 min. For backtracking assays, reaction tubes were placed on a magnetic rack, and supernatant was removed. Beads were washed with 200 μL buffer B200, re-suspended in 12 μL 1x TB without NTPs and incubated at 28°C for 10 min. All reactions were stopped by addition of 2x RNA loading dye (Thermo, R0641). Samples were heat denatured at 95°C for 5 min and resolved on 8 M urea 20% polyacrylamide gels. Fluorescently labeled transcripts were visualized using a Fugi FLA-5100 phosphoImager and images were processed using Multi Gauge software (Fuji).

Validation of RNAPI CRAC data

Two major aspects of the CRAC data were investigated: Contamination with mature rRNA or processed pre-rRNA and bias in sequence recovery.

In total RNA, mature rRNAs (18S, 5.8S and 25S rRNA) are much more abundant than the spacer regions (5′ETS, ITS1, ITS2, 3′ETS) present in the nascent transcript. However, the recovery of reads mapping to the rRNA sequences was not clearly elevated relative to the spacers and there was no accumulation at the mature rRNA boundaries (Figure 1E). This shows that the RNAPI CRAC data are not significantly contaminated by mature rRNAs.

During pre-rRNA transcription, the nascent transcript is cleaved at four sites; A0, A1, A2 and B0. Cleavages at A0-A2 are coupled and predominately cleaved in the nascent transcript, but processing occurs when RNAPI has traveled ~1.2Kb downstream of site A2 (Axt et al., 2014; Kos and Tollervey, 2010). Sequences terminating at sites A0, A1 and A2 were not elevated in the CRAC data (Figures 1E and S1C), confirming that the processed pre-rRNAs were not recovered. Mapped 3′ ends from cDNAs are therefore expected to represent the positions of bona fide 3′ ends in nascent transcripts.

We also performed experiments to validate the Rpa190 CRAC data and detect potential bias in target recovery. Notably, all of these analyses yielded RNAPI distributions that were consistent with the results of CRAC with Rpa190 (Figure 1F).

1: To reduce the possibility of non-specific cross-linking to Rpa190, we analyzed a range of shorter UVC cross-linking times. These showed minimal changes (Figure S1D).

2: To exclude steric preferences in RNA cross-linking, we HTP-tagged the second largest subunit of RNAPI, Rpa135 (Figure S1E). This showed a similar 5′ bias to Rpa190, and substantial overlap at the level of individual peaks, as shown by a peak metaplot (Figure S1E, embedded panel; see Figure S1F and STAR Methods for details on peak metaplot generation).

3: We performed a PAR-CRAC experiment, in which RNA was metabolically labeled with 4-thiouracil (4SU) and cross-linked using UVA (Figure S1G). 4SU crosslinking involves different photochemistry and may be less prone to recover non-specific crosslinking relative to UVC (Shchepachev et al., 2019). The peak metaplot for PAR-CRAC was very similar to the CRAC data (Figure S1G). However, some enrichment for U-rich sites was observed in PAR-CRAC, as expected (Figure S1H).

4: Wild-type yeast strains generally have ~150-200 ribosomal repeats, of which around 50% are reported to be actively transcribed, making it conceivable that the apparent 5′ end bias (Figure 1E) arises from premature termination on "inactive" repeats. To test this possibility, Rpa190 CRAC was performed in a yeast strain with only 25 rDNA repeats, all of which are highly transcribed. The RNAPI profile in this strain was almost identical to the wild-type (Figure S1I).

5: We considered the possibility that RNA interacting with the outside of the polymerase might contribute to the signals, although the requirement that recovered RNA has a 3′ OH group made this unlikely. To test this, we considered only cDNA sequences shorter than 20 nt, since this region will be almost entirely located within the transcription bubble and RNA exit channel. This analysis also revealed the distinctive peaks for RNAPI distribution (Figure S1J).

6: To address the possibility that periodic peaks with the 5′ ETS are generated by ambiguous mapping to repetitive sequences, we prepared a reference genome with single copy of the rDNA. We then performed unambiguous mapping, which does not report any sequences that map to more than one location. Unambiguous mapping returned a spiky profile, closely matching the results of random mapping (Figure S1K), showing that mis-mapping does not make a major contribution.

7: We considered that the distribution of RNAPI might be influenced by chromatin structure. The actively transcribed rDNA repeats are associated with the DNA binding protein Hmo1, which is related to human HMG1 (Hall et al., 2006; Merz et al., 2008; Wittner et al., 2011). In addition, DNA torsion can also be relieved by writhe, which might be promoted by toroid formation, constrained by DNA-binding proteins such as Hmo1. Rpa190 CRAC was performed in an *hmo1Δ* strain (Figures S2A and S2B) but still showed a 5′ bias and stable peaks over the 5′ region of the rDNA.

8: Finally, we considered bias originating from the CRAC experimental protocol. Mainly the relationship between nascent RNA recovery, RNA structure, UV crosslinking and adaptor ligation steps during library preparations. The arguments against this hypothesis are as follows: (1) nascent RNA interacting with RNAP is buried inside the channel in its extended, unstructured form, therefore, there should be no influence of structure on the UV crosslinking efficiency. (2) The CRAC protocol involves highly denaturing conditions to reduce the background. Following protein denaturation, the RNA could be susceptible to folding, potentially sequestering RNA ends and hindering adaptor ligation. In such a case we would expect lower recovery of highly structured RNAs, e.g., hairpin loop regions. However, this is in marked contrast to our results (Figure S2F).

From this validation we conclude that CRAC approximates the genuine distribution of RNAPI at most sites along the rDNA transcription unit *in vivo*. All subsequent analysis was performed using the median of six biological replicates (Figure S1L). Moreover, we generated randomized datasets and compared RNAPI CRAC with them using a Spearman test (Figure S1M). This revealed that RNAPI CRAC data present a non-random distribution.

Development of Mathematical model for RNAPI transcription

The numerical model for elongation steps of RNAPI transcription kinetics, was developed using input data taken from biological experiments wherever possible (Table S1).

Justification of parameters of the model

1. Quantification of molecules of RNA polymerases

To estimate total copy numbers for RNAPI, RNAPII and RNAPIII, we re-analyzed three independent studies: (Chong et al., 2015; Ghaemmaghami et al., 2003; Kulak et al., 2014). An average and median for all subunits were calculated for each RNA polymerase (Figure S4A). These calculations were repeated for all specific subunits for each RNA polymerase and presented similar trend. Data expressed in arbitrary units (Chong et al., 2015) were used only to confirm ratios between RNA polymerases. Analysis of these data indicated that RNAPI and II are present at similar levels of 5,000 - 6,000 molecules per cell, whereas RNAPIII is present in 2,500-3,000 copies.

2. Transcription initiation rate

Rapidly dividing yeast cells produce ~200,000 ribosomes per generation (~100 min), corresponding to ~2,000 ribosomes min^{-1}. There are ~150-200 rDNA repeats, of which ~50% are transcriptionally active (Dammann et al., 1993). Each transcription unit should therefore release ~20-27 completed pre-rRNA transcripts per minute (1 transcript every 2.2 - 3 s). The transcription initiation rate cannot therefore be less than 1 initiation per 2.2 - 3 s, but might be greater if the processivity of RNAPI is less than 100% or the elongation rate is non-uniform.

Transcription initiation by RNAPI has undoubtedly evolved to be extremely efficient. We postulate that polymerase may be recruited to the rDNA promoter faster than the time needed for the previous polymerase to clear the initiation site, making promoter clearance rate limiting. Therefore, we modeled RNAPI transcription initiation as a stochastic process with a success probability between 0.33 and 1.0 s^{-1}. We tested rates of stochastic initiation over this range, limited by the requirement that the preceding RNAPI has cleared the initiation region. A mean stochastic initiation rate of 0.8 s^{-1} generated RNAPI loading consistent with data from Miller spreads (~50 per rDNA unit) (Figure S4I).

3. RNAPI number per rDNA transcription unit and RNAPI spacing

The maximum average number of RNAPI molecules per rDNA transcription unit can be estimated from the number of RNAPI in the cell (5,000-6,000 molecules) and rDNA repeats (75-100), giving a range of 50-60. This figure is in good agreement with quantification of RNAPI complexes from Miller chromatin spreads (~50; Table S1). The number of RNAPI molecules on the 7 Kb long rDNA transcription unit gives an average RNAPI spacing of 120-140 nt.

$$spacing = \frac{7000\ nt}{\left(\frac{RNAPI\ molecules}{active\ rDNA\ repeats}\right)}$$

This value is in good agreement to independent calculations derived from metabolic labeling experiment (Kos and Tollervey, 2010). The average velocity of RNAPI (40 nt sec^{-1}) and transcript release rate (1 per 3 s; from initial calculations above) predicts a spacing of 120 bp. Measurements of the relative positions of RNAPI in Miller spreads by tomography, indicated minimal center to center separation of 15 nm (Neyer et al., 2016), which is estimated to reflect a 44 bp. This figure may therefore represent a minimal spacing between RNAPI molecules *in vivo*.

4. Elongation rate of RNAP in the discrete model

The velocities of RNA polymerases have been determined *in vivo* and *in vitro* many times and some examples were summarized in Table S2. Interestingly *in vitro* measurements are systematically lower than *in vivo*.

Approximate RNAPI elongation rates can be also obtained from number of ribosomes produced per generation (200,000), yeast doubling time (100 min), pre-rRNA length (~7000 nt) and number of transcribing RNAPI molecules (5000 - 6000).

$$V_{RNAPI} = \frac{200\,000\ ribosomes \cdot 7000\ nt}{6000\ seconds \cdot RNAPI\ molecules}$$

Based on the published data, ~40 nt·sec^{-1} is expected to be the overall average velocity of transcribing RNAPI. However, pause-free elongation is very unlikely *in vivo*. Therefore, we used 50 nt sec^{-1} as the intrinsic, average RNAPI elongation rate (V_{Int}) in our model.

Analysis of elongation *in vitro* determined the distribution of nucleotide incorporation (elongation) rates at a nucleotide level in *E. coli* using single-molecule measurements (Adelman et al., 2002). These rates directly reflect the range of time delays before elongation or backtracking. It is described by two Gaussian functions: The first function, comprising 7.8% of the area, represents the paused state and is centered at 0.9 nt sec^{-1} (Figure S4B, red line). The second function reflects active elongation and centered at 12.8 nt sec^{-1} (Figure S4B, green line). We adopted this function for RNAPI, using an *in vivo* elongation velocity centered at 50 nt sec^{-1} (Figure S4B').

RNA polymerase elongation is based on a Brownian ratchet mechanism, in which each step of elongation and catalysis is discrete and independent from other steps. Classical mechanics and momentum do not apply to molecular processes, and we therefore constructed a stochastic and discrete model. At each time step, there is a probability of moving 0, +1 or −1 nucleotides according to the distribution presented in Figure S4B'.

$$V_{Elongation} = N\left(V_{Int}, (\sigma \cdot V_{Int})^2\right), \text{where } V_{Int} = 50 \text{ and } \sigma = 0.4$$

$$V_{Paused} = N\left(V_{Paused}, \sigma_{Paused}{}^2\right), \text{where } V_{Paused} = 0.9 \text{ and } \sigma_{Paused} = 1.5$$

For each particle, with probability $p = 0.078$, V_{Random} is drawn from the V_{paused} distribution, and otherwise from the $V_{elongation}$ distribution.

Having computed the velocity V_{Random} for a given time step of length dt, the corresponding probability of jumping in that time step is given by $p = |V| \cdot dt$. This is essentially the expected distance moved in one time-step. With probability p, the RNAP jumps in the direction of V in that time step.

This probability can be modified by following factors:

(a) DNA torsion
(b) Promotion of RNAP elongation by nascent structure forming behind the polymerase
(c) Decrease of RNAP elongation by a strong RNA:DNA hybrid within the transcription bubble.

5. RNAP convoys imply DNA torsion effects

RNAP elongation along a DNA helix requires two types of movement: forward and rotary. In principal, either the DNA or polymerase can rotate with a frequency of ~240 rpm. However, the combined mass of all polymerases plus nascent pre-ribosomes is very much greater than that of the rDNA. The rDNA is nucleosome-free and loaded with multiple RNAPI complexes (~50 at 0.5 MDa each), each associated with up to 7 Kb of pre-rRNA transcript (up to 2.3 MDa) and a multi-megadalton pre-ribosome (6 MDa for the SSU processome alone) containing many assembly factors (Turowski and Tollervey, 2015). The difficulty of moving these very large complexes through the highly viscous nucleolus environment (Bormuth et al., 2009), and steric problems that would be entailed by rapid rotation of the pre-rRNA around the DNA, make it very likely that the rDNA is rotated through an array of polymerases, in agreement with the model of immobilized RNAP (Iborra et al., 1996).

If a group of RNAPI complexes move along the DNA together, this will not result in over- or under-winding of the DNA. This suggests that the RNAPI array on the rDNA acts cooperatively to rotate the DNA template. DNA topoisomerases I and II (Top1 and Top2) can relax positive or negative supercoils and are necessary to maintain transcription of the rDNA (Brill et al., 1987; El Hage et al., 2010). However, the abundance of Top1 is estimated to be very much lower than the RNA polymerases. Quantification reported by SGD (https://www.yeastgenome.org) based on multiple analyses: Top1; 4130 ± 2517 molecules per cell. Sum of the largest subunits of all three RNA polymerases; 33674 ± 12715. Moreover, topoisomerases can unwind a minimum of one complete turn of the DNA, whereas a stalling force is generated by substantially less overwinding for polymerases with spacing typical for the rDNA (120 bp) (Heberling et al., 2016; Ma et al., 2013; Tantale et al., 2016).

We therefore propose that RNAPI complexes move as a group along single rDNA transcription unit while DNA rotates through the polymerases. Notably, similar models have been proposed for highly transcribed RNAPII genes associated with "convoys" of RNA polymerases resulting from transcriptional bursting (Lesne et al., 2018; Tantale et al., 2016), and for bacterial polymerase (Heberling et al., 2016; Kim et al., 2019). Finally, DNA rotation during transcription was observed directly in *E. coli* RNAP (Harada et al., 2001).

In the model for RNAP convoys, the distance between initially loaded RNAP molecules is maintained by torsion in the DNA helix. Transcription elongation of RNAP includes a translocation step based on Brownian motion. Only this step is assumed to be force sensitive (Dangkulwanich et al., 2013). Single-molecule elongation of bacterial RNAP can be stopped *in vitro* by application of a stalling force of 15-25 pN (Bustamante et al., 2004).

Overwinding and underwinding of DNA (σ) generates force. $\sigma = 0.00$ (0%) when DNA is relaxed (1 turn / 10.5 bp, 10 turns / 105 bp) and $\sigma = 0.10$ (10%) when DNA is 10% overwound (1.1 turn / 10.5 bp, 11 turns / 105 bp). When all polymerases within the convoy are moving along DNA with the same velocity (relative velocity $v_{rel} = 0$) the force generated by DNA torsion equals 0. However, when one polymerase moves faster than its neighbors ($v_{rel} > 0$), this results in DNA overwinding in front of RNAP and underwinding behind it (Figure 4A). Both of these effects will favor slowing of the middle RNAP.

A DNA torque $\tau = 11\ nm \cdot pN$ was reported to stall bacterial RNAP *in vitro* (Ma et al., 2013). [Note that DNA torque and stalling force have different units.] An elegant solution was proposed to calculate a relationship between DNA torque τ and DNA overwind σ (Figure S4C; Heberling et al., 2016).

$$\tau = \frac{\mu \pi^2 r^4}{10.5}\left[\ln\left(\frac{xP_{Init}}{dxP}\right) - \ln\left(\frac{xM_{Init}}{dxM}\right)\right],$$

Where $\mu = 300\ pN/nm^2$ is the shear modulus for DNA, $r = 1\ nm$ is the radius of DNA, 10.5 is number of bases per turn. σ is relationship between loading distance xP_{Init} or xM_{Init} and current distance dxP or dxM. The current distance is calculated as $dxP = x_{n+1} - x_n$ and $dxM = x_n - x_{n-1}$ (Figure 4A).

The force F acting on the RNAP is calculated from DNA torque τ as previously described (Ma et al., 2013; Figure S4D).

$F = (\tau \cdot \theta / d)$, where θ represents angular rotation of RNAP after 1 bp translocation 0.6 radian or 34°, converted from 10.5 bp per turn. d is the contour length of DNA per bp (~0.34 nm).

It is notable that the value of sigma σ causing RNAP stalling will be higher *in vivo* due to following reasons: (1) We assumed that highly packed and viscous environment of the nucleolus causes RNAPI to transcribe as a convoy. However, *in vivo* the ability of RNAPI to rotate around the rDNA will be greater than zero. Therefore, an increased limit of sigma σ includes this capacity to spin around the rDNA without introducing an additional parameter. (2) The average velocities of bacterial RNAP or RNAPI *in vivo* are $\geq$ 2 fold higher than *in vitro* (Table S2). The previously developed function describing bacterial RNAP velocity in relation to DNA torque τ is based on *in vitro* data, hence we assume that RNAPI stalling force *in vivo* is higher and decided to increase sigma σ appropriately.

We therefore used $\sigma = \pm 0.05$ as a parameter in our model. Much higher values are unlikely since $\sigma = \pm 0.20$ can lead to phase transition (Sarkar et al., 2001) and very low negative torque τ may lead to DNA melting.

In the model, DNA torsion modifies V_{Random} as follows:

$$V_{Random+Torque} = V_{Random} + c\left(1 - \frac{xP_{Init} - b}{dxP - b}\right) - c\left(1 - \frac{xM_{Init} - b}{dxM - b}\right)$$

Where xP_{Init} or xM_{Init} are initial distances between polymerases when initiated (engaged on the DNA), dxP or dxM are current distances between polymerases, b is the length of the transcription bubble (11 nt for RNAPI, PDB: 5M5X (Tafur et al., 2016)) and c is a constant describing DNA stiffness. In the basic model for RNAP convoys, the initial separation of RNAP is established by the initiation rate, and then maintained by DNA torque.

6. Range of DNA stiffness constant c

The $V_{Random+Torque}$ equation allows calculation of the DNA stiffness constant c in relation to DNA overwind σ. We use a simplified system of three RNAPs with an initial separation of 100 nt (as Figure 4A). Then we solved the equation, in which a given value for c should be strong enough to stop RNAP transcribing with average velocity V_{Int} when % of DNA overwind σ is equal to r:

$$c = \frac{-V_{Int}}{\frac{100}{100+r} - \frac{100}{100-r}}$$

V_{Int} is the intrinsic velocity of RNAPI and equals 50 $nt \cdot sec^{-1}$. This gives values of DNA stiffness constant c for a given velocity V_{Int} (Figure S4E). Based on these calculations the model used a DNA constant of $c = 500$.

7. Low Entrainment Region

The RNAP convoy model is justified by the energetic cost of spinning the DNA, friction, and the ratio between topoisomerases and all three eukaryotic RNAPs. Theoretically, only two flanking topoisomerases might be sufficient act as swivels to release torsion generated from DNA rotation by an entire convoy of RNAP. Notably, experimental data demonstrated that depletion of both topoisomerases causes severe perturbation in rDNA transcription when RNAPI is around 2 Kb into the transcription unit (El Hage et al., 2010). This was shown using a range of methods including northern hybridization, ChIP and chromatin spreads. Decreased Top1 activity is accompanied by an increased number of R-loops, as also observed in the human rDNA (Manzo et al., 2018).

CellPress
OPEN ACCESS

We interpret this observation as showing that RNAPI molecules are initially able to spin around the DNA, allowing changes in their relative positions without generating torsion, but become locked by torsion at around +2 Kb. To incorporate this mechanism into the model, we progressively engaged torsion within RNAP convoys over the initial 2 Kb of the rDNA. We used a linear engagement scheme, where at position 0 RNAP moves according to discrete stochastic elongation, at position 1000 DNA torque was applied in 50% and becomes fully engaged at position 2000 and later. This Low Entrainment Region was implemented as three elements: (1) Decreased DNA stiffness constant *c*. (2) Reset of x_{Init} position. (3) A small decrease in the intrinsic RNAPI velocity ($\leq$20%) to mimic the cost of friction. All three elements were applied progressively.

8. Role of nascent RNA in transcription elongation

Finally, we introduced our findings on the effects of sequence in the RNA:DNA hybrid within the transcription bubble and the structure of the extruded RNA into the model.

Nascent RNA interacts with template strand of DNA within transcription bubble. Stronger hybrids usually have a higher G+C content, particularly with G in the DNA sequence, and this correlates with slower RNAP translocation. We calculated the ΔG of RNA:DNA hybrids over an 8 nt rolling window ($dG^{RNA:DNA}$) along the rDNA as previously described (see El Hage et al., 2014; Turowski et al., 2016).

The folding energy of nascent RNA was calculated using a 65 nt rolling window, offset by 15 nt ($dG^{Structure}$), as described in Materials and Methods. Stronger structures limit translocation backward and promote translocation forward. Hence, RNA structures adjacent to RNAPI would act on elongation rate positively. On the basis of the backtracking assay (Figure 3) we applied this parameter only for structures with folding energy below the threshold value ($\Delta G \leq -11$ kcal·mol^{-1}). This excluded an artificial situation when long, but very weak structures would apparently have a sufficiently low ΔG to promote translocation.

Both values were incorporated into a model as modifiers of RNAP jump probability as follows:

$$V = V_{Random+Torque} + dG^{Structure}_{Strength} \cdot dG^{Structure} - dG^{RNA:DNA}_{Strength} \cdot dG^{RNA:DNA}$$

Values of strengths were fitted. Further details are in the model optimization section.

9. Optional elements of the model

A number of additional factors were considered during development of the model:

a) Topoisomerase activity and DNA looping

In our model topoisomerases induce single-strand cuts to spin DNA when a convoy of RNAP generates sufficient rotating force. The canonical role of topoisomerases is associated with resolving DNA supercoiling and we tested this possibility. Top1 can unwind a minimum of one complete turn of DNA. Therefore, we applied Top1 activity as a probability function of resolving a complete turn when distance between adjacent RNAP particles was greater than 25 nt. As demonstrated on Figure S4F Top1 activity has minor effect on the overall profile.

Notably, implementation of DNA looping into the model would be numerically similar to topoisomerase. We therefore predict that incorporation of DNA looping would have effects similar to Top1 activity.

b) Premature termination

A potential explanation for the 5′ bias in the RNAPI CRAC profile was premature termination. RNAPII is known to undergo transition from initiation state to elongation state that is associated with changes of phosphorylation status of C-terminal (Milligan et al., 2016). We considered that RNAPI might undergo a similar transition, with the region of the 5′ bias reflecting a region in which RNAP has an elevated probability to terminate. Application of premature termination recapitulates the overall shape of the profile but greatly reduces the total number of RNAP per transcription unit (Figures S4G and S4H). We were unable to find a probability where both criteria, (i) overall profile and (ii) number of RNAP molecules per rDNA, were satisfied. Matching the 5′ bias was accompanied by a 30% lower number of RNAPI molecules per rDNA than observed using Miller spreads. Nevertheless, cannot exclude premature termination of RNAPI or at least partially playing role in establishing the 5′ bias. However, from our modeling, it does not appear to be a key factor.

c) R-loops

R-loops arise when nascent RNA hybridizes with melted DNA helix and constrain progression of RNAP. To include r-loops as a parameter of transcription elongation model their length and position would have to be established. The distribution of RNA-DNA hybrids has been mapped, genome-wide by methods using anti-RNA:DNA antibody (El Hage et al., 2014; Wahba et al., 2016). The median length of r-loop prone genomic regions in yeast was reported to be 500 nt (Wahba et al., 2016), but is unlikely to be the length of actual DNA:RNA hybrids within the rDNA region. Nascent pre-rRNA is co-transcriptionally bound and processed by a multi-protein complex, so called small subunit processome (Turowski and Tollervey, 2015). Only short fragments of free, nascent pre-rRNA are expected to be available for potential RNA:DNA hybrid formation, making the availability of single stranded, nascent RNA rate-limiting. This availability will be anti-correlated to folding energy of nascent RNA extruded from the RNAP; i.e., hairpins within the 5′ETS region should also reduce formation of r-loops. In consequence, the potential for r-loop formation is indirectly implemented into the model by a RNA folding element and there is no need to introduce an additional factor.

Numerical convergence of the model

The stochastic model contains three numerical parameters: the time step, the total time for each independent simulation and the number of independent simulations to be averaged.

The principle constraint on the time step is that the distance moved in a single time step should be 0, 1 or -1 (since only single nucleotide jumps are permitted). As an estimate, we note from Figure S4B' that the probability of sampling a velocity larger than 120 $nt \cdot sec^{-1}$ is very small. We hence take an initial time step estimate of 1/120 $\sim$0.008. We performed test simulations at this and half the time step (0.004) and noted that there were no significant differences. All remaining simulations were performed with this time step.

We determined the total time necessary to run the model by monitoring expected values, such as the number of particles and the mean separation, requiring that these had reached equilibria. The main purpose of this was to remove bias caused by initiation of the RNAP molecules along the transcription unit. We found no significant differences when the total time was between 1500 s and 3000 s.

Increasing the number of independent simulations decreases the statistical noise in the final result. This can also be achieved by increasing the total time of each simulation, but due to parallelization, it is more efficient to increase the number of simulations. We performed convergence studies for a range of parameters and determined that there is no significant difference between results with 256, 512, and 1024 independent simulations. For the parameter studies below, due to the large number of parameter combinations, we used 256 simulations, whereas for the single chosen parameter set we used 1,024.

Model optimization

The model was optimized toward two major criteria: (1) The number of RNAPI molecules present on the transcription unit (Figure S4I). (2) The general shape of the occupancy plot relative to that obtained with CRAC (Figure 1E).

Given the constraints on the parameters discussed above, we tested all parameter combinations with transcription initiation (addProb) = {0.7,0.8,0.9}11, DNA stiffness constant c = {400,500,600}, $dG^{Structure}_{Strength}$ = {1,1.25,1.5}, $dG^{RNA:DNA}_{Strength}$ which was represented as a ratio to $dG^{Structure}_{Strength}$, with ratio = {0.32,0.48,0.64}, threshold value of folding energy (structure2consider) = {-10,-11,-12}. This gave us a total of $3^5 = 243$ sets of parameters, with each varying approximately 10%–20% from the chosen value. Figure S4J demonstrates that the main features of the results (shape and position of peaks, general profile, number of particles), are robust under these variations in the parameters. We also demonstrate that the chosen parameters give a representative dataset, lying approximately in the middle of the set of simulations over all parameters.

Data sampling

In order to mimic the experimental measurement process, we applied a smooth cutoff function to the data, essentially reducing the measurement of RNAPI in areas of low density/high velocity. The cutoff function is given by

$$rho_{Exp} = cutOff \cdot rho$$

$$cutOff = 0.5\left[1 + \mathrm{erf}\left(\frac{(rho - rho_0)}{\sigma}\right)\right]$$

where rho_0 and σ are parameters that determine the *cutOff* position and range. We note that the *in silico* density profiles are normalized so that they have unit area; they are probability distributions. To maintain this, the ‘experimental’ densities are renormalized after the *cutOff*.

Relative contribution of model elements

To calculate the relative contributions of different forces to the modeled elongation, absolute values were used. RNA structures always act positively, RNA:DNA hybrids act negatively, whereas DNA Torsion can act both, positively or negatively. All three modifiers were summed for each nucleotide position and their relative contributions were calculated as a percent of that sum.

QUANTIFICATION AND STATISTICAL ANALYSIS

Pre-processing and data alignment

Illumina sequencing data were demultiplexed using in-line barcodes and in this form were submitted to GEO. First quality control step was performed using FastQC software (http://www.bioinformatics.babraham.ac.uk/projects/fastqc/) considering specificity of CRAC data. Raw reads were collapsed to remove PCR duplicates using FASTX-collapser v0.0.14 (http://hannonlab.cshl.edu/fastx_toolkit/) then inline barcodes were removed using pyBarcodeFilter.py script from pyCRAC package v3.0 (Webb et al., 2014). The 3′ adaptor were removed using flexbar v3.4.0 (Dodt et al., 2012) with parameters -at 1 -ao 4 –u 3, and filtered to retain only reads containing the 3′ adaptor.

All datasets were aligned to the yeast genome using Novoalign v2.07.00 (http://www.novocraft.com) with –r random and saved in novo or sam file format. Second quality control step was performed using pyReadCounters script (pyCRAC package) which calculates overlaps between aligned cDNAs and yeast genomic features. The 3′ end or the 5′end of reads were selected using in-house *awk* script and 1 nt resolution BigWig files were generated using bamCoverage v3.1.3 script from deepTools package (Ramírez et al., 2016). Sam file operations were performed using SAMtools v1.9 (Li et al., 2009).

RNA polymerase I profile

Downstream analyses were performed using python 2.7 Jupiter notebooks, python libraries (pandas v0.19.2, numpy v1.16.0, scipy v1.2.0, matplotlib v2.2.3) and in-house scripts submitted as an update of gwide toolkit v0.5.27 (https://github.com/tturowski/gwide; Turowski et al., 2016). All reads mapping to the gene encoding pre-rRNA (*RDN37* gene with 300 nt overhangs) were summed up to 1 and fraction of reads was used further, adding 10^{-7} pseudo count. There are two copies of the *RDN37* gene in the reference genome; *RDN37-1* and *RDN37-2.* Subsequent analyses used the *RDN37*-1 gene. For simplicity, this is referred to as RDN37 in the text.

The data at 1 nt resolution were quite noisy and we therefore smoothed them with centered Blackman function (window 10). CRAC profiles were presented similar to boxplots of six biological replicates (Figure S1L): median as a solid line, range between second and third quartile with darker color and range between minimum and maximum as lighter color. The basic profile of RNAPI CRAC was established on the basis of six independent biological and technical replicates performed by two different researchers (TWT and EP).

The data were randomized to compare obtained profile with random distribution of similar data, especially within part of 25S rRNA. To generate random data raw reads were shuffled using random functions (shuffled - numpy.random.permutation, choice - numpy.random.choice) and post-processed (calculating fraction of reads, smoothing). Spearman correlations for independent generation of randomized datasets confirmed that distribution is non-random (Figure S1M).

Profile analysis: peak/trough calling and metaplots

Peak/trough calling was performed using argrelextrema function from signal processing library scipy.signal (v1.3.0) using order value most appropriate to applications: 50 for comparison between experiments, 35 for comparison with folding energy and 20 for analysis of GC-richness. To generate peak/trough metaplot for each peak or trough two sided window around the feature was superimposed with all other peaks. Mean for all windows were calculated and data for each dataset were presented as peak/trough metaplot (Figure S1F).

For RNAPII analysis, due to different length of transcripts, reads were additionally normalized to fraction of reads in the window followed by calculation of mean. For each position an average of folding energy was calculated for a 40 nt window around each feature. Wilcoxon signed rank test was applied.

CRAC versus PAR-CRAC comparison

To investigate differences between CRAC and PAR-CRAC their normalized *RDN37* profiles were subtracted from each other and regions where the difference was ≥ 0.0005 were treated as specific for UVA (PAR-CRAC) or UVC (CRAC). For all specific positions an average frequency of nucleotides in a short (1 to 15 nt) window was calculated and two tailed student test was applied. p value $<$ 0.005 was found for windows 1 to 3 nt.

Analysis of G+C-richness

Content of guanine (G) or cytosine (C) bases in a given window is called G+C-richness. A given peaks or troughs G+C-richness was calculated depending on application using window: 10 nt upstream, 10 nt downstream or 10 nt upstream plus 10 nt downstream. P values were calculated using two-sided t test.

Folding of nascent RNA

Each sequence was divided into segments using a rolling window of *w* nt, where *w* was the length of RNA considered to form structure (chosen 65 nt, tested range 10-80 nt). The folding energy at 30°C was calculated using hybrid-ss-min from UNAfold package v3.8 (Markham and Zuker, 2008). Folding energies were associated with the position of last nucleotide in the sequence and off set was applied (chosen 15 nt, tested range 0-80 nt). The offset aimed to exclude the 3′ end of the nascent RNA immersed in the RNAP complex and calculate folding energy only for the extruded RNA. The folding energy for each position was converted to BigWig files. The data for the 65 nt window are provided as Data S1. The BigWig files for all other windows tested (10 – 80 nt at 5 nt intervals) are available from the authors.

Analysis of splicing

Analysis of splicing speed-ranked genes used published data (Barrass et al., 2015). Genes were selected as previously described. Non-ribosomal, protein coding genes were sorted according to their AUC value and 1/3 of fastest (fast) and 1/3 of slowest (slow) genes were selected.

For analysis of the 3′ SS selection in yeast the features of known introns were extracted as described (Pleiss et al., 2007) using the MEME suite (Bailey, 2011; Machanick and Bailey, 2011). The following algorithm was implemented to predict introns *de novo*: (1) find all branch points (BP), (2) for each BP find the 5′ SS, upstream to the BP and non-overlapping with it, (3) find the 3′ SS at least 4 nt away from the last nucleotide of the BP. This approach was able to predict correctly positions of 236 of 256 annotated yeast introns. For some genes an additional, consensus 3′ SS was predicted but skipped in the spliced product. Only genes where the predicted but skipped 3′SS and the utilized 3′ SS were separated by at least 40 nt were selected for this analysis. Folding energy is presented, normalized to the 3′ SS, to highlight changes in folding as the polymerase moves downstream of this position. P values were calculated using Wilcoxon rank-sum test for ± 10 nt around the position 60 nt downstream of each potential 3′ SS.

Statistical analyses

All plots and statistical analyses of this work were performed using python 2.7 Jupiter notebooks and python library scipy v1.2.0. Wilcoxon rank-sum test was used unless stated otherwise. t test was used for G+C richness analysis (Figures 2B and S2E) and Wilcoxon signed-rank test was used for Figures 6E and 6F. Statistical details can be found in the figure legends, including the statistical tests used, exact value of n and p value.

Boxplots present 2nd and 3rd quartile, line marks median and whiskers range between 5th and 95th percentile.

Molecular Cell, Volume *79*

Supplemental Information

Nascent Transcript Folding Plays a Major Role in Determining RNA Polymerase Elongation Rates

Tomasz W. Turowski, Elisabeth Petfalski, Benjamin D. Goddard, Sarah L. French, Aleksandra Helwak, and David Tollervey

SUPPLEMENTARY FIGURES

Supplementary Figure 1

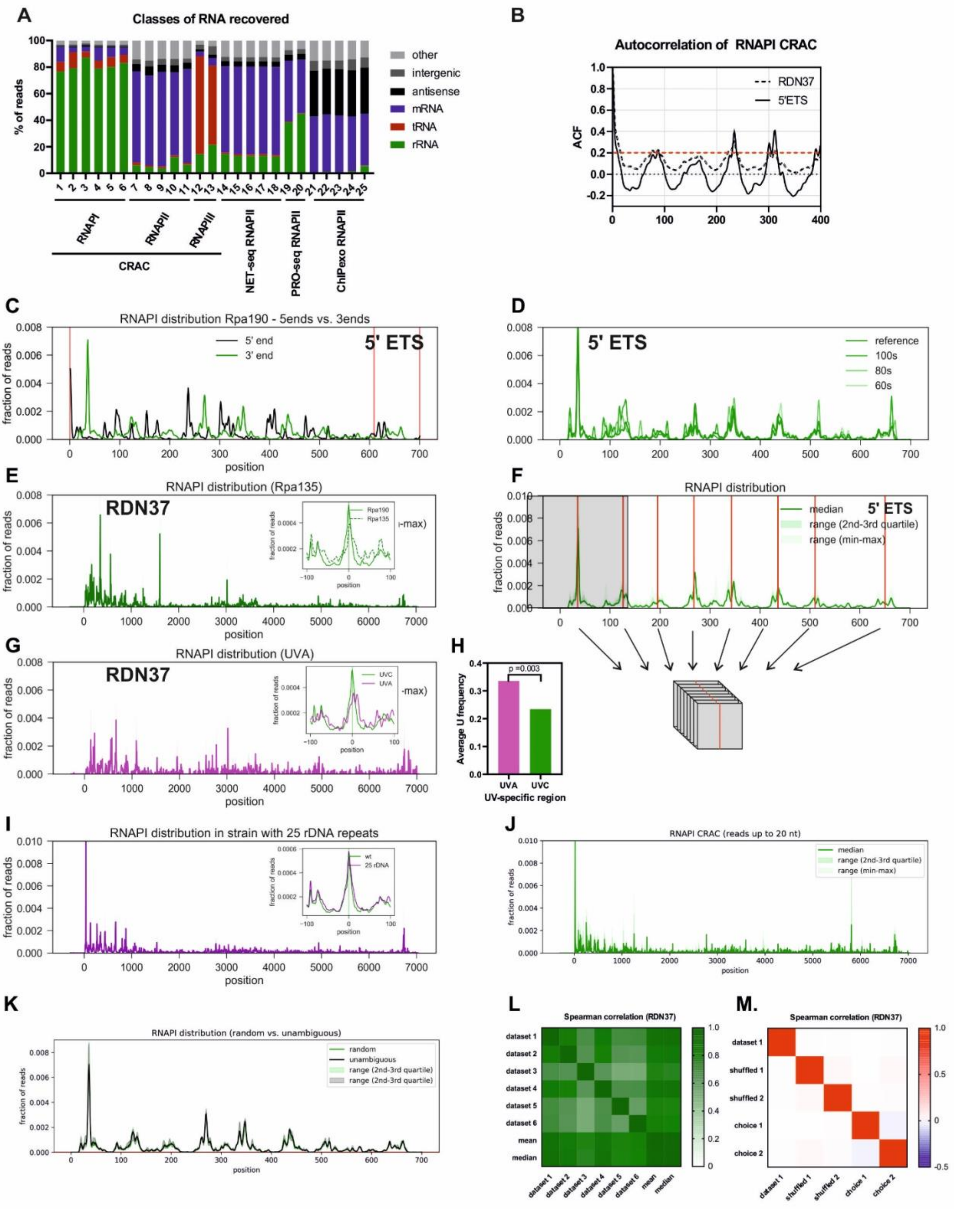

Figure S1. Initial analysis of RNAPI CRAC data (related to Figure 1)

(A) Transcriptome-wide binding profiles for RNAP I, II and III from CRAC (lanes 1 to 13), RNAPII NET-seq (lanes 14-18), PRO-seq (lanes 19-20) and ChIPexo (lanes 21-25).
(B) Autocorrelation function for the 5' ETS region of the pre-rRNA (solid line) and entire *RDN37* transcription unit (dashed line) indicates ~80 nt spacing between peaks in RNAPI CRAC data.
(C) RNAPI CRAC profile over the 5'ETS. The 3' ends (median, green) and the 5' ends (median, black) of mapped RNAs were plotted. There is no clear enrichment of reads at cleavage sites A0 and A1 (red lines).
(D) Rpa190-HTP CRAC profiles for UVC cross-linking time course. The 5' ETS region is presented to show data reproducibility for 60, 80 and 100 seconds of UVC cross-linking time.
(E) Rpa135-HTP CRAC profile, with the second largest RNAPI subunit tagged. Embedded: RNAPI CRAC peak metaplot for *RDN37*, comparing Rpa190 and Rpa135 at the level of single peaks.
(F) Outline of peak metaplot generation. All peaks were found using a dedicated function (see Materials and methods). Windows around each peak were superimposed to generate a metaplot centered around the peaks.
(G) Rpa190-HTP PAR-CRAC (UVA) profile. Embedded: Rpa190-HTP CRAC (UVC) peaks metaplot for *RDN37* overlaid with Rpa190-HTP PAR-CRAC (UVA). The data shows reproducibility on a level of single peaks.
(H) Average U frequency is slightly higher at the 3' end of reads recovered with PAR-CRAC. U frequency was calculated for the last two nucleotides for regions specifically enriched with UVA (purple) or UVC (green). P-value was calculated using a two-sided T-test.
(I) Rpa190-HTP CRAC profile for *RDN37* obtained from strain with 25 rDNA repeats. Embedded: Rpa190-HTP CRAC peaks metaplot for *RDN37* comparing wt and 25 rDNA repeat strains at the level of single peaks.
(J) Rpa190-HTP CRAC profile for *RDN37* obtained from reads only up to 20 nt.
(K) Comparison of Rpa190-HTP CRAC profiles for *RDN37* using random and unambiguous mapping.
(L) Spearman correlation between datasets 1-6, and median or mean of these datasets.
(M) Spearman correlation between dataset 1 and randomized datasets.

Supplementary Figure 2

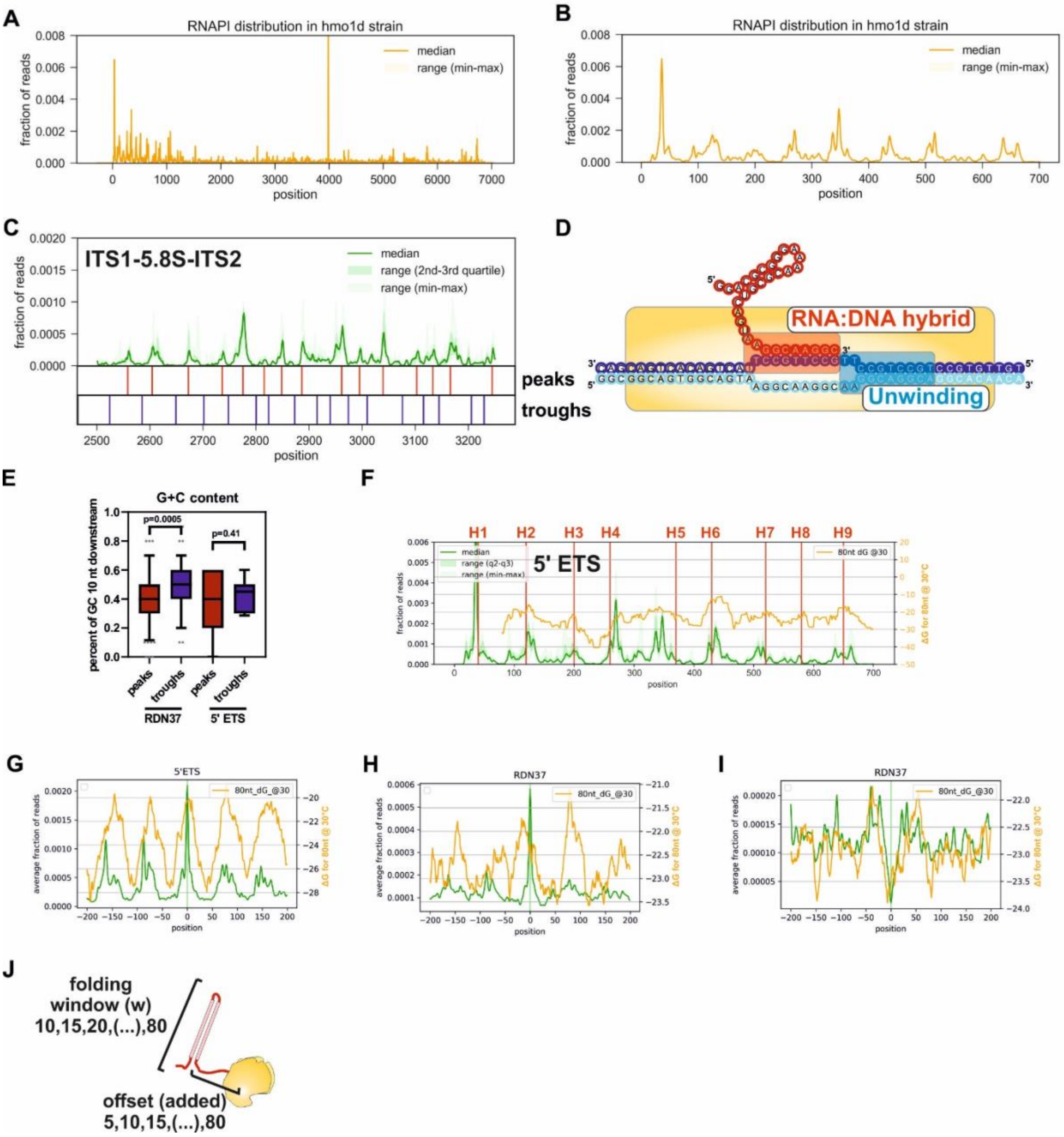

Figure S2. RNAPI density correlates with features in the nascent pre-rRNA (related to Figure 2)

(A) RNAPI CRAC profile for *RDN37* from the Rpa190-HTP *hmo1Δ* strain. The peak at 4000 nt is an artefact previously observed in CRAC analyses.

(B) RNAPI CRAC profile for 5' ETS from the Rpa190-HTP *hmo1Δ* strain.

(C) RNAPI CRAC profile for the ITS1, 5.8S rRNA and ITS2 regions with marked peaks and troughs (identified by peak calling algorithm and) used for further analysis.

(D) Schematic representation of nucleotides considered to play role in RNA:DNA hybrid formation (red box) or DNA unwinding (blue box).

(E) G+C-content in front of RNAPI has a minimal impact on DNA unwinding. Boxplot presenting distribution of G+C-content among peaks and troughs within RDN37 or the 5' ETS. G+C content is calculated for 10 nt downstream from each feature.

(F) RNAPI CRAC profile (green) for the 5' ETS and folding of the nascent transcript using different 80 nt folding windows at 30°C. Folding energy was calculated using hybrid-ss-min from the UNAfold package. Red vertical lines represent positions of the known hairpin loops (numbered).

(G) RNAPI CRAC peaks metaplot for the 5' ETS with folding energy of the nascent transcript (80 nt folding window). *Note*: Stronger structures have lower ΔG.

(H) RNAPI CRAC peaks metaplot for *RDN37* with folding energy of nascent transcript (80 nt folding window).

(I) Same as (H) but troughs were superimposed.

(J) Schematic representation of all parameters tested for calculation of folding window (10-80 nt) and offset (5-80 nt).

Supplementary Figure 3

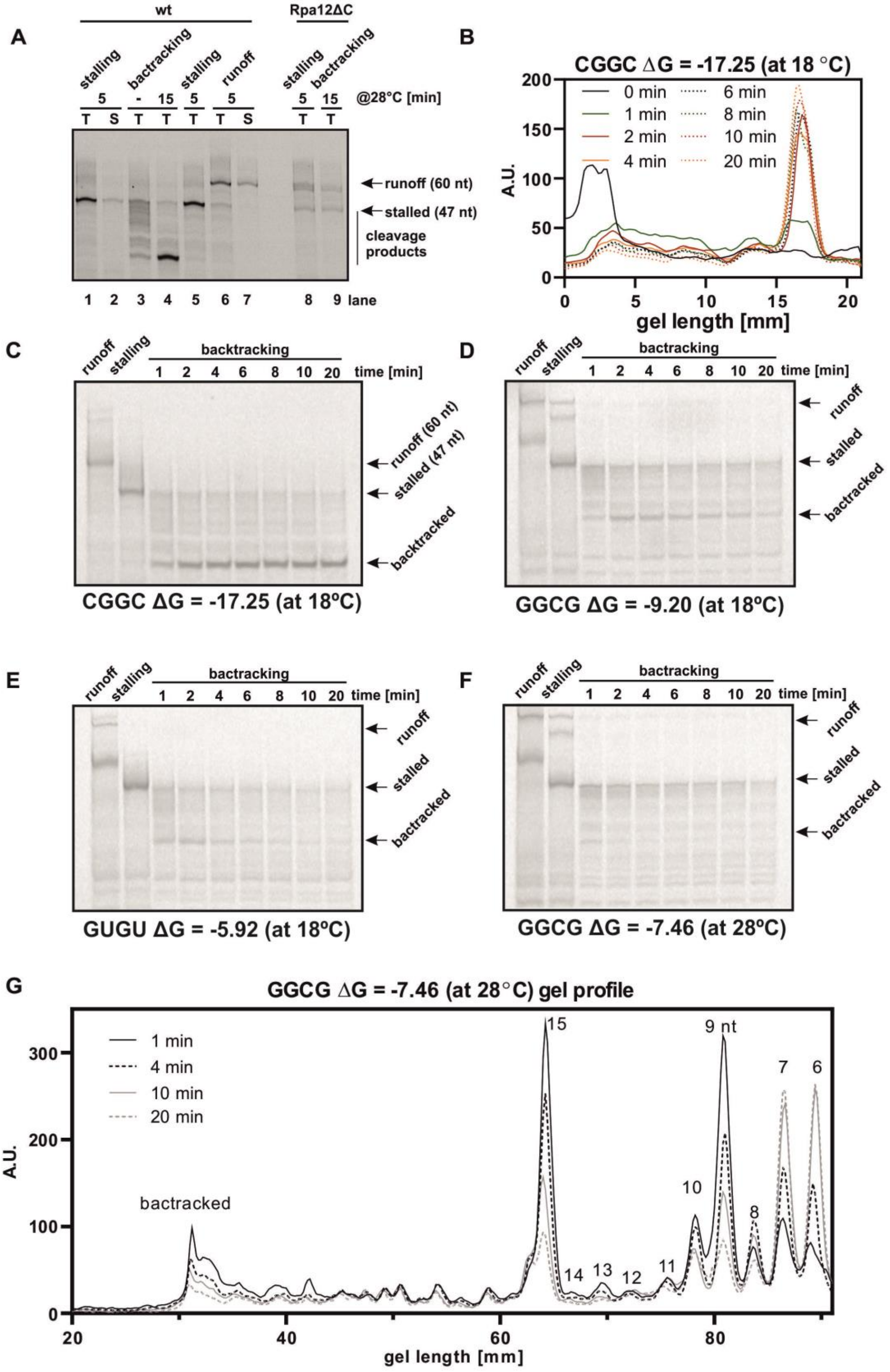

Figure S3. Nascent RNA limits backtracking proportionally to folding energy (related to Figure 3)

(A) RNAPI cleavage requires the Rpa12 C-terminal domain. *In vitro* assay for RNAPI backtracking, performed after initial incubation with transcription buffer (TB) lacking ATP (“stalling”). Nucleotides were washed out and samples incubated in TB without NTPs to induce RNAPI backtracking (“backtracking”). Cleavage products were detectable for RNAPI with wild type Rpa12, but were not observed with Rpa12ΔC. Unexpectedly, the C-terminal truncation of Rpa12 allowed substantial readthrough of the AAA stalling sequence during extension in the absence of added ATP (stalling, lane 8). This observation will be followed up elsewhere.

(B) Quantification of all lanes from Fig. 3E presenting area between stalled and backtracked peaks.

(C) – (F) Representative gels showing backtracking assays used to study kinetics of RNAPI translocation. Assay performed as in Fig. 3C. The scaffold used, temperature and predicted folding energy are indicated below each panel.

(G) Quantification of backtracked peaks and shorter products from backtracking assay. For clarity, only four time points are displayed. The scaffold used, temperature and predicted folding energy are indicated above the plot.

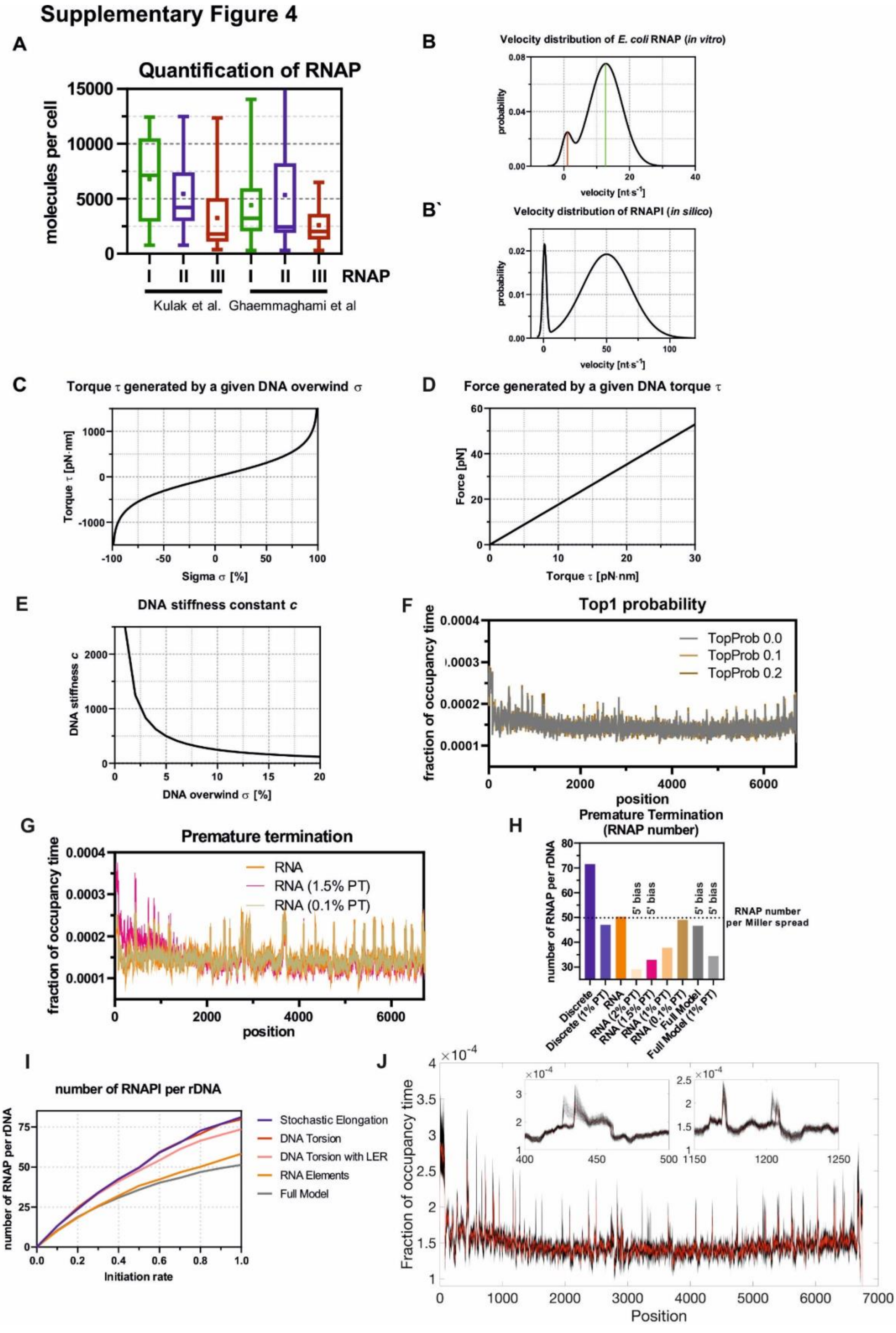

Figure S4. Mathematical model of RNAPI transcription (Related to Figure 4)

A: Quantification of RNA polymerases in yeast cell. Boxplots for each RNA polymerase using two independent studies and all detected subunits.
B: Velocity distribution of bacterial RNAP (Adelman et al., 2002). Described by two Gaussian functions: reflecting 7.8% of the area, represents paused state and is centered at 0.9 $nt \cdot s^{-1}$ and a second function, which reflects active elongation and is centered at 12.8 $nt \cdot s^{-1}$.
B': Same as B, but the active elongation function is centered at 50 $nt \cdot s^{-1}$.
C: Function of DNA torque τ generated by a given DNA overwind σ.
D: Function of force F generated by a given DNA torque τ.
E: Function of DNA stiffness constant c in relationship to DNA overwind σ for V_{Int}=50 $nt \cdot s^{-1}$.
F: Effect of Top1 (n=64). The results of simulations using parameter TopProb = {0, 0.1, 0.2} were superimposed to show high similarity of overall profile.
G: Effect of premature termination (n=64). The results of simulations allowing for premature termination over first 2000 nt with a given probability = {0%, 0.1%, 1.5%} were superimposed to show high similarity of overall profile.
H: Number of RNAPI molecules per rDNA relative to the premature termination rate (panel Fig. S5G) Note: The 5' bias of the profile (panel I) is accompanied by ~35% drop of RNAP molecules, whereas stable number of RNAPI molecules do not recapitulate the 5' bias (panel J).
I: Number of RNAPI molecules per rDNA relative to the initiation rates are shown for different version of the model used in Figs. 4C-4G.
J: Parameter Fitting (simulations n=256). The results for each parameter set are plotted in (transparent) grey; darker areas indicate more likely results. The red line denotes our choice of parameters (simulations n=1,042). Insets show two zoomed areas. Note that the results are robust under changing parameters, and the results presented for the chosen parameter set are 'typical'.

Supplementary Figure 5

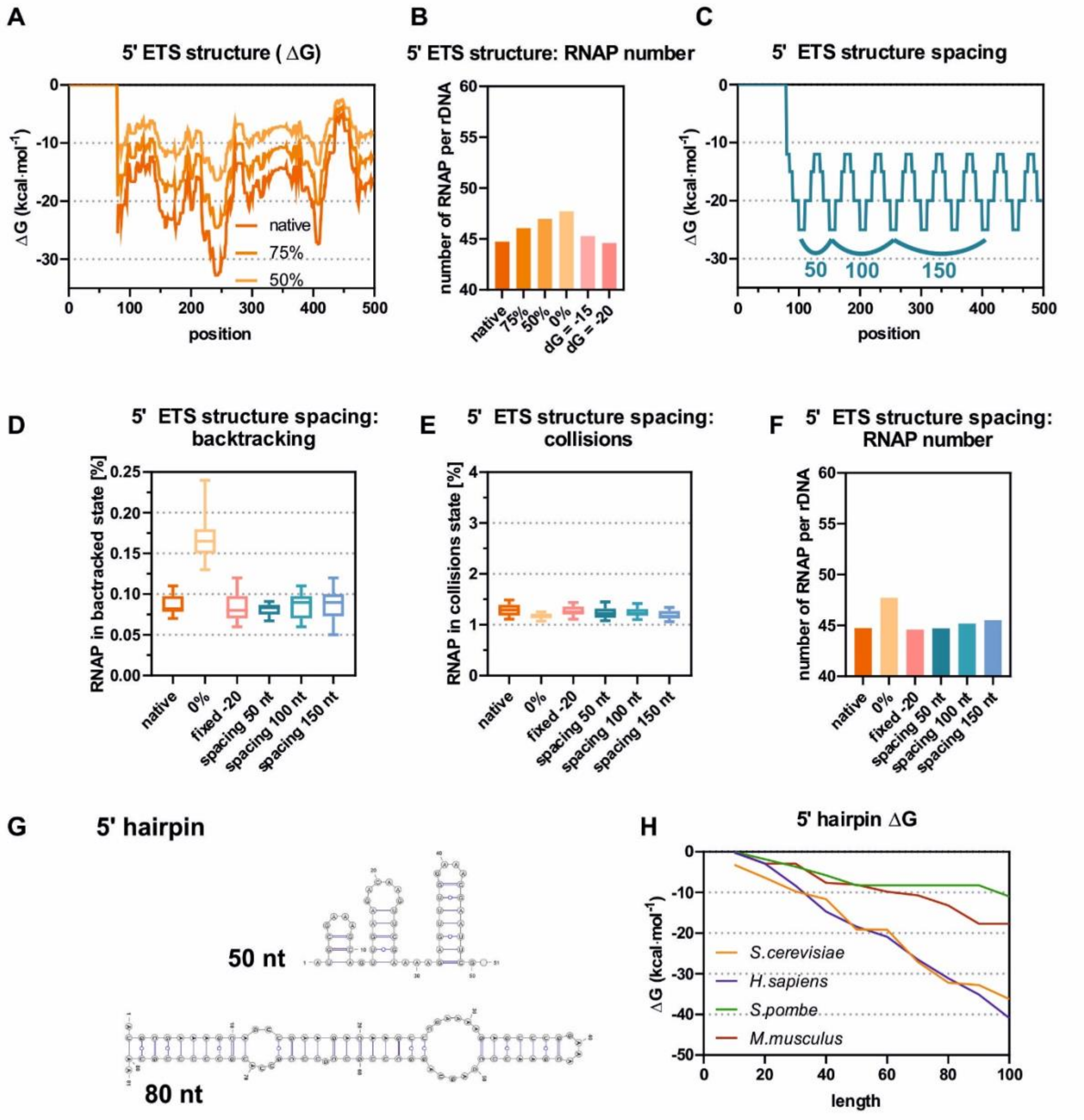

Figure S5. Conclusions from the model on the role of 5’ ETS structure (Related to Figure 5)

A: Plot of the folding input data for the first 500 nt. Folding energy of the 5’ ETS was calculated for a rolling window of 65 nt with 15 nt offset (dark orange). Two modified input files are presented, with folding reduced to 75% or 50% of the biological values. The input with a fixed ΔG would be a straight line and is omitted for clarity.

B: The predicted number of RNAPI molecules loaded on the rDNA remains similar for altered 5’ ETS structures.

C: Overview of altered spacing of 5’ ETS hairpins. Structural data were generated as presented with peak spacing equal 50. For 100 and 150 nt spacing, peaks were connected using longer spacers of fixed energy ΔG = -12 kcal·mol-1.

D-F: Modification of spacing between hairpins within the 5'ETS. Measured features of the model remain unaffected: frequency of backtracking (D), frequency of collisions (E) and RNAP number per rDNA unit (F).
G: Secondary structures of the 5' proximal hairpin in the 5' ETS (lower panel) and the 50 nt long transcription intermediate (upper panel).
H: Folding energy of the full-length 5' proximal hairpins and transcription intermediates. The dG was calculated for first 100 nt of the 5' ETS and for shorter intermediates at 10 nt intervals, using sequences from *S. cerevisiae, H. sapiens, S. pombe* and *M. musculus*.

Supplementary Figure 6

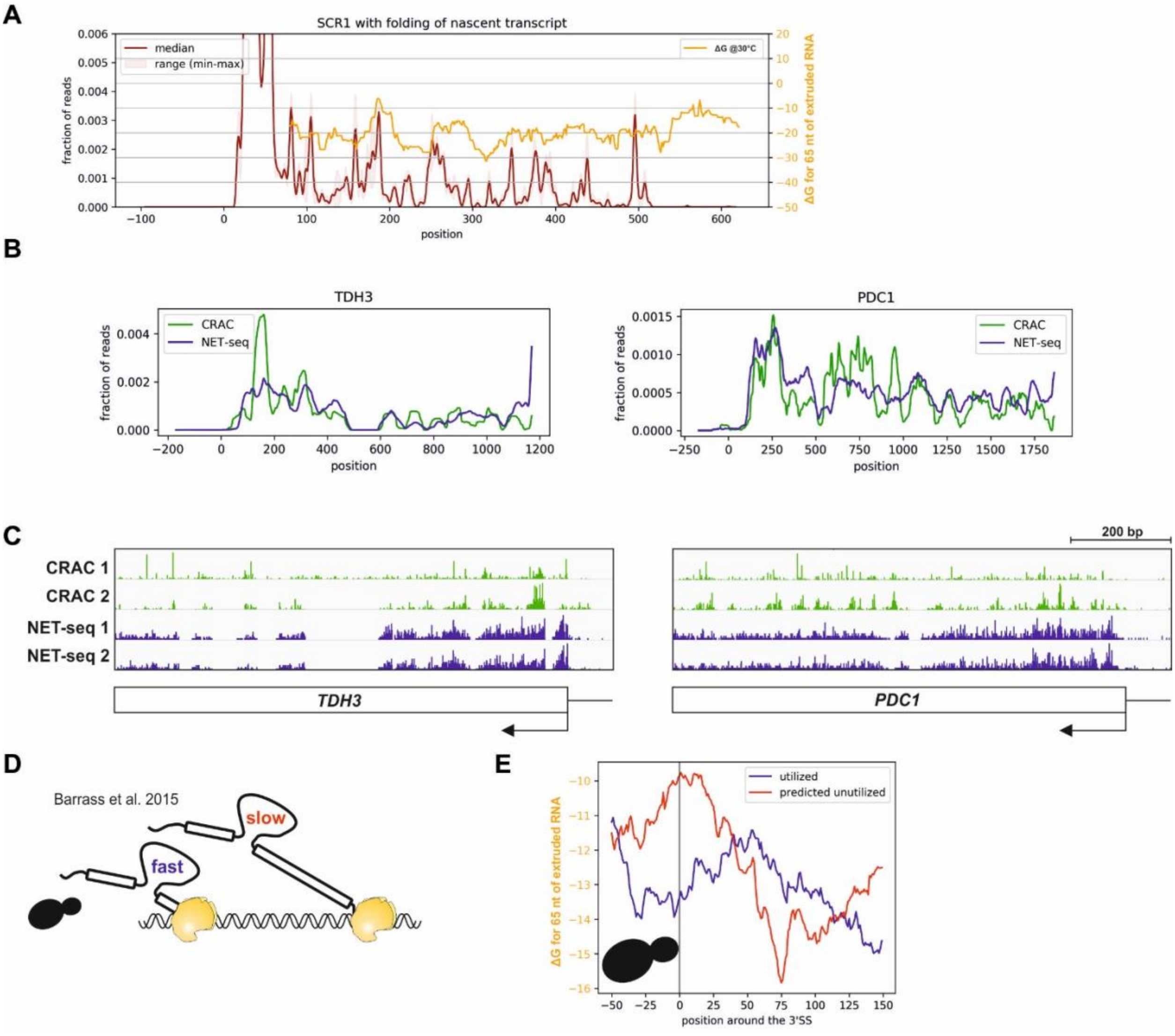

Figure S6. Folding of the nascent transcript plays a role in determining elongation rate of all eukaryotic RNA polymerases (Related to Figure 6)

A: RNAPIII profile for *SCR1* overlaid with nascent RNA folding. The same folding window as for RNAPI analysis was used (65 nt rolling window with 15 nt offset).

B: RNAPII density across *TDH3* (left panel) and *PDC1* genes (right panel). Medians of NET-seq (blue) and CRAC (green) data were plotted to show similarities between the profiles.

C: Genome Browser tracks for RNAPII CRAC (green) and NET-seq (blue) for two highly expressed genes *TDH3* and *PDC1*. Two replicates are shown.

D: Cartoon indicating differences between fast and slow-spliced pre-mRNAs. Slow splicing could be also considered as post transcriptional.

E: Folding energy of nascent RNA around utilized 3' SS, versus predicted but skipped 3' SS. The same data were used to generate Fig. 6I. Note that folding of the nascent RNA does not affect accessibility of the 3' SS.

SUPPLEMENTARY TABLES

Table S1. Fixed parameters used in the mathematical model of RNAPI transcription (related to STAR Methods)

Name	value	Reference
Mean elongation of RNAPI	40 nt s^{-1}	(Kos and Tollervey, 2010)
35S pre-rRNA transcription time	170 s	(Kos and Tollervey, 2010)
rDNA repeats	150-200	(Nomura, 1999)
Active rDNA repeats	75-100	(Toussaint et al., 2005)
RNAPI per rDNA	50±2.5	(Dasgupta et al., 2007; El Hage et al., 2010; French et al., 2003; Hontz et al., 2008; Oakes et al., 2006; Sandmeier et al., 2002; Schneider et al., 2006; Tongaonkar et al., 2005; Viktorovskaya and Schneider, 2015)
35S rDNA length	~7000	SGD
RNAPI footprint	38 nt	(Neyer et al., 2016) PDB 5M5X

Table S2. The velocity of RNA polymerases (related to STAR Methods)

RNA polymerase	Method	Elongation rate nt sec^{-1}	Reference
RNAPII human	single cell microscopy, *in vivo*	115	(Tantale et al., 2016)

RNAPII yeast	single molecule transcription assay, *in vitro*	25-30	(Dangkulwanich et al., 2013)
RNAPII yeast	single molecule transcription assay, *in vitro*	18.7 +/- 2.7	(Lisica et al., 2016)
RNAPI yeast	single molecule transcription assay, *in vitro*	32.2 +/- 2.5	(Lisica et al., 2016)
RNAPI yeast	Metabolic labelling, *in vivo*	40	(Kos and Tollervey, 2010)
RNAPII human	4sUDRB-seq, *in vivo*	30-100	(Fuchs et al., 2014)
RNAPII human	DRB-arrest release, *in vivo*	50	(Cortazar et al., 2019)
RNAPII human	CDK9 inhibition and TT-seq, *in vivo*	39	(Gressel et al., 2017)
RNAPII mouse	Transcription release and GRO-seq, *in vivo*	33	(Jonkers et al., 2014)
